# Identification of an NAC Transcription Factor Family by Deep Transcriptome Sequencing in Onion (*Allium cepa* L.)

**DOI:** 10.1371/journal.pone.0157871

**Published:** 2016-06-22

**Authors:** Xia Zheng, Shouwei Tang, Siyuan Zhu, Qiuzhong Dai, Touming Liu

**Affiliations:** Institute of Bast Fiber Crops and Center of Southern Economic Crops, Chinese Academy of Agricultural Sciences, Changsha, China; Youngstown State University, UNITED STATES

## Abstract

Although onion has been used extensively in the past for cytogenetic studies, molecular analysis has been lacking because the availability of genetic resources is limited. NAM, ATAF, and CUC (NAC) transcription factors (TFs) are plant-specific proteins, and they play key roles in plant growth, development, and stress tolerance. However, none of the onion NAC (CepNAC) genes had been identified thus far. In this study, the transcriptome of onion leaves was analyzed by Illumina paired-end sequencing. Approximately 102.9 million clean sequence reads were produced and used for *de novo* assembly, which generated 117,189 non-redundant transcripts. Of these transcripts, 39,472 were annotated for their function. In order to mine the CepNAC TFs, CepNAC genes were searched from the transcripts assembled, resulting in the identification of all 39 CepNAC genes. These 39 CepNAC proteins were subjected to phylogenetic analysis together with 47 NAC proteins of known function that were previously identified in other species. The results showed that they can be divided into five groups (NAC-I–V). Interestingly, the NAC-IV and -V groups were found to be likely related to the processes of secondary wall synthesis and stress response, respectively. The transcriptome analysis generated a substantial amount of transcripts, which will aid immensely in identifying important genes and accelerating our understanding of onion growth and development. Moreover, the discovery of 39 CepNAC TFs and the identification of the sequence conservation between them and NAC proteins published will provide a basis for further characterization and validation of their functions in the future.

## Introduction

Transcription factors (TFs) are proteins that regulate gene expression by binding specific DNA sequences in the regulatory region of target genes, thereby modulating RNA polymerase activity for the initiation of transcription. NAM, ATAF, and CUC (NAC) TFs are one kind of proteins that only exist in plant. The name of this family was originally derived from the names of three proteins: no apical meristem (NAM), ATAF1-2, and CUC2 (cup-shaped cotyledon) [1, 2]. NAC TFs have a highly conserved DNA-binding domain in the N-terminal region and a variable functional domain in the C-terminal region [3, 4]. Generally, this variable C-terminal domain can function as transcriptional activator or repressor [5–7]. The NAC family is a large gene family and comprises a large number of protein members in plants, and at least 151, 117, 163, and 101 non-redundant NAC TF protein members have been identified so far in rice, *Arabidopsis*, *Populus*, and soybean genomes, respectively [8–10].

At present, there are at least 50 NAC genes that have been functionally characterized [11, 12, 13], and they are involved in many aspects of plant growth and development, including embryo development, seed germination, lateral root development, maintenance of the shoot apical meristem, flower formation, plant immune response, cell division and growth, senescence, and formation of secondary walls [3, 11]. Moreover, NAC TFs were also found to participate in regulating of plant tolerance to abiotic/biotic stress [14, 15]. Therefore, NAC family is multifunctional proteins family with various roles in regulating plant growth and development.

Onion (*Allium cepa*) is one of the most important vegetable crops of the *Allium* family; it is known for its nutritional and medicinal properties and has been widely planted around the world with more than 5000 years [16]. Onion is a diploid (2n = 16) plant and has a large nuclear genome of approximately 16.4 Gb per 1C, which is about 38 and 130 times larger than the rice and *Arabidopsis* genomes, respectively [17, 18]. Molecular analysis in onion has been hindered by the limited availability of genetic resources, as a result of the onion’s large nuclear genome, which presents challenges for gene identification. Therefore, while the onion has been used extensively in previous studies for cytogenetic analysis, the molecular analysis of onion genes has been lacking. In recent years, the rise of next generation sequencing (NGS) technologies has offered a powerful and cost-efficient tool for high-throughput sequence determination. Transcriptome analysis based on this technology has led to the discovery of new genes in hundreds of species [19–23]. However, no studies to date have identified NAC family genes in onion.

In the present study, we have *de novo* assembled and characterized the transcriptome of onion leaves using Illumina paired-end sequencing technology and searched onion NAC (CepNAC) genes from the assembled transcripts. Furthermore, we have performed phylogenetic analysis of the CepNAC TFs identified in this study along with 47 NAC proteins of known function that were previously identified in other species (S1 Table). The discovery of these *CepNAC* genes will provide a basis for further understanding of their function in onion growth and development.

## Materials and Methods

### Plant material and RNA extraction

The study included a local variety of onion (Chalinghuangpi) that produces yellow-skin bulbs and was collected from Chaling, Hunan, China. The onion was planted in the experimental field of the Institute of Bast Fiber Crops, Chinese Academy of Agricultural Sciences, Changsha, China, on Sept. 20, 2014. On Mar. 10, 2015, leaves tissues of three individuals were sampled. The tissue sample was immediately frozen in liquid nitrogen, and then stored at −80°C until use. E.Z.N.A. Plant RNA Kit (OMEGA Bio-Tek, USA) was used to extract the total RNA according to the manufacturer’s protocol.

### cDNA library construction, sequencing, and assembly

Illumina sequencing was performed at Novogene Bioinformatics Technology Co., Ltd, Beijing, China (www.novogene.cn). RNA of onion leaves was used to construct the cDNA libraries with fragment lengths of approximately 250 bp (±25 bp). Paired-end sequencing was then performed using the Illumina sequencing platform (HiSeq™ 2500) according to the manufacturer’s instructions (Illumina, San Diego, CA). After trimming adapter sequences and filtering low-quality reads, the clean reads were used to search against NCBI non-redundant protein sequences (NR) database by BLAST (Basic Local Alignment Search Tool) with an E-value threshold of 10^−1^, and the reads that showed significant similarity with other species might be the sequences derived from other species, thereby being removed. Thereafter, the remaining clean reads were used to *de novo* assemble the onion transcriptome using Trinity [24]. Sequencing reads and transcripts assembled are deposited in the NCBI Sequence Read Archive (SRA) and Transcriptome Shotgun Assembly (TSA) Database, under accession number SRA347079 and GEOY00000000, respectively.

### Gene annotation and classification

To annotate the transcripts of onion transcriptome using bioinformatics approachs, each of the assembled transcripts was searched against seven public databases, including KEGG Orthology (KO) database, protein family (PFAM) database, eukaryotic orthologous groups (KOG) database, NR database, NCBI nucleotide sequences (NT) database, Swiss-Prot protein database, and Gene Ontology (GO) database. WEGO software was used to perform GO functional classification of all transcripts to visualize gene functions distribution. Transcripts annotated in the KOG database were classified according to the COG group that each transcript was assigned to, whereas transcripts annotated from the KO database were grouped according to the KEGG pathway that gene was assigned to.

### Identification of NAC genes and phylogenetic analysis

In order to identify the NAC genes, the keywords ‘‘NAC,” ‘‘no apical meristem,” or ‘‘NAM” were used as the query to search against annotations of the onion transcripts assembled. ORFs of identified NAC genes were screened using the findorf software. All non-redundant genes identified were further validated as NAC genes by manually checking for their conserved NAM domain. In addition, information regarding 47 functionally characterized NAC genes was collected from previous studies conducted in other plant species, and can be found in S1 Table. We performed the phylogenetic analysis on these 47 NAC proteins of known function, together with the CepNAC proteins identified in this study. Clustal X (version 1.83) program [25] was used to perform multiple sequence alignments of full-length protein sequences, including the highly conserved N-terminal NAM domain and the more divergent C-terminal activation domain. Neighbor-joining (NJ) method was used to construct an unrooted phylogenetic tree by using MEGA 4.0 [26], and the bootstrap analysis was performed with 1000 replicates. NAC proteins of known function that had closest evolutionary relationship to each CepNAC protein were ascertained based on the phylogenetic tree, and their percentage similarity analyzed using the BLASTP program.

## Results

### Illumina paired-end sequencing and *de novo* assembly

**Fig 1:**
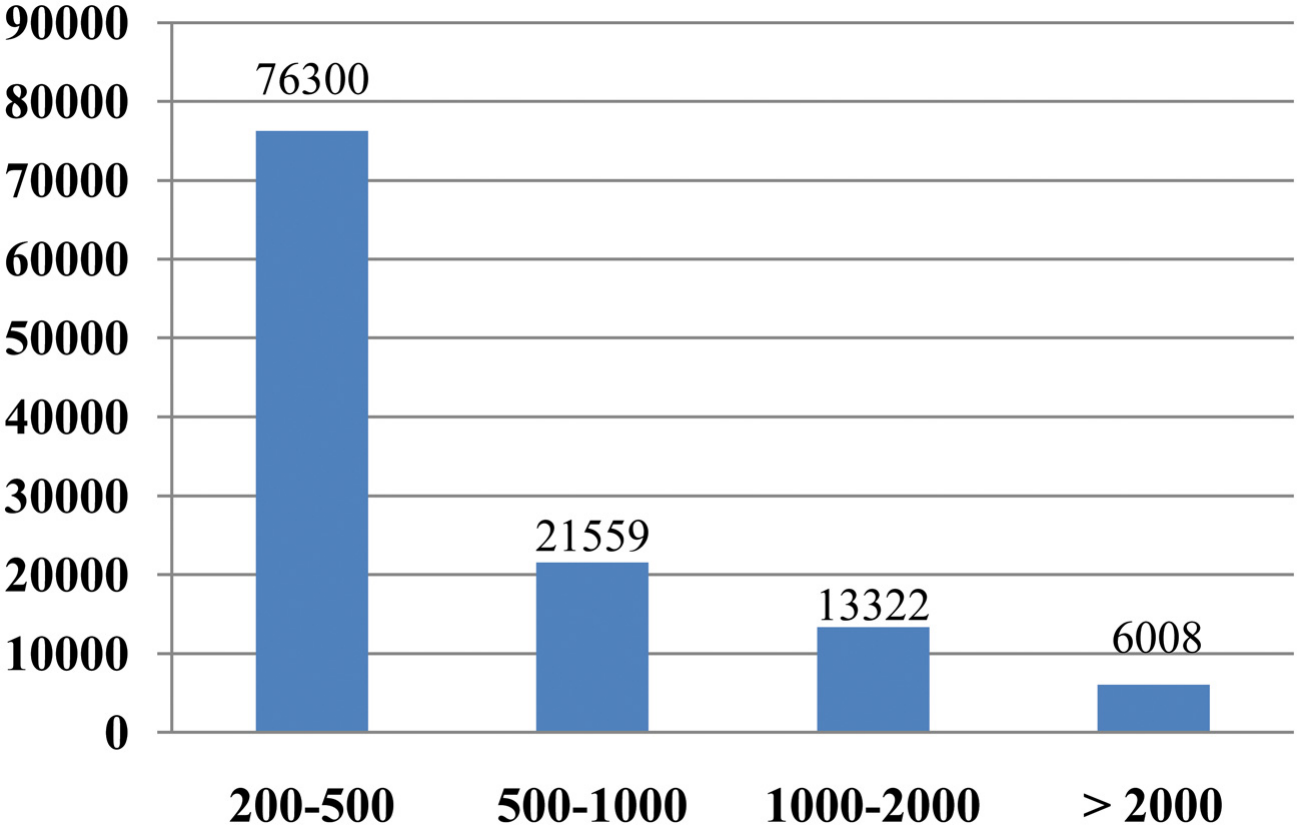
Length distribution of assembled transcripts.

RNA extracted from onion leaves was used to construct a library of cDNA sequences. In all 102.9 million clean sequence reads with lengths of 125 bp were sequenced from the library. Next, these sequence reads were used for the *de novo* assembly using Trinity software. Sequences that were not extended on either end were defined as transcripts. Eventually, a total of 117,189 non-redundant transcripts were assembled. The average length and total length of these *de novo* assembled transcripts were 632 bp and 74.03 Mb, respectively (Table 1). The length was between 500 and 1000 for 21,559 (18.4%) transcripts, between 1001 and 2000 bp for 13,322 (11.4%) transcripts, more than 2000 bp for 6,008 (5.1%) transcripts, and no more than 500 bp for all remaining transcripts (Fig 1).

**Table 1:** Summary of transcriptomes assembled by Illumina sequencing.

Clear reads	102,874,740
Clean bases (Gb)	12.86
Transcript number	117,189
Total length of transcript (Mb)	74.03
Average length of transcript (bp)	632
Transcript number annotated	39,472 (33.68%)

### Functional annotation

**Fig 2:**
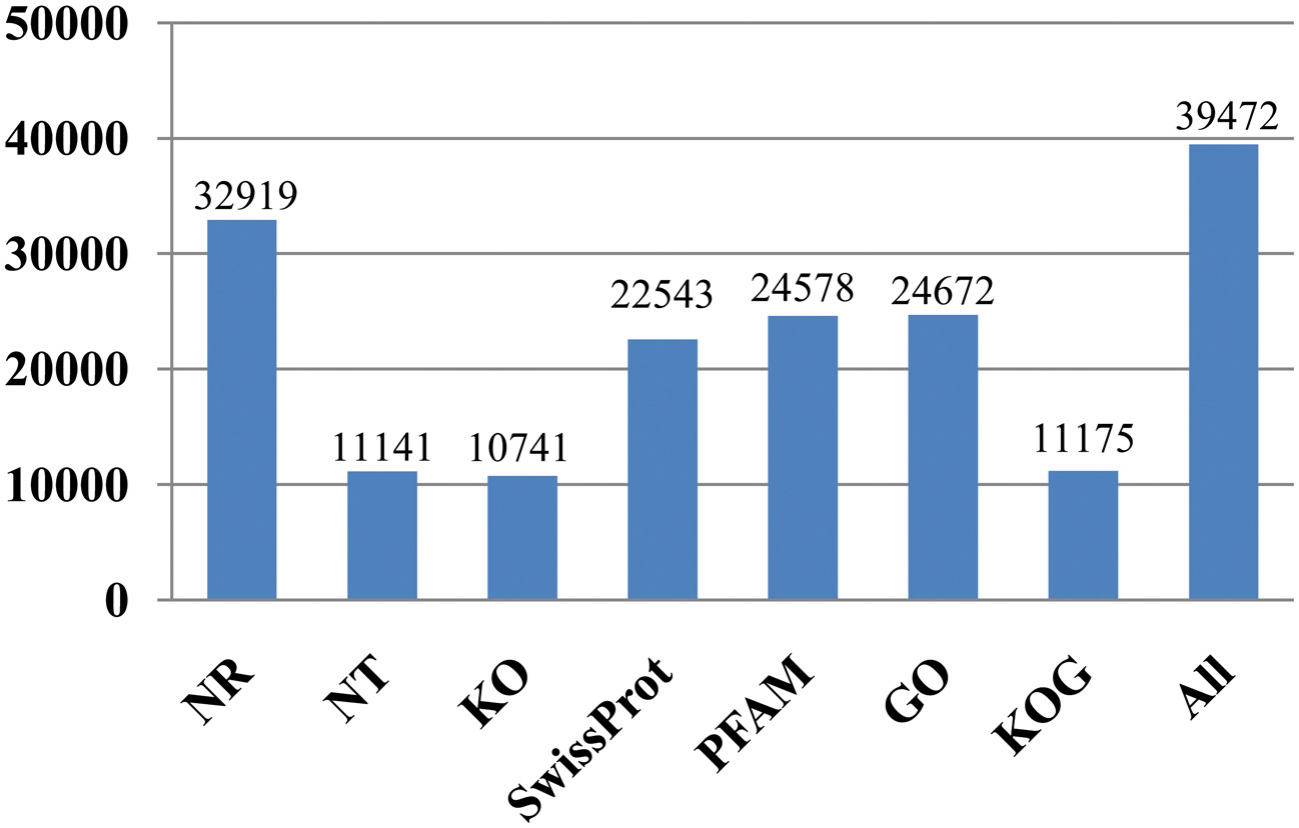
Transcript numbers annotated in the seven public databases searched.

To validate and annotate these assembled transcripts, sequence similarity searches were performed against in seven public databases. Of 117,189 transcripts, 32,919 (28.1%) showed significant similarity to known proteins in the NCBI non-redundant protein sequence (NR) database, 11,141 (9.5%) in the NCBI nucleotide sequence (NT) database, 10,741 (9.2%) in the KEGG orthology (KO) database, 22,543 (19.2%) in the SwissProt database, 24,578 (21.0%) in the protein family (PFAM) database, 24,672 (21.1%) in the Gene Ontology (GO) database, and 11,175 (9.5%) in the eukaryotic orthologous groups (KOG) database (Fig 2). Finally, 39,472 (33.7%) transcripts showed similarity to known proteins in at least one of these seven databases, and were achieved for annotating function.

### Functional classification

**Fig 3:**
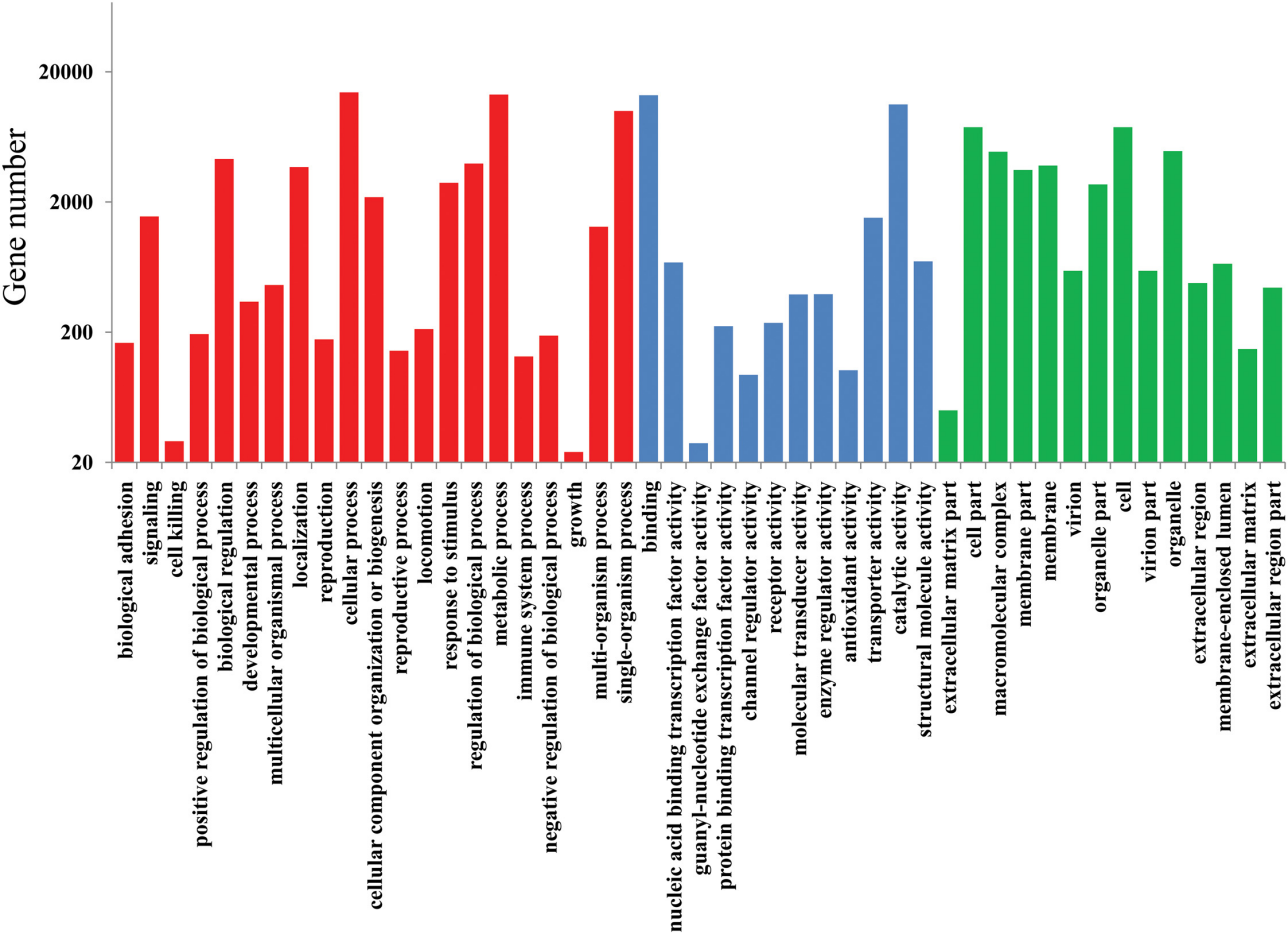
Gene ontology classifications of assembled transcripts; the red, blue, and green histogram bar represented three main categories: biological process, molecular function, and cellular component.

**Fig 4:**
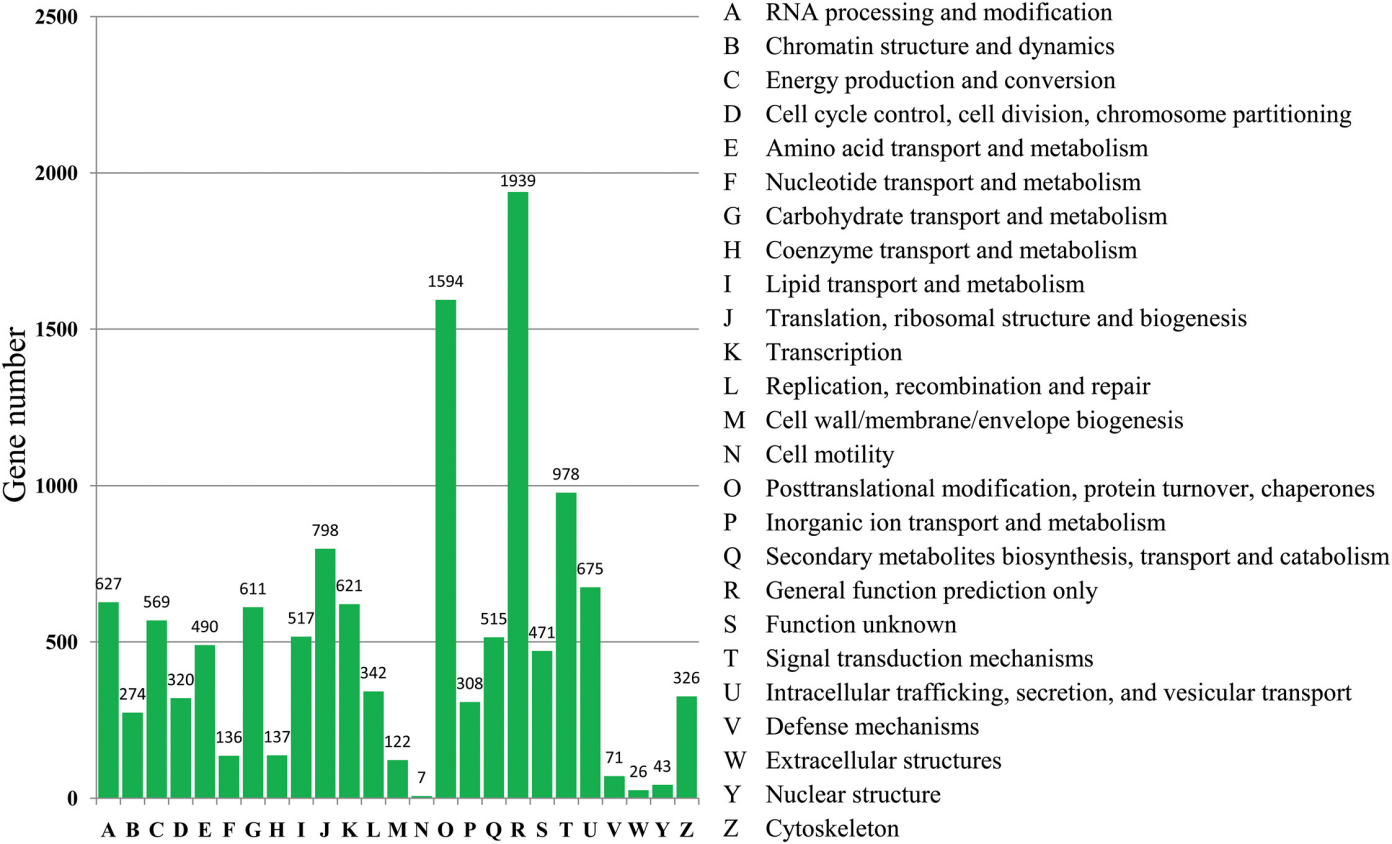
Histogram presentation of clusters of eukaryotic ortholog groups (KOG) classification.

**Fig 5:**
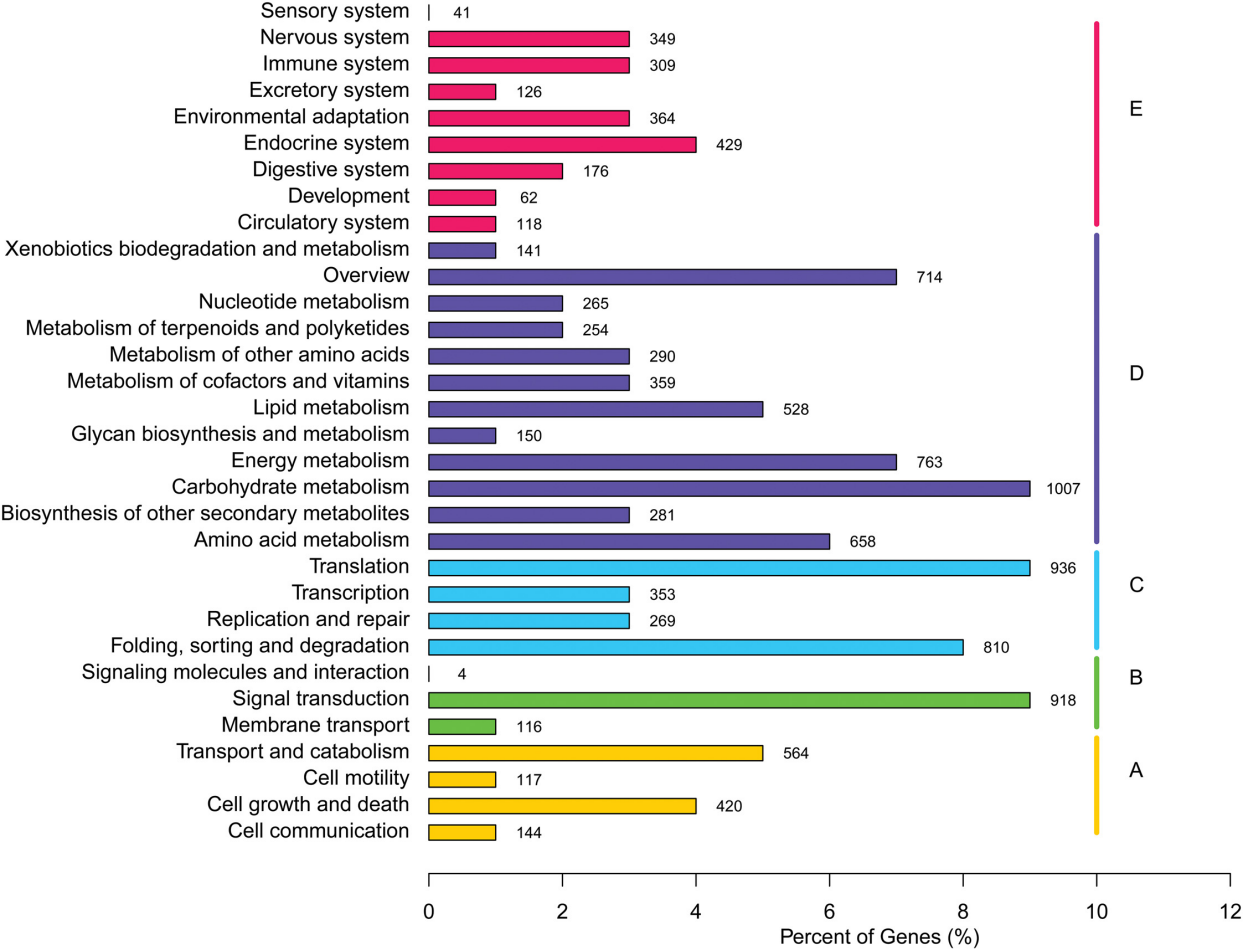
Classifications of transcripts according to the matching KEGG pathway; A, B, C, D, and E represent five main categories: cellular processes, environmental information processing, genetic information processing, metabolism, and organismal systems, respectively.

Functional classification of assembled transcripts was performed by BLAST searching against GO, KOG and KEGG databases. The results of the GO analysis showed matches to known proteins of 56 GO classes for all 24,672 transcripts, with a total of 125,547 functional terms. The majority of these GO functional terms consisted of assignments to the biological process ontology (59,019; 47.0%), followed by cellular component (37,756; 30.1%) and molecular function (28,772; 22.9%) ontologies (Fig 3). KOG functional classification resulted in the assignment of 11,175 to all 25 KOG categories. Some transcripts were assigned to several KOG categories, which led to a total of 12,517 sequences being classified into 25 KOG categories (Fig 4). In addition, on using BLASTx with an E-value threshold of 10^−5^ against the KEGG database, 10,741 transcript sequences matched a total of 12,035 functional terms in the database and were assigned to 32 KEGG categories (Fig 5).

### Identification of the CepNAC genes

The *NAC* genes of onion were discovered by searching annotated sequences among the assembled transcripts. A total of 39 *CepNAC* genes were identified, and were named *CepNAC01* through *CepNAC39* (Table 2). Among these 39 genes, 35 contained the full-length open reading frame (ORF), and the other 4 genes were partial (Table 2). The proteins encoded by these 4 genes with partial sequence contained the complete NAM domain. The DNA sequences of *CepNAC* genes and their predicted protein sequences are shown in S1 File.

**Table 2:** The information of NAC genes of onion.

Gene ^a^	Gene length	PSC^b^	PEC^b^	Predicted protein	The most similarity gene		Group
	(bp)	(bp)	(bp)	(aa)	Gene	RS^c^(%)	
*CepNAC01* *	995	1	993	331			I
*CepNAC02*	1018	30	845	272	*ANAC036*	47	III
*CepNAC03*	1349	125	1078	318	*ENAC1*	44	III
*CepNAC04*	1289	56	796	247	*OsNAC4*	80	V
*CepNAC05*	1291	135	1034	300	*ORE1*	67	II
*CepNAC06*	2081	71	1822	584	*RIM1*	61	III
*CepNAC07*	3456	802	1299	166			III
*CepNAC08*	1204	74	973	300	*OsNAC10*	56	V
*CepNAC09*	3028	44	2371	776			I
*CepNAC10*	3167	90	1097	336			I
*CepNAC11*	1122	93	989	299	*NAC1*	47	II
*CepNAC12*	2373	198	2138	647			III
*CepNAC13*	1130	103	954	284	*NAC1*	46	II
*CepNAC14*	1201	49	1026	326			V
*CepNAC15*	1689	34	1416	461			III
*CepNAC16*	1569	340	1074	245	*OsNAC52*	58	V
*CepNAC17*	2027	935	1864	310	*PtrWND6B*	51	IV
*CepNAC18* *	768	86	766	227	*RIM1*	60	III
*CepNAC19*	1417	250	978	243			
*CepNAC20*	1878	97	1338	414			I
CepNAC21*	675	2	673	224			
*CepNAC22*	1040	121	855	245			II
*CepNAC23*	1265	50	610	187	*OsNAC4*	82	V
*CepNAC24*	1095	215	931	239			II
*CepNAC25*	1163	372	1091	240			II
*CepNAC26*	2046	134	1774	547			III
*CepNAC27*	1217	245	1018	258			II
*CepNAC28*	1143	84	551	156	*ENAC1*	61	III
*CepNAC29*	1618	40	489	150			V
*CepNAC30*	1239	64	1215	384			I
*CepNAC31*	2037	451	1713	421	*FSQ6*	41	III
*CepNAC32*	2120	157	1953	599	*NTL4*	32	III
*CepNAC33*	1513	268	1143	292			
*CepNAC34*	1648	118	1407	430			I
*CepNAC35*	802	30	608	193	*XND1*	51	
*CepNAC36*	1172	298	948	217			
*CepNAC37*	2577	201	2180	660			III
*CepNAC38*	3414	296	3109	938			III
CepNAC39*	517	32	517	162	*NAM*	81	V

^a^ the asterisk indicated that the gene with partial cDNA sequence.

^b^ PSC and PEC represented the positions of starting and ending codon in cDNA sequence.

^c^ RS indicated the ratio of similarity of the protein sequence.

^#

### Phylogenetic analysis of CepNAC proteins and functionally characterized NAC proteins identified in other species

**Fig 6:**
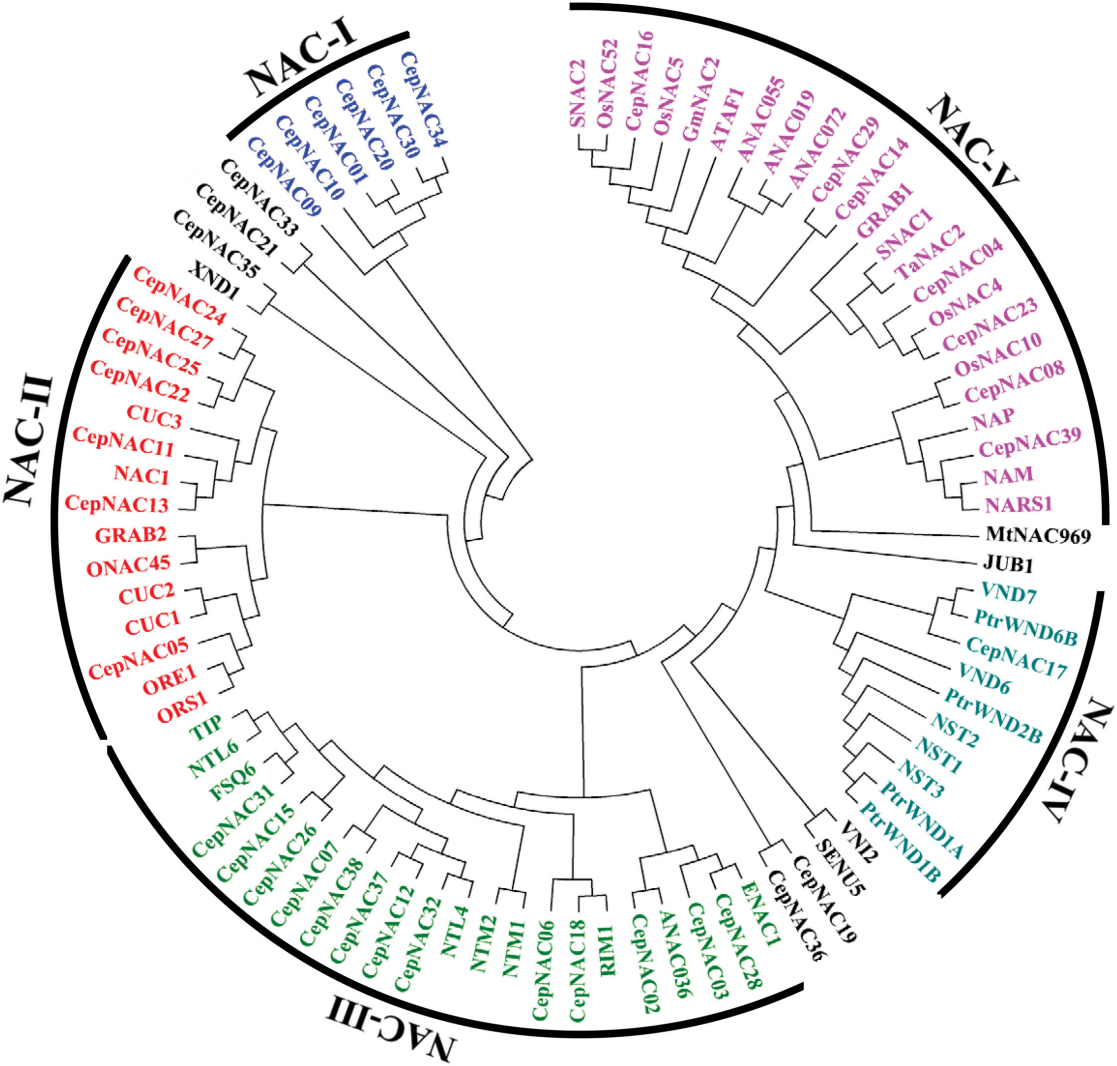
Phylogenetic tree of 39 CepNAC proteins and 47 NAC proteins published. Neighbor-joining method was used for constructing this unrooted tree by using the MEGA 4 program. Bootstrap values from 1000 replicates are indicated at each node, and are displayed in S1 Fig.

All 39 CepNAC protein sequences identified in this study, together with 47 functionally characterized NAC proteins of other species, were subjected to phylogenetic analysis. The results suggested that the 86 NAC proteins can be classified into five groups (I–V, Fig 6). A total of 6, 7, 13, 1, and 7 CepNAC members were classified into NAC-I, II, III, IV, and V, respectively (Table 2, Fig 6). Moreover, a total of 8, 9, 9, and 16 NAC proteins of known function were assigned to the NAC-II, III, IV and V subfamilies, respectively, whereas none of the NAC TFs reported fell into the NAC-I group (Fig 6). In addition, five CepNAC members and five NAC proteins of known function were not assigned to any phylogenetic group (Fig 6). Interestingly, all 9 NAC TFs reported to be in the NAC-IV subfamily function as secondary wall synthesis regulators, and all 16 NAC TFs reported to be in the NAC-V group function as stress tolerance modulators (Fig 6; S1 Table). This suggests that the NAC-IV and V subfamilies might be functionally related to the processes of secondary wall synthesis and stress response, respectively. On the other hand, the function of NAC TFs reported in the NAC-II and III groups was very diverse (S1 Table). Seventeen CepNAC TFs showed highest homology to the functionally characterized NAC proteins reported in other species, with a sequence similarity ratio ranging from 32% to 82% (Table 2). For 7 of those CepNAC proteins, the similarity ratio with the NAC proteins of known function was more than 60%.

## Discussion

### Characterization of the transcriptome of onion leaves

Because of its cost-efficiency, high throughput and speed, NGS technology offers several advantages over traditional Sanger sequencing, which is costly, time-consuming, and laborious. NGS has been proven a powerful tool in several researching areas, including re-sequencing, micro-RNA expression profiling, DNA methylation, and especially *de novo* transcriptome sequencing for non-model organisms [27]. Based on this technology, the transcriptomes of hundreds of species, including non-model organisms, were characterized [22], and a large number of expressed genes have been discovered, thereby accelerating our understanding of the complexity of expression, regulation and networks of gene in model and non-model organisms.

Onion is an economically important crop. By Sanger sequencing for the cDNA library, a total of 11,008 unique ESTs obtained from this crop [28], which first provided an insight into onion expressed genes. In a recent study, Rajkumar et al. *de novo* assembled and characterized the red bulb onion transcriptome, generating a total of 293,475 genes with a total length of 281 Mb [16]. Obviously, the number of genes assembled in the above study is enormous, far greater than that of other *Allium* species, such as garlic (135,360 transcripts), Chinese chive (60,031 transcripts), and Welsh onion (53,837 transcripts) [29–31]. The transcriptome characterized in this previous study was also huge (almost two times the genome size of *Arabidopsis*). Probably, there are some redundant genes in the previous onion transcriptome. In addition, as only the onion bulb was used for transcriptome analysis [16], genes that are specifically expressed in other tissues such as leaf were not identified.

In the present study, we have described the transcriptome analysis in yellow bulb onion leaves, and identified 117,189 non-redundant transcripts. However, among these transcripts, there were only 33.7% of them achieved for functional annotation. This low ratio might be caused by the large size of onion transcriptome. Our result revealed that onion had a transcriptome of about 74 MB. Because of its large size, it is difficult to assemble for the onion transcriptome. Therefore, although that the average sequencing depth of coverage in the transcriptome was more than 170 folds in this study, many small sequence fragments still were generated, and they were difficult for annotating the function. Actually, a similar low ratio for the annotated transcripts had been observed in onion transcriptome in previous study [16]. There were substantial amount of transcripts obtained in this study, which will immensely help us to explore the major genes and accelerate our understanding in the onion growth and development.

### Identification of 39 CepNAC TFs

There are 8,133 NAC genes that have been identified from 74 species in the PlantTFDB database [32]. Previous studies revealed that NAC TFs are involved in many aspects of plant growth and development, including modulation of senescence, secondary wall synthesis, stress tolerance, and embryogenesis [11, 14, 15]. However, no study to date has identified NAC TFs family members in onion. In the present study, a total of 39 CepNAC TFs were identified for the first time. Sequence analysis revealed marked homology of 17 CepNAC TFs to NAC proteins of known function reported in other species. Among those, 7 showed more than 60% sequence similarity with the NAC proteins previously reported. The high homology in protein sequence will provide further insights into the molecular mechanisms in which CepNAC TFs are involved.

Phylogenetic analysis was performed for 39 CepNAC TFs and 47 functionally characterized NAC proteins reported in previous studies. Among these 47 NAC proteins of known function, 10 were found to be involved in secondary wall synthesis. The results of the phylogenetic analysis revealed that 9 out of 10 NAC genes involved in secondary wall synthesis and 1 CepNAC TF (CepNAC17) were assigned to the NAC-IV subfamily, suggesting that CepNAC17 TFs share a conserved sequence with NAC TFs responsible for secondary wall synthesis. Moreover, CepNAC35 showed 51% homology with XND1, a regulator of secondary wall synthesis in *Arabidopsis* [33]. Most likely, CepNAC17 and CepNAC35 have a potential role in secondary wall synthesis in onion. In addition, 16 NAC proteins of known function fell into the NAC-V subfamily. Interestingly, all these 16 NAC TFs were found to be involved in the regulation of stress tolerance (S1 Table), suggesting that this NAC group might be a stress-responsive NAC subfamily. In light of our results, this stress-responsive NAC subfamily would contain 7 CepNAC members.

In conclusion, 39 CepNAC genes were identified for the first time in this study. Analysis of sequence conservation revealed that 2 CepNAC TFs had high homology with NAC TFs of other species that functioned as secondary wall synthesis regulators, and phylogenetic analysis found that 7 CepNAC TFs were contained in the stress-responsive NAC subfamily. The identification of the CepNAC TFs reported in this study will provide a basis for further characterization and validation of their function in the future.

## Supporting Information

S1 Fig Rectangular phylogenetic tree of NAC proteins with bootstrap values. (TIF)

S1 File cDNA sequences of 39 *CepNAC* identified genes and their predicted protein products. (DOCX)

S1 Table Details of the 47 functionally characterized NAC genes previously reported in other species. (DOC)
