# Supplementary figures and images for: Identification of an NAC Transcription Factor Family by Deep Transcriptome Sequencing in Onion (Allium cepa L.)
Source: PLoS One. 2016 Jun 22;11(6):e0157871. doi: 10.1371/journal.pone.0157871 (PMC4917099; doi:10.1371/journal.pone.0157871)

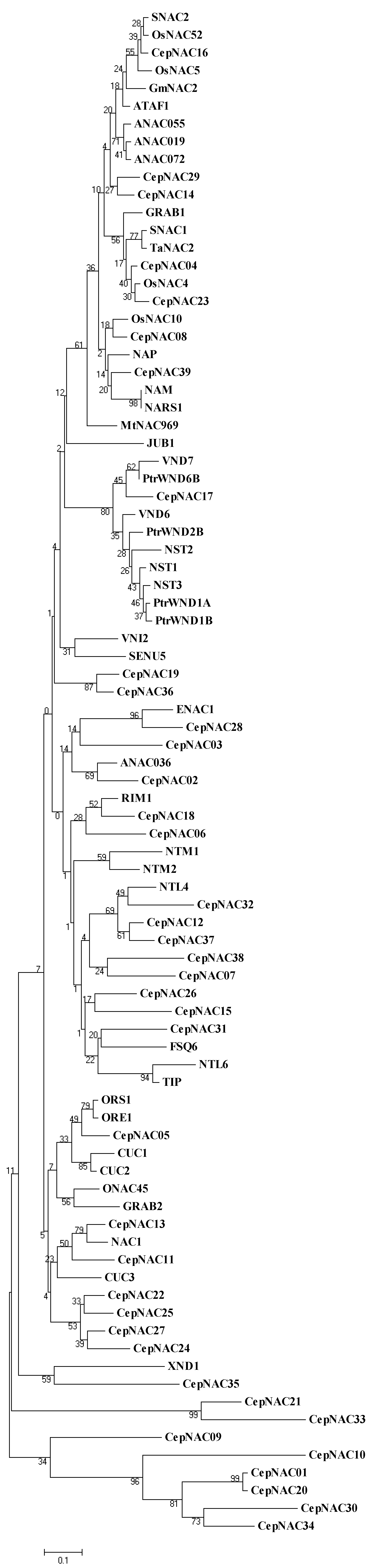

Supplement: S1 Fig — (TIF) [file pone.0157871.s001.TIF]
