# Supplementary material for: Identification of an NAC Transcription Factor Family by Deep Transcriptome Sequencing in Onion (Allium cepa L.)
Source: PLoS One. 2016 Jun 22;11(6):e0157871. doi: 10.1371/journal.pone.0157871 (PMC4917099; doi:10.1371/journal.pone.0157871)
Supplement: S1 File — (DOCX) [file pone.0157871.s002.docx]

>CepNAC01

DWFGLPAGVKFDPTDQELVEHLEAKVYEQDSNLSHPLIDEFIPTIEGEDGICYTHPEKLP

GVTRNGHYKHFFHRPSKAYTTGTRKRRKIQNESDHGQIQRGETRWHKTGKTRPVIVNGKQ

KGCKKILVLYTNFGKHRKPEKTNWVMHQYHLGELEEEKEGELVVSKIFYQTQPRQCNWSS

HDKSGSSSSKEIVGQSSQLDHQMDNYQFAPYSKSFDQIGMTTAEATTNTRNVHVHNLRHD

QNQIIAATSTFHINQPTHPISAVISHHHRSGTLINHDDLYHMPRIVRQSTNTFQQQQQQH

TNMEERSDAGLEELIMGCNSSGNRQAQTINP

>CepNAC02

MERYESKLDLPGFRFHPTEEELLDFYLKHKVNNRNHSFNIIGTINLYSYDP

CELPGLAKIGEREWYFFVPRDRRQSSGGRPNRTTERGFWKATGSDRPVRSAADPKRVIGL

KKTLVYYMGRAPRGSKTDWVMNEYRLPGSSNSSVPPPHEDIVLCKIYKKATPLKELEHRA

AIAEARSRINNNMLPAENASSSSQIDYKFDTENSSNEVDDIELLMNQDGASRNEIEIIAP

RSSAVVQPVDVPELQIPNNGFDYWMQDVTLWSPYANLLNF*

>CepNAC03

MRDITIVEASIMSSNSRDDHEHDLVMPGFRFHPTEEELIEFYLRRKVEGKRFNVELITF

LDLYRYDPWELPALAAIGEKEWFFYVPRDRKYRNGDRPNRVTTSGYWKATGADRMIRGED

SRPIGLKKTLVFYSGKAPKGIRSSWIMNEYRLPHSDATDKFQKAEISLCRVYKRAGVDDH

LHPLPNSRASSSVNTELPQTLAPIVGDSSSSSQSSMDKKTATNFFNTTTTTNSVEEVGTT

LLTQPKNTNGTLTSLLAPNTFANYIDHNNVVVSTSAIDQLVNPLPLMQSQLLPFGISDKL

WDWNLLPESGRDHYSNFK*

>CepNAC04

MEQLNLPPGFRFHPTDEELVVHYLCKRSAHQRLPVPNLIAELNLY

KHDPWDLPHRALFGEKEWYFFTPRDRKYPNGSRPNRAAGRGYWKATGADKPIVPKGFTKP

VGIKKALVFYSGKAPRGVKTDWIMHEYRLADSVTNSKKGSLRLDDWVLCRLYNKKNNWEE

SKPSVGSDDSYVSDVVDEDMERILGQQSCREMEGCEVKMKKEETDWFMDFSFDELQNSFG

GFGSSGAGTGFGSLCPDYLLQ*

>CepNAC05

MDESINLPPGFRFHPTDEEIITHYLSPKILNQNFTTIPIGEIDLNKCEPWDLPS

KARMGEKEWYFFCQRDRKYPTGMRTNRATEAGYWKATGKDKEIFKGKNNTKVLIGMKKTL

VFYTGRAPKGVKSNWVMHEFRLEGTSAEANFPKPAKDEWVVSRVFHKSNGAKKSPISDYF

GSGSLPTLMDPTPFVNNEDLINNFDMMNSSCFYNVPGLDDAQNFNQNYQQMGQVNGSTSN

YNYLHHNPMMKMEQFPKRSMVSDDTGLSTDRNTEISSVVSGHEIKNEFEMPTVGSVLDLE

NMWKF*

>CepNAC06

MGAAPDMSGAQLIPQEEIEVDNSISDCRNWPPGFRFHPTDEELV

LYYLKRKICHQKIKLNMIGDVDVYKWHPDELPGKSLLRSGDKQWFFFSPRDRKYPNSSRS

NRATANGYWKATGKDRTISQNSKPVGNKKTLVYYHGRAPKGQRTDWVMYEYSLAEQVLIN

LKNVQDYFVLYKLFRKSGPGPKNGEQYGAPFREEEWDEDVVDDNHMNQTNSNFQMIENPP

NNSVDNELPNGDMNANQNHDLDRMLSQVATELDVVPLNSELHSVNNGSGNQVDNNARNER

ALNLEQNSLPSYDLEDILLQLSNEQDVMHQLPDSTSYAPNVLQGNGARDDDLDINNFMTP

SHYINEVPVQQTNAEEEYLEIEDLINSDCQAQNPDNMNYNSELNHIGDQFVFPDEYFDTQ

MFLEDAMGSDSGMNQQNTYWNDFLNNEQQMPASNITTDFPRLEQHFSSSTTSVELCTNVT

ASSSGVMGANNNNTTESWFDSNLSAFLGSIPSSPAFASENTLLTKALNRVSSFRGGRNKE

MRDPYAAVNNNNGIIVRRGRQNGGFLFTSFLVGLGAVVWFLSIGITVQIFKGFWNRYFS*

>CepNAC07

MEALNLLSLPPGFGFHPSDAELITHYLKNKILGNFHQNEIIPLVDIYKH

EPWDLPARCCVPTRQAKWHFFSHGDRKHPKGTRKNRATRAGFWKSTGKDRVIKHQNRNVG

TKKTLVFHEGRPPVGKRTAWIMHEFYVNDKEFMFTPEMKVPFYHYAHHFYYDSNFF*

>CepNAC08

MTNQTPDLPPGFRFHPTDEELILYYLRCQASSSSCPVPIIAEVDIYKFDPWNLPDKAM

FGDREWYFFSPRDRKYPNGVRPNRAAASGYWKATGTDKPITTGSENENIGVKKALVFYKG

KPPKGVKTNWIMHEYRLCDNKNKNSYRPMKLKDSSMRLDDWVLCRIYKRNGHDQDDVHEE

SDVSNTAYMAQGYEDDPMKMHKSFSVSELLNETDIADISQLLDAPTTNTGIDNQNLYGMN

QRSLKRQRPEEENYLQGQKLTNINSWTAGNFSSQLDFDSQAYINQQLMFNSQLDMQQKWL

S*

>CepNAC09

MMKIPTSEQNLCSTLPPGYRFHPTDGELITYYLHRKISNIPFPVDLVPQINLYGYE

PWQLPALSKFTVTGKEWYFFNLVSGHKKGSFVRPSRSRTNVNRETPEGQWISGLKREILV

DGKIVGWKRKFTFFARTNGDRKKTDWYMMEYRSSKDDDSYPKGASLVISKIYAKVTPAQL

QIEPTSAHKVVANEGNSSVQDIELDTTQDRKSSVDGEQDLYSTKLEQVSKMAMPKAGQSI

LNDETSSSKTFTPSGYLSLPGFWFYPTDNELIEYYLKHKVLNLPIHDNAISEIDLFKWAP

WDLPSQSRLQHKNMQWFFFNLHDRTHIHSYYNVNNQGVEGYWSSGTDLPVHNTSTNAIIG

MKNFFVFHQGKFPNGKKTYWVMHEYRLREDGQIGPICVVIKIWNVSIQFKLTASETHEKE

VLAPVDKSTCALVSNLEAAKGDNVLHSPNNSEQGSSTPFMVGSVAEVAYTHENVPIAWYT

ATVTEIINEKVLLVEYLSLKNEDGSLLRETVSIQNIRPCPPSTQQVRDFQLLDIVEAFYN

GGWWQGVIAAVKDQSKYNVKLMLLEDEIISSNTELRVVYDWINEEWILASQDEPLESQNN

VESLHACSPLNKRKDPDPVATSMSDVDISGLLPSFKKNKNDQESRRPILRSKKGRKSETV

EDCINFSASRCVETSNVLKLGLEVPTFIHEEKQSNIDTNTAVEKMSEDSQQIGEVPKTDQ

DAVQDKSLESRNYVELLLACLLLYAKTQGSRFKYYPDKSRSIFFVSHHHLHKMETIMNQ*

>CepNAC10

MAEIGVKGDTGWATNLAKKWIKLPRGVKFEPSDEDIIWHLLAEIGEGDAKPHPF

IGLFIKFWNIDGPHKIPPGVKKDGSMSYFSHKPFTPQEIRSRKHNEGMKDGCVNGFWRKL

GNTEHVFMDGTQYGSKEIFVLCKYLKRHSRFKKTNWVMHQYHIGTGNVKGNLVISKVFYQ

LNSEQGEKTVRNFGISTETVLSLATNGRQVHAYDSCMKSLAVTNQNDTMGNHHNEANASQ

GDVSRAMVEPSNCIQNNPMEVAPSIPAAVSESGNGEVDDLTLQERYRQLFSNIDKVEPEA

LQGQCKSMLCNMNNDTGHNLISELSEDAGSGVHVEENEVQM*

>CepNAC11

MSYLSMMEATMPPGFRFHPRDEELICDYLAKKIDGDNTDRSLYDGCPTLMSVDLNKC

EPWDLPEIACVGGKEYYFYSLRDRKYATGQRTNRATISGYWKATGKDRPVFRKGVLVGMR

KTLVFYQGRAPKGKKTDWVMHEFRMESPAGHTLKLPVTKEDWVLCRVFYKSRGLTSKPIT

MMNTTNYEDTIASPSLPQLTETYITFNAYDQVPCFSNNQFQNNPMAMIGRSMAIKNDAAA

QMDGFDQLSLNQVGCDRNGIVDVLNQLTKMEEKTKREAHTDSYGGGNIDSYFSENGGSNM

W*

>CepNAC12

MIPCESSSSSTRLAPGFRFHPTDEELVSYYLKRKISGLPLRVDAIAEIDLYKKEPWEL

PSLSKLQTRDMEWYFFSPLDRKDSNKLRTSRGTMEGYWKTTGKDRAVRSTYSNVVVGMKK

TLVFHEGRAPKGKRTNWVMHEYRLESSENDAASSCENTALVHPQDSFVVCRIFQKHGSGP

QNGAQYGAPFIAEEWEDIHDNKTPECAERHGNGHDAALMCLTNEQYYAELDIDMNQILAN

HQDNNESTNSKDCQSHADEPTQYQLVDNPVADHLSGEQICDSHAYDISFTLEDLIDFDKP

NNIDGNLQENVVCIDGNTARNSSIASDKSDGYLELNDVYANPNGHCTNNTLDDCMRSNHA

NNANTSLKIEDAEIFNNSNVTTATEGVHSVQPYDYLNPADGNYLEDDTMFYDAPSNDVQF

TEDTYMNIKEYNPTVTDFDLVDDLMAFFDATEQNVDPLSADQSDIDNPTVKSEVEGSNYP

TGKDCLQTPGANMGMGAPSTEFIDAANPLEKTNVEANDGWNKSFRKHVVDMLGSISAPPA

MAAADSCKGKGIQISSSGSSSSIRVSAGMIQIRGISVTGHPEKWTLSKDGEINFIYTYAN

GAVCKLASRQPPPATVFGALLRSGIFLFGFSVLVLSITMKLGVGVYRK*

>CepNAC13

MSYLSMMESTLPPGFRFHPRDEELVCDYLAKKINGSDGTSHGGLYDECPALICV

DLNKCEPWDLPGDACVGGKEWYFYSQRDRKYATGQRTNRATLTGYWKATGKDKHITQNGV

LVGMRKTLVFYQGRAPKGTKTDWVMHEFRMESFDHASKFPQPKEDWVLCRVFYKDRGMAP

TPTYSMTNAMNYDDSMLVERYITFDGYDQVPCFSSSSLQNPTAAVERSAPAYREQGGMEC

FEQLNLNQVDDNSAIASVLNQLNRVDGKTRRDVCIGGLGEGNLGLMMLS*

>CepNAC14

MTNQAPLLPPGFRFHPTDEELILFYLKNRAEATPCPVSIIAEVDIYKFDPWNLPDKAMFG

DKEWYFFTPRDRKYPNGVRPNRAAGSGYWKATGTDKPIVTTIGNENIGVKKALVFYIGKP

PKGVKTNWIMHEYRLSESNIKSSNNIARSTRIRNSSMREDSKSNVKQFLMLYDHRDSTNI

RSFVYMXLDDWVLCRIYQKGGHLKEDSVENESSFEDNKLEIQRSFSLSELLNEADFSLLS

RFIETPVDTGMSGANMNVTTYAKAEPSVFVNGLKRQRAEEGENSMYPLKKNIGNSSWDYM

NFGDQYDSQTFYDQQLLMNSQFGMQ

>CepNAC15

MEEIPYDKLPLGFRFRPTDEQLINHYLKRKINGTIHPSESWVIPEIDVCRTEP

WDLPSKWRVKSSDLEWFFFAAVDRKYPSRNRSNRATENGYWKATGKDRLIKSKKVLVGMK

KTLVFHIGRAPRGTGSCWIMHEYRVVNPDGTDVVGDEGAFVLCRLFKKDKDILNVTPLSQ

ALPNSDLSDDPHTVNPENSYGQITEYNVANNEVLPYLVPTTIKPEQSHSNSDITADVGTH

QELKIKQEEDSLFDGLKDFLGSEYDQILSPEAPENYTINPKMFEQQLCVLSTSDMAGAIS

RDSDDLKPMWRLHNKMVYKGASKRSQGVPSEMHEPIEWETHFTCDDESLKASSSYETEIN

IRHRMPESSNSSMNYPSPQGTAERRIKLERVRRRKPKKQIEIEPPMATSVVSDQSSFSSN

SDGDIDTNDLKVKSPTSGYSVPMHRIAVAVLVIVMMLCAGTMWLLTS*

>CepNAC16

SLSSGLASYGEKEWYFFSPRDRKYPNGSRPNRAAGSGYWKATGADKPVGTPKPVAIKKAL

VFYSGKAPKGDKTNWIMHEYRLANVDRSARKKNNLRLDDWVLCRIYKKKGAEKPNGNEQR

AAYDSRMIQQVPEFEQKPEVIQHVTPTFSHVQQNNGYGKNDWFNFDASESMPRLHADSSC

SEHVLSSDFTCDREVQSQLKWGELERALAVPFNNVNATSSFPTLDNGPLTDPLQDIFMYL

QKPF*

>CepNAC17

MECCVPPGFRFHPTEEELVGYYLTRKVASQKIDLGVINDIDLYRIEPWDLQ

ERCKLGYEQQSEWYFFSHKDKKYPSGTRTNRATNAGFWKATGRDKEVLSKSRVIGLRKTL

VFYEGRAPNGRKSDWIMHEYRLQSEHGPSQEEGWVVCRAFKKPIPNPRPSYYNSYSPYSV

REQNQYEQLNDLITRPTQNIIESNQVPNHIFDQPLGSMVVSKLNEPKVITSRGIFEYIPQ

VDSPSISTTNIAARDQCCKRDDSKGQFVDWKALDNLLASQLNESTSCSNSKFPMVSLLDQ

YDMNDPSHEDEFLASFDG*

>CepNAC18

MATMSLPPGFRFHPTDEELVAYYLKRKINRRKIELEVIQEVDLYKCEPWELPEKSFLPSK

DLEWFFFSPRDRKYPNGSRTNRATQAGYWKATGKDRKVYSQKISIGTKKTLVYYRGRAPH

GSRTDWIMHEYQLDESECETPSSGLQDVYALCRVCKRNAPGPVIIAEKSNSYSLQYESNS

SRIIQKPMNCVEGKWMQFLDEDAFDANVSPFQDSSSFPYVPSEVDVL

>CepNAC19

MEKQNPSSIPSIVRHNNHNGVVKLPPGFRFHPTDEE

LVLQYLRRKLFSYPLPASIIPEIDLARHNPWDLPGGCEQERYFFALREAKYANGNRTNRA

TECGYWKATGKDRVVVATKCGRAVGLKKTLVFYHGKAPIGQKTDWIMHEYRLNISNPRSC

GLSHMKNSTLSDAIEVKDWVLCRIFKKKRSAKASVEQEEHRFIDFMGRGMNGRRSSLPAH

SDSSCVTDHASDESKYGEEITSSSYP*

>CepNAC20

MRKNYMDSISSSDLIETKLEEHRMSGSKQCPSCGHKLEFNKPDWVGLPAGVKFDP

TDQELIEHLEAKVSEEEMRVSHPLIDEFIPTIEGEDGICYTHPEKLPGVTRDGLSKHFFH

RPSKAYTTGTRKRRKIQTECDLQSGETRWHKTGKTRPVLVNGKQKGCKKILVLYTNFGKH

RKPEKTNWVMHQYHLGELEEEKEGELVVSKIFYQTQPRQSSLPTEKSTGSCSTSSKEVNI

TQSDRHGYNCNLEQLQHMKNIDSNFSFAAFRKPFDEVGLREVPTQARDQATVASIACDKE

HEIRVTSAYHISRPTNPMPTTIQNPLHQGSVVLDHHDPYNLSTMLLHQTNKFQQHQQQQP

QQQPPPPKLVERSSAGLEELVKGCTSAGNRGEPSIPVNTQETDWLKYSSYWTDNPDHH*

>CepNAC21

DPFTIDPQLSSDNHWYMYKSVVSDIYGKESVIKGKNGYWEFMNDINIPTSTAIGMKITRE

FYLGNVTFGDKTAFRMHEYHVKLEKNRFKENSLSRIFLEIEVKKEEKSNIDGENIESKLP

CLVNQQEKNSSLKQCQNDLEVSEENEGQPILPSGLMNEEAGFEGFIEFNDFGAEDGSSSF

SVNTSSSVKSMTSDEHFESEIVGFDPEAVLSDIANDVNDVNASV

>CepNAC22

MGLRDIESTLPPGFRFSPSDEELVCHYLLKKVTN

VRVYEGTMVEVDLHTCEPWELPEVAKLGDNEWYFFSFRDRKYATGSRANRATKSGYWKAT

GKDRPIYNQKTHGVMGMRKTLVFYHGRAPNGIKTAWVMHEFRMENPHMPPKEDWVLCRVC

QRRREDIASSSLQMTSSSQTPRPVDRSMLHGCPEMMWDPHQDGQSTNTNFDSNNLEALQY

NIFDFTQDMDMSMGSKDIDDDYGPFLDLIS*

>CepNAC23

MGRRTRDAEAELNLPPGFRFHPTDEELVVHYLCKKVTFQRLPVP

IIAEVDLYKFDPWELPEKALFGQKEWYFFTPRDRKYPNGSRPNRAAGRGYWKATGADKPV

SPSGSKKPLGIKKALVFYSGKAPRGIKTDWIMHEYRLADANRTPANKKGGSLRVRIWIRP

VLFRNCVKLLNSLNCILFSSVG*

>CepNAC24

MGLREIESHLPPGFRFHPSDEELICHYLFKKVNNERNDEGAMVEVDLHTSEPW

ELPEVAKVSENEWYFFSFLDRKYATGMRANRATKSGYWKATGKDRMVYDPVTHTLIGMRK

TLVFYRGRAPNGIKTGFVMHEFRLGNSHMPPKENWVLCRVCHKRKGEVEMNNESFNSQPF

MGKEIENSMFANGAQSSSSFPDMNNLMQCNFLDFTPEYAQDYDLPFDVGTEENAMHYENL

VDFSY*

>CepNAC25

MGLRDVESGLPPGFRFFPSDEELVCHYLHKKVARLAMKEETMVEIDLHTCEPWELP

EVAKLSVNEWYFFSFRDRKYATGSRTNRATKSGYWKATGKDRTIYNPSARNEIIGMRKTL

VFYSGRAPNGIKSSWVMHEFRLENSHSLPKEDWVLCRVFNKRKEEEGSAITSSSSSPLFS

RDCPMEGRMYKHIEPLLSGDHNNSYLCPTYESIGDFNDMSNLMSFNVPQAEIEDFGFLMD

VGL*

>CepNAC26

MTVVPIKSLPLGFRFHPTDEELVNHYLKRKINGRIRSEMEVIPEIDVCKCEPWDLPGR

ALIESDDPEWFFFSPKDRKYPNGHRSNRATEAGYWKATGKDRYIRSKAPSAIIGMKKTLV

FHRGRAPKGVRTNWIMHEYRTTEPEFDSGEQGGYVLYRLFRKPEESAPLYNVDEMDRSDL

SITPSKSSPSEIQNEVDVFEELQTPAMLQWNQVSPVSDIQENTKFQVAVNKYDNNNSLNL

KDGYNNNLEQRELVDPLLEALGQVDSNGIPNIPSPLCTYVDNSFLCNGNQESNTGLFQAN

FNEPDPMDDLLNSFFSNEVDSPSGHEGVGYSDPEWFYMDASNDYSERTFPENAFLQFQSA

HDDYSLDSGSESLHDLFNNMDESNAQFNLSNHENNLEESGSTGIKIRSRQPRSVIPNNMP

AQQGFAIRRIRLQVSTDDAERKLKLEDNEEICETEQVENNGEDEDHGDINVAPLSLKESE

PNLRLRAKTSVEINLNEECKYSVKEKEEIKRPVTFNYVIFMTVMIMIVVLLLVGMLMWKE

EILGFVDF*

>CepNAC27

MGLRDIESTLPPGFRFYPSDEELVCHYLYKK

VANVKVYEGTLVEIDLHNLEPWELPESSKLSDNEWYFFSFRDRKYATGSRANRATKSGYW

KATGKDRAVYNLKTRRLVGMRKTLVFYSGRAPNGLKTGWVMHEFRIENPHTPPKEDWVLC

RVFQQRKGEAGSSQSQSGHSSPAMADGQSVKDECFQTMWDPHQEGVNTTDNLEVLHYNIF

DFTPELSTTYTAGMDMSMGTMDVNDYELLLDGNLLSNMGELKDLIW*

>CepNAC28

MNNSEQQIPPGYRFFPTEEELISFYLHNKLANAREEEIEQVIPTVNL

YYLDPWQLLPFASVQESEQWFFFCPQQEREAHGGRPARTTPSGYWKATGSPGSVYSGVNK

LIGVKKSMVFYEGKAPRGTKTKWKMNEYRALDETVVNGDSPKVLVSTF*

>CepNAC29

MSGGDLKLPPGFRFHPTDEELVKHYLCRRCTGMNIAVPIVADIDLYK

FNPWELPEKALYGEKEWYFFTPRDRKYPNGSRPNRAAGSGYWKATGADKAIGAPKPIGIK

KALVFYAGKAPKGEKTNWIMHEYRLADVNRSVKKLNSLRVCF*

>CepNAC30

MARACWLIDGRGIARKVRDASSFSKCQIKELGTEAHRECPNCSHIIDNSDVSLEWP

GFPAGVKFDPTDIQILEHLAGKVGLPNSKSHILIDEFIPTLTEEQGICYAHPEDLPGVKN

DGSSVHFFYRISNAYATGQRKRRKIGNKECSSASEEKARWHKTGKTKPVIDKNGNQIGWK

KIMVLHQSLKKGSKRKSSADKANWVMHQYHLGTEVEEKNGEMVVAKIFFQQNSKQNNKVD

AELSREEPINVASGPRTPKMVTPLQPRKAKQSPMSEAEYREEVIPQSVVNKNDEGNFLLL

AGESQAVDEFNPKPLDELLFCHEVFDNSAYTNDTSFGNNELLNTDDFNSSNPFDDLNNIP

LGTPPDFNLSDIQFGSQDSISSWLNRM*

>CepNAC31

MVDKLTLPGFRFHPTDAELVLYYLKRKIQGRLSKSQAVISEVDIYKFAPW

ELPEKSCLQSKDLEWYFFCPRNNKYSHGNRTNRAATGIGYWKPTGQDRPVINKKQTVGKR

RTLVFHTGKSPDGTRTDWVMHEFRIENSDELVNAGFKQDAYVLCKVFEKSGQGPRPGEQY

GAPFNEEDWDDEVDTVISNDNSLPSALVIDSELLQSIASQAGELNSVHHVQNSSLIQPSS

VGAETSLQVHFEVGESSRAQELSSMQCNLPPSFSDGVSLGSLTQENAHLVSEMPGSALPL

ENSIQYADLDFDLGDLGDLDTSIHHLMEPDDGFSVLEVLSYVDNQELQHSAEYANGDYLT

VADIASENQANVMEQPPLTNVQDSLNVTSGDDCYDHIVNSLLTDNPQHTEMSDVFLPCIA

YPDCQCCNNL*

>CepNAC32

MTIQSSSLSSTLAPGFRFHPTDDELVSYYLKRKKSNSPLLVDAISYIDLYKYEPWDLPSL

SKLKTRDMEYYFFTTLDRKHDSRIRTSRRTLDGYWKTTGKDREVYGVGKVVVGMKKTLVY

HSGRAPTGARSNWVMHEYRLNSDSSSTADDLQXELDETCGVWFENLDDMFYAVGRDISFK

WGKETLVLCRIFQKNGSGPQNGAQYGAPFREEEWAEGQGLGPKTNEGELNVLMHANNIEE

NQALADGQGSTDITANSEENVGSQPRNPSNLLELDEGINVPEPQVADSNIKKMYTFEEQV

ENHSGQKDGYLELKDFHNDDSFGQMGENYRDESTAVPQVDYASLYLDNAAENMNSNGLRT

IDPSPISYDYHSAQSVAVDENIFFDAFSNNFSFTEDILDDMNNASGYDMVNDLFAYFDAT

ENNLQNDMEIFNSTTTVQVNVKSEVDSGHVKEKGSSHSASSSENEALNKGNSATAEVQSR

MGWKSAVKKQVVNMLGSISAPPAMAEECSTAIKAIPAPTSSIPATSGIIKIQNILLDESM

DFWSTKKMIERSEFELSSYEPVVKRNGAFSTVLRCGFYLFGASXADLFCMFENCNVFA

>CepNAC33

MCPIPPLLSADSSDEFVVMFLDSRSVDDPMPSNVITDVDPFTIDPRLSSG

DNWFMHKSIILDTVDERSMIKGNSGCWEFINNISISMPTSTRTIGRKITRKFLLGNGTSG

DKTTFGMHEYLVEQDKNKFKETRLSRIFSQMDEVKIEEESGTGGENTESKLPFLVNQQER

NSSLKQPENEFQASNGIEGQLELPNYVMNEEAGHEDFIELKDLFGPEDGNSSFSGNTSVK

SMTSDENFDSEIMGFDPEAMIRDIANDIYNANASVSSDPAVIRPSISLGIFTRNLFHFVV

A*

>CepNAC34; len=430 start:118 end:1407; minus strand

MTGPSWLIDGKRIATKIKYASESKDPNRVNWISNPTKPCPSCNHIIDNDDVVQDWPGLPE

GVKFDPSDQELITHLIAKLEKGDERPHPFINEFIPTVDEDDGICYTHPKKLPGVKLDGSV

SHFFHRTFKAYNSGTRKRRKIQHNGADDVRWHKTGKTKPVIVDGKHVGCKKIMVLYSSLR

NGEKPQKTNWVMHQYHLGTGEDETDGEFVVSKLFYQQQPMLKSCSNANKFEVVEEASPVM

ETMDNDALPVPPSLDISHAFSVHDKSAMVAVCSPLDNDQNCVQESVTTDSGTIINDSTSD

KILKQGNIADQDNENGTEATEDPKWWEGESQFLLDSQQLAEGIALCDEFLHSQSQTSCGG

EIEEATKRIKPCALSEYAKKGGVDVFKKDLEECQILNGDHFATDDSQPQLPNFELMDTPP

DFRLSQLEF

>CepNAC35

MGDRNPNDIPPGFRFYPSDEDLVVHFLARKASHLPCQPNIIPTLDLHNFDP

WSLYGNALQGGNQVHYFFARNTQHRATPSGYWSPITVDESIMNSDQVIVGFKRTLMFYFG

DMKTNWVMHEYHLSDAIVNNTRRSSKKRGTSRIEINKWVICGVYESSCNSHSNHNDGGRE

LSCLDEVFLSFDDLDEVSLPN*

>CepNAC36

MEKQNPSPTPCISRHSNTIIINGVMKVPPGFRFKPTDEELVLQYLRRKA

FSYPLHSSFIPEIDLSKHNPWDLPDGGCEEERYFFVLRETKIATESGYWKATGKDRVVVA

TKCGRAVGLKKTLVFYKGKAPRGQKTDWVMHEYSLAISNIFSQRKSLSLHGAVGIKDWVL

CRIFMKKRSGISYVRGLPSPAHSDSSCVTNHAVGKSNDGEEISSLLQ*

>CepNAC37

MFSESSSNSTKLAPGFRFHPTDEELVSYYLKRKISG

LPLRVDAIAEIDLYKKEPWELPFFSKLQSRDVEWYFFSPLDRKYSNRTRTNRATVEGYWK

TTGKDRVVKHAYSNSVVGMKKTLVYHHGRAPKGKRTNWVMHEYRLECSEDDANGRNSTTL

VRPQDSFVVVRIFQKNGSGPQNGAQYGAPFIPEEWEELQGNIAPKEEEENARHTVLVHQT

NVQHSDKAYVEQKNPLLVDPQQNTDHLSAESKDGQSQPDDQTQIHVAGNHLAESEGQGCD

SHADDFTFMLEDIFDLDKLNNVPEDMQENDSSIANNMVENSANVSDQNDWYLELNDIANA

DGLCTTNNVFDDTIISHFPNEGSASQELDGSEVFDNSNRTMGTEGMFEMQQSDDFLNPAV

ASRLGDNTVFYDAPSNDTPYTGDVYMDMNSNDQFSPYIGDFDLVDDLMAYFDASEHDLPY

EVDSLGSYSSDITHPNFAAKVEGSSYPTIKQNLGDPETNIGKGASSSIFTNATNPLETTK

RNSIAATDVQSNDGWNKSFKKHVADMLDSISAPPAMAEETCKGKAAQIFNGSSLIHASAG

MIKIQGVSVNGIPENWTLNKNGEISFIYTYANSNDAVEKSPCFRPLTASAAFRILLRSGL

YIFGIAAVLISISLKLGTGIYSN*

>CepNAC38

MNLLNPTANLAPGFRFHPTETELITFYLNRKINRVPIEFNPIADIDLYKYSPWKLPEL

SNLKIRHEKQWFFFNSLDRKYDGSRRSNRATADGFWKSTGKDRPIYEETESKRIIGMKKI

LVFHVGRAPNGKRTDWVMHEYRGVEDAEIKDNRASFVLCRIFHKSSIGREKMDQCGSRYV

KEEWEDAHDSSESDTNGLVDADGSDQKTDVIFLAPGFRFRPTDEELVGYYLKRKVMGLHL

RVDAIPEINIYKSAPWDLPSLTRVSLTVPNKEGYFFSILDRKYSQNHSTINNRATAEGYW

KSTGNDCPVLSHLGGKKMGMKKFLVFHTGRAPRSQRTDWVMHEYRLEEDEQNSVMCGKGS

TFVVCKIFQKKSFGEQKGRPRSVDPTTEETDEGEIVPVDVLDCAPNPTTDGSPNVTTAVC

TPIVNEAMFAPMVTEENFAPKVTQAHAAQNAELIEVVQKKIHAQASKEVQQGKAQSLYKV

GNHVEIAYMRQQIPVAWYTASVIKVQDEQVLLVEYETLKAKDNSLLSELVSIQYIRPHPP

VREVEHFKVLAEVEAYYNGGWWPGVVATVHDNAKYNVKFMHLEEEIEFDHKELRLLYDWV

DGKWVQASQENKTLESLNHEELRTVEHTQLLHSNNQDHKRKLSHSNGCEIGITSEFTITS

NMENKNGKEIAIKSQHEVFQQFKKAKIEENVEDPFSEENNEELVLELSLPGYKGGKIHED

RKKKKDNCIPRTVESSLNVGLLSKIQENNKSLELSTSSDNSSEAQGLSNPLTSSSDASLV

ESSTSSVKPRFDKLPLEQPSLPFEKHSSLWDTLENMEVFHTMPQNPHFAPLGQHGEAFRE

GMAIGLMLTFSNVVAGIEKLDATDSVEKLALIYKTLIELEAHGFEVQCLRARLMELLKVK

RNKEKLDDDVSSLEDLIEKNGRVDLNLKLDLEVKSDVSS*

>CepNAC39

MESTDSSTGPPAPPQPHPQLQPAQPQLPPGFRFHPTDEELVVHYLKRKAASVPLPVSIIA

EVDLYKFDPWELPSKASFGEQEWYFFSPRDRKYPNGARPNRAATSGYWKATGTDKPIMSS

IGDSGGCSVSAGQMKKVGVKKALVFYGGKPPKGIKTNWIMHE

>CepNAC01

GATTGGTTTGGTCTACCAGCAGGAGTGAAATTCGACCCGACAGATCAAGAACTCGTAGAACATCTAGAGGCAAAAGTTTA

TGAACAAGACTCAAACTTATCACATCCCTTAATAGATGAATTCATACCTACAATTGAAGGAGAAGATGGAATATGCTACA

CTCACCCTGAAAAACTTCCAGGTGTCACTAGAAATGGACACTACAAGCATTTCTTTCATAGACCATCAAAAGCGTACACA

ACAGGGACAAGAAAGCGGAGAAAGATTCAAAACGAAAGTGATCACGGCCAAATACAAAGAGGGGAGACTCGATGGCATAA

AACGGGTAAAACAAGACCCGTTATAGTGAATGGAAAGCAAAAGGGATGCAAGAAAATATTGGTTTTATATACAAATTTCG

GTAAGCATAGAAAACCCGAGAAAACAAATTGGGTTATGCATCAATATCATTTAGGAGAGTTGGAAGAAGAGAAAGAAGGA

GAGCTCGTTGTGTCAAAGATATTTTATCAAACTCAACCGAGGCAATGTAATTGGTCGAGTCATGATAAGAGTGGAAGTAG

CTCTTCGAAAGAGATTGTTGGCCAAAGTAGTCAGCTTGATCATCAAATGGATAACTATCAGTTTGCTCCATACAGTAAAT

CGTTTGATCAGATAGGCATGACTACTGCAGAGGCAACAACAAACACAAGGAATGTTCATGTGCATAATTTGAGACATGAT

CAAAACCAAATCATAGCAGCAACTTCCACTTTCCACATAAACCAACCTACACATCCAATCTCTGCAGTTATATCTCATCA

TCATCGAAGCGGCACTTTGATTAACCATGATGATCTTTATCATATGCCTAGAATTGTTCGCCAGTCGACCAATACATTTC

AGCAACAACAACAACAGCATACTAACATGGAAGAAAGATCTGATGCTGGCTTGGAAGAATTAATAATGGGCTGCAATTCA

TCCGGCAATAGACAGGCACAAACAATTAATCCTCA

>CepNAC02

AAAAGAAAGCATCATATATACGAAGTAAAATGGAAAGATACGAATCAAAACTGGACTTACCAGGATTCAGGTTCCACCCA

ACAGAAGAAGAGCTACTCGATTTCTATCTCAAGCACAAAGTAAACAATAGGAATCACAGCTTCAACATCATAGGAACCAT

TAATCTGTACAGTTATGATCCATGCGAGCTTCCAGGTTTAGCAAAAATAGGAGAAAGGGAATGGTACTTTTTCGTGCCAA

GGGACAGGAGACAGTCTAGCGGAGGAAGGCCAAACAGAACAACCGAACGCGGGTTCTGGAAAGCTACTGGGTCTGATAGG

CCTGTTCGTAGTGCAGCAGATCCTAAACGGGTTATCGGGTTGAAAAAGACCTTGGTCTATTATATGGGCAGGGCACCGCG

AGGATCCAAAACTGATTGGGTCATGAATGAATATCGCCTTCCTGGCTCTTCCAATTCCTCAGTTCCTCCTCCACATGAGG

ACATAGTATTGTGCAAAATATATAAGAAGGCAACACCATTAAAAGAGCTTGAGCACCGTGCAGCTATAGCAGAGGCAAGA

AGTAGAATAAATAACAACATGTTACCAGCAGAAAATGCATCATCAAGCAGTCAAATTGATTACAAGTTTGATACAGAGAA

TAGTTCAAATGAAGTAGATGATATTGAACTATTGATGAATCAAGATGGAGCATCTAGGAATGAGATTGAGATAATTGCTC

CACGGAGCTCAGCAGTTGTTCAGCCGGTGGATGTACCAGAGCTTCAGATTCCGAATAATGGATTTGATTATTGGATGCAA

GATGTAACTCTATGGTCTCCATATGCTAACCTTCTTAACTTCTAACTAGGCCACTTTGAATTCTGTACATTGTTGACAAC

CGTTATATATAGTGTGTTATTTGCAAGAACAGGAAAATGAAAACTGATTAAATGTAATAGAAGCTAGTCTCATAAAACAA

ACTTAAATGCATGTATTATTCATACTTTATTACGCGATTAAAAAAGAAGCAGCAAACA

>CepNAC03

CTTTATCTTGGTAAGTTTTAATTACTCCCCCACCTCTCCCACTTGTACTATAGTCTATTGCTAGCTACACACATACATAA

AAGGGTTAAACACTGGAGAAAAATAAAGAAATGAGAACTGAGAGATGCGTGATATCACAATTGTTGAAGCAAGCATCATG

AGTAGTAACAGTAGAGATGACCATGAGCATGATCTTGTGATGCCTGGTTTTAGATTTCATCCAACCGAAGAAGAATTAAT

TGAGTTTTATCTTCGGAGAAAAGTTGAAGGCAAAAGGTTTAATGTTGAGCTCATCACTTTTCTTGATTTGTATCGATATG

ATCCTTGGGAGCTTCCAGCATTAGCAGCCATAGGTGAGAAAGAATGGTTCTTTTATGTACCAAGGGACCGAAAGTACCGA

AATGGAGATAGGCCGAACCGTGTAACTACATCCGGATATTGGAAGGCAACGGGAGCTGATAGGATGATTCGAGGTGAAGA

TTCTAGACCCATTGGCCTCAAAAAGACACTTGTGTTCTATTCGGGGAAGGCACCAAAAGGAATTAGAAGTAGTTGGATCA

TGAATGAGTATAGGTTGCCACATAGCGACGCCACCGATAAGTTTCAAAAGGCTGAAATCTCACTGTGCCGAGTATACAAA

AGAGCCGGTGTAGACGATCATCTCCATCCTCTTCCAAATTCCAGGGCATCATCTTCAGTAAACACCGAGTTGCCACAAAC

CCTAGCCCCGATTGTAGGAGATTCATCATCGTCTTCACAATCATCAATGGACAAAAAGACAGCAACCAACTTCTTCAATA

CTACAACAACCACCAATTCAGTCGAAGAAGTTGGAACCACACTGCTCACGCAACCAAAGAACACAAATGGCACGTTAACT

TCTCTCTTAGCTCCTAATACTTTTGCTAATTACATCGATCATAACAATGTTGTAGTAAGCACTAGTGCTATAGACCAGCT

TGTTAATCCGTTACCGCTGATGCAGTCTCAATTGCTTCCTTTTGGTATTTCGGATAAGTTATGGGACTGGAATTTACTGC

CAGAGTCTGGAAGAGACCACTACTCAAATTTCAAGTAATTTCTGTAATTTAATTTACATTTGTAAACACTGATTATTAGT

GTTACATACACATTATTATATTTGATCTGTTATGGCTACATAAAGTGGTATTATTGTATTTCAAGTTGTATTATAGGTTA

TGTAGAACAAATGACTATATTATCAGTTGGAAAGTTCTAGGTATTTGATTTTTAGTGTATTGACTTTCTGACATGCATAA

AAATACAATGATGTTCCACATGAAAATGATTAATAAAGACTTGGCAGAGGCCACTTTGCTAGATTTTGG

>CepNAC04

CCACGATTAACATTCACAACATCCAAACTTTCTTTACATATACATATATATTTCAATGGAGCAGTTAAATCTACCGCCGG

GCTTCAGATTCCATCCAACGGACGAGGAGCTTGTCGTACACTACCTCTGCAAGCGATCCGCCCACCAACGCCTGCCCGTC

CCCAACCTCATAGCCGAGCTCAACCTCTACAAGCACGACCCCTGGGATCTGCCGCACCGGGCCCTGTTCGGCGAGAAGGA

GTGGTACTTCTTCACCCCTAGAGACCGCAAGTACCCCAACGGGTCCCGGCCCAACAGAGCCGCCGGCCGCGGGTACTGGA

AAGCCACCGGGGCCGATAAGCCCATCGTCCCTAAAGGGTTCACGAAGCCCGTCGGGATCAAGAAGGCCTTGGTCTTTTAC

TCTGGCAAAGCGCCTAGAGGAGTCAAAACAGACTGGATCATGCATGAATACAGACTGGCCGATTCCGTTACTAATAGCAA

GAAAGGGAGCTTGAGATTGGACGACTGGGTGCTGTGTCGGCTGTATAATAAGAAGAATAACTGGGAGGAATCCAAGCCGT

CCGTTGGATCCGACGATAGTTATGTGTCGGATGTGGTGGACGAGGACATGGAAAGGATTTTAGGGCAGCAGTCGTGTCGG

GAGATGGAAGGCTGTGAGGTGAAGATGAAGAAGGAGGAGACGGATTGGTTCATGGATTTTAGTTTCGACGAGTTGCAGAA

TTCGTTTGGTGGTTTCGGGTCCTCGGGTGCGGGTACCGGGTTTGGATCTTTGTGCCCGGATTATTTGTTACAGTGATGGG

GGAGGTGAAGTTACAATTGGAGAGGTTGTAAATAAGTTGACCTGACTGCGGACTGTGTGGGGCATCAAATTTTGCTTAGT

TATGGCTGTTATGTATTCGTCTATGTCGGTATTTAAAGTTGTTCATAACCAGGAATACAGTTTTGTTTCGGACAAATTAT

ACATCTTGGAATCATGGTTTGAGGACAATTTTTCTGTGAAATTCTCACGTTATTTTTCTTTATGCTAATATCTTTGATAA

ATTGAAACTTTAATCCAAGTTATTTTTTCCTACTCTAATTACCATTTTTGCTGATTTACATCAAATCGTAATGGCTTATA

GCACATTACATAAATAAATAAATATAGACATCAATATGAGCACATTACATCCCTGTTAATCCATTATCTATTTCCTATAG

CTTGGCATACATATTTTGTTTAGAGCGATGTATTAGAATTGAGAGATGTAGTAGAATTTTGATACGCCTTTGATTGATTA

TGAAATCCA

>CepNAC05

AGCTTCTTCTCCATTTTCAATTCCGAATTCTTCTCTCTTGGGTCTACAACTTTCTTGTGCTTTGCAATCATCATTTATTG

TTAATTAATTCATTTCAAAGTAACAGTATATTTTAAACAAGTACAAGTGCAAGGATGGACGAGTCTATAAACTTGCCACC

AGGTTTCAGATTTCATCCTACAGATGAAGAAATCATAACTCATTACTTGTCTCCTAAAATTCTCAATCAAAACTTCACTA

CGATCCCTATTGGAGAGATCGATTTGAACAAGTGCGAGCCTTGGGATCTTCCTAGTAAAGCAAGGATGGGTGAAAAAGAA

TGGTACTTTTTTTGCCAAAGAGACAGAAAGTACCCAACTGGAATGAGAACTAATCGAGCTACGGAAGCAGGATACTGGAA

GGCTACTGGCAAAGATAAAGAAATATTCAAAGGCAAAAACAATACCAAGGTTCTTATCGGCATGAAGAAAACGCTTGTAT

TTTACACAGGTAGAGCCCCTAAAGGAGTTAAAAGCAACTGGGTCATGCACGAGTTCAGACTTGAAGGCACATCTGCTGAG

GCCAACTTTCCTAAACCCGCAAAGGATGAATGGGTGGTTAGTAGGGTGTTCCATAAGAGCAACGGAGCAAAGAAAAGTCC

GATAAGCGACTACTTTGGTTCTGGGAGTTTACCAACTTTAATGGATCCGACTCCGTTCGTAAACAACGAGGATCTAATCA

ATAATTTCGACATGATGAATTCTTCATGCTTTTACAATGTTCCAGGATTAGATGATGCACAGAATTTCAATCAAAATTAT

CAACAAATGGGGCAAGTGAATGGTTCAACTTCAAACTACAACTACTTGCATCATAATCCGATGATGAAAATGGAGCAATT

TCCAAAAAGATCAATGGTTAGTGATGACACCGGGCTTAGCACAGATAGAAACACAGAAATTTCTTCTGTCGTTTCAGGCC

ATGAGATTAAGAATGAGTTTGAAATGCCTACTGTTGGTTCAGTGTTGGATTTGGAAAACATGTGGAAGTTCTAAGTATGT

AGATTACTGATGCTAATGTTTTATAAAGCCTAACAAAAAACTTGAAGGCGATGATGATGACGGTTGTTTTGTTTATGTAC

ATAACATAGATTGAAGATTTGTGTTTTAACGTTATACAGATTTGTTAACTCATTTTATAGAGAATTTGCATGAAAAAACA

TATATTGTGTGGGTGTTCATTAGGTGATGATTACTAAATTCTATTCTTTTTGTCGAAACATTGTGATAAGTTATTCCTTG

TTTGATTGCTA

>CepNAC06

CAATAAGTTGCAAAAACAATAACCCCTTCTCCAAGTCTCCTCTTTACATCTCCCTCATCGGATCTCAAAAATGGGAGCCG

CACCCGACATGAGTGGGGCCCAATTGATCCCGCAAGAGGAAATCGAGGTTGATAATTCGATCAGCGATTGCAGAAACTGG

CCACCTGGCTTCCGATTTCATCCGACCGACGAGGAACTCGTTCTGTATTATCTCAAACGTAAGATCTGTCATCAGAAGAT

CAAGCTCAACATGATCGGCGATGTTGATGTTTATAAGTGGCACCCTGATGAATTACCAGGAAAATCGTTGTTGAGAAGCG

GTGATAAACAATGGTTCTTCTTCAGCCCTAGAGACAGGAAGTATCCAAACAGTTCAAGGTCTAATCGTGCTACAGCGAAT

GGATACTGGAAGGCTACAGGCAAAGACCGTACCATCTCTCAAAACTCCAAACCCGTTGGTAACAAAAAAACTCTTGTCTA

CTACCATGGGCGGGCACCAAAAGGACAGCGCACTGATTGGGTGATGTATGAGTATTCATTAGCTGAGCAAGTGCTTATTA

ATCTAAAAAACGTGCAGGATTATTTTGTTCTATATAAGCTTTTTAGGAAGAGTGGACCTGGTCCTAAAAATGGAGAGCAA

TATGGTGCACCATTTAGAGAAGAAGAGTGGGATGAAGATGTTGTAGATGACAATCACATGAATCAAACTAACAGCAACTT

CCAAATGATTGAAAATCCACCTAACAATTCAGTTGACAATGAATTGCCAAACGGGGATATGAACGCAAACCAAAATCATG

ATTTGGATAGAATGTTGTCACAAGTAGCAACTGAGCTCGATGTCGTTCCACTAAATTCTGAGTTGCATTCTGTGAATAAT

GGTTCTGGAAACCAGGTTGATAATAATGCAAGAAACGAGAGAGCATTGAATCTAGAGCAGAACTCACTTCCAAGTTATGA

TTTAGAGGATATTTTGCTGCAGCTGTCAAATGAGCAAGATGTGATGCATCAACTTCCTGATTCTACTTCCTATGCTCCAA

ATGTGTTACAGGGAAATGGTGCAAGGGATGATGATCTTGATATAAACAATTTTATGACCCCATCGCATTACATCAATGAA

GTCCCTGTCCAACAAACTAATGCTGAAGAAGAGTATCTTGAAATTGAAGATTTAATAAATTCGGACTGTCAAGCTCAGAA

TCCAGATAATATGAACTACAACAGTGAGCTCAATCATATTGGTGATCAGTTTGTTTTTCCTGATGAATATTTTGATACAC

AGATGTTTTTAGAGGATGCTATGGGATCTGATTCAGGAATGAATCAACAGAATACATACTGGAATGATTTTCTAAACAAT

GAGCAACAGATGCCAGCTTCTAACATCACTACAGATTTTCCAAGGCTTGAGCAACATTTTAGCTCCTCGACTACATCTGT

AGAGCTATGCACAAATGTTACTGCATCATCTTCAGGTGTAATGGGTGCTAATAACAATAATACAACAGAGTCGTGGTTTG

ATTCAAATCTTTCAGCATTCTTAGGTTCTATCCCTAGTAGCCCAGCATTTGCATCTGAAAATACACTACTTACCAAAGCA

CTAAATCGAGTTTCTAGCTTTAGAGGAGGTCGTAATAAAGAAATGAGAGATCCGTATGCTGCAGTTAATAATAACAATGG

CATTATTGTTCGTAGAGGGCGGCAAAATGGTGGATTTCTATTTACCTCTTTTCTTGTAGGGCTGGGAGCAGTTGTTTGGT

TTTTAAGTATTGGCATCACTGTACAGATCTTCAAGGGATTCTGGAACAGATACTTCTCTTAAGGACGCGCCTGATACTTA

TAACTATTAATGTACAATTTAGAGTTTTTGGTGGTGGTGATGGAATTTTTATCATGGTATTTATGCTATCTATTACTGCT

TTTTTAAGTTGGGTTATAGTCACTGTATTTTATTTTCACCAAGCATGAAAACTATGTAGGCTAATGCTTTGAGCGAGTCT

TTTGGTTATGCAGATTGATTCACATATTCAAGTTGAAGAACATGGGAAGATAAATGCCACTAGGCAAGTAAAACATATTC

G

>CepNAC07

TTTCTATGATCAATATATCTTCACTAGCACAGCCACTCATTATCAAATAAGTTAGAAAAGTCTCGTCTTTCAGAACTTTT

GTGTAAATTAGGTATCTTTTCGTCTCGATTTTATTTATGTTTCGTGATCTTTATGTTTTTTTCCCCAATTGTTCGTTGTT

AGTTGATTTTGATAATTCGGTTTCTTGATCTCCAGTACAGAACGAAATGATCTTATAGGATCTCGCAGTCCTGAAGTTAA

AGAGCTAACCGGAAACGAAGGATTTTATTTCAATTTTTATTTTCTTTCTATTCAGAATTACTTTGGGGTGTGTGCTAATT

CTATGTTGAATGGCTGTATTTAAACAATGACAAACTTAACCCATAGGTTAAAGTTGATTTATTTATTCTTTCGTTTATTT

ATAGTAATATCATGTTTGATCTGAGCTGATTTTTAAATAGATGGTTGTATACAAAAATGCGTAGCTTCTTTCTGTCTGTG

ATTGGTATTCAATTATTAAATCTTCGTATGATTAATGCTTTTAGTTCAATGCGGCACACATATGTGTACCGATGTATACC

TTCCTCTATTTTATGATGGCATTCTCTCTTCTGCACTGTGCATTTAATCAAACTTTTAGTCTTATGTTCTGCGTCTACTA

CTTTAGTATGTGGCTTTAGTATGTGACTTGAACATGTAAGTTTTATCTTGTTCAGGATATATATACATAACACACATGCT

TATGTTAACGTGTTTTGATTTCAATCATCAGTTGACATCTCTATTTAAGGTGTGTTATTTTTTCTGCAGAACATAGTGGA

GATGGAAGCCTTGAACCTCTTGTCTCTCCCTCCAGGATTTGGCTTTCATCCATCAGATGCTGAGCTTATTACACACTATT

TGAAGAACAAAATTCTTGGGAACTTTCATCAAAATGAAATTATACCATTGGTTGACATATACAAGCATGAACCTTGGGAT

TTGCCTGCACGATGCTGTGTTCCAACTAGGCAGGCCAAGTGGCACTTTTTTTCCCACGGGGACAGAAAGCACCCAAAGGG

TACACGCAAAAATAGGGCTACAAGGGCAGGGTTTTGGAAATCTACTGGTAAAGATAGGGTCATAAAGCATCAAAACAGAA

ATGTGGGTACTAAGAAAACTCTGGTATTTCATGAAGGTAGACCCCCAGTTGGCAAACGCACCGCATGGATCATGCACGAG

TTTTATGTTAATGACAAAGAGTTTATGTTTACGCCAGAGATGAAGGTACCCTTTTATCATTATGCACATCACTTCTATTA

TGATTCCAATTTTTTTTGAATAAATCGAGTTGTATGTTGTGCACGATGGGTGTCCGATTTGTAAAGAACCAAACTGAACA

GAGAAAGGTGCAGCGTTCATTCAAACCATGGCTATTTCATATACAGTTTGAAATAGCCTTGAATATGTTAGGCAAACATA

TATTCATCAGAATTCATATCGTGGATTCTGATTTTATCCATAAAAGATAGCTCATCTTACAAGGATATGTCAAATTAGCT

GTGCAAAGGTAGAATCCAATGTTGACATCAATACTTTTGAAAAGCGTGGAGCCAATGCGTGCATATTTTCTCAAGTTTTT

GAATAATGTTTGATTTTACTTTGTGTTTGTGTGTGTGGTTTCTTATTTGGCGTTCGAACTCCTATAAGGCTGTAAATACG

GTGATAAAACCTATGATTTATTCAAAGTGTATGCTCAGAATTTACGAACACACACACATGAGAATTATTGAATATCTTCT

GTAATCTTAGCTCTTACAATAACAGCTGAACGTTGATAGCTGCATGATGTAAATCATTAAATGGATTCATTGCAGATATT

TAGTATGTTCTCTCGTCCTTTATTAATATTTTCTCTTGCATTATCTTTTGATAGCACAATCAGCTTCCAATGACCAATAT

TTAAAGTTTATTAAGGGCAAAAATTGAAGGGCTTTTAACATTCCTCTTTGTACGTATGTTGTATGTATCCTTGCAGTATA

TATTGTACTCCTAATTTAATTCAGTTTGCATGCCTTCTGGACTTTGGTTTTTGGTATAGCCTTGGTGACATTGTTCAAAC

GACCCTCTAAGAGAACACAATTTTTCATCTAATATAAAGGTAATGGCAAGTACATGCTTGAGTATGAAATGTGCTATGAT

GCTTATGTATTAACGTTCATTAATTTAGAAATATACCCATTTAGTTGACCAAATGAATGGAACATGTGATAGAAGAACAG

TATCTGATTGCTCCACTTTCATCAAATAGGTTTTCAATCAGACATGACGTAACTTTTTTTTCTTCCTTCTTTCCTTTTAA

ATTACAGGACAGTTATGTCCTATGTCGTGTTGCCTGTCGTGATGGTTGGGAGCCAATGAATACGTCCAACTTGGTTCAGG

CACCATCTAATTCAGTTCATGCACCATCTAATTCAGTCGAAAGTGAAGAGGAATTGAATGCATGGATTGCGGAACTTATA

GATCCAGACTTTACGGGCAGCCCTATTCATTCTGCACTCAACTGTGTCGATACGGTGGGTAGCAGTGCCATCCTTGAGGC

CAATCAGGAAGCAGTCAATTCACTACCTACTCCTGAAATTTATGATGATCCAGATCACTTATTCTCTGTAGAAGAGTACA

AAAACATTCTGGAATCTGAAAACCAAAACACTGACATTTTCGAAGGTTTCGACTTTGACGCAGACATCTTCATCGACCAA

TATGACGATATATTCAGTATCTTGGAGAATCACGAATTTGCACAAAGCAACAACTACATCAACAACGACGATGAAGTGAC

GGGAATTGAAGTGAGGCAAAGAGAAGCAGAACCCAGTTCTTCTAACACTACAGCAATAAGTACTGTTAAACTAAGGCTTC

AGGCTAATAAACTGGAAAGGAAAAGCAACAACGAACCCATTAGTGAGTTTTTCAATGTTAATGGTGCAGTTTGCCTTAAC

CGCGTTCATTCTATGAGGGTTAACAGTGAGACCATGGTTCGAAATACCGAGTACAAGGCTAGACAGTCAGCCTTTGTGAA

CTGCTTCTTTCTGTGTGCTTTCATCACTGCAGTTGTTGCTATCGTTTGTTACTTCAAGGTGAAGAATGCTCGAGTATACA

GCTTGAGCTGAATGAGCGAAGAAGATGACATTTTCGGTCGTTTGTGGACTGAATTTTTTCGCCTTCGTATAAATACTGAT

GTAGTTGTATATCATATTGTGTGCGTTTTCTTAATGTTCTTGCTTAGTTTGTACGTAAGATTTGTATTTACGTTCTTAGA

GGCATGTTTACATGTATTTCTTAGTAATACCGTAATAAAACGTCATGCTAATGTAGTACTGAGGGTATTTCTCATGTTTT

AGATATCTCAAGATACGGGATCATTGTGCACAAAATGAAATATCTTATGAGTATGTTATGTCATTTCAATATGAATAATT

TAACGCTTTATCGGAG

>CepNAC08

CCATCCTCTAGCAACAGAAAATTCAACCTTAGCTCTTTCAAATTGAATATTCTTCCTCCTCCAATAAAAAGATATGACAA

ATCAAACACCGGACCTCCCTCCTGGCTTCCGTTTCCACCCCACCGACGAGGAACTCATCCTGTACTACCTCCGATGTCAG

GCTTCCTCCTCCTCCTGCCCTGTCCCCATCATCGCCGAGGTCGACATCTACAAATTCGACCCTTGGAACCTTCCAGATAA

AGCCATGTTCGGCGACAGGGAGTGGTATTTCTTCAGCCCGAGGGACCGGAAGTACCCAAACGGCGTACGACCCAACAGAG

CCGCCGCCTCCGGCTACTGGAAGGCCACGGGCACCGACAAACCCATCACCACCGGCAGCGAAAACGAAAACATCGGGGTG

AAAAAGGCTCTCGTCTTTTATAAAGGGAAGCCTCCCAAAGGGGTGAAGACCAACTGGATCATGCACGAGTACCGCCTCTG

CGATAACAAGAACAAGAACAGCTACCGCCCCATGAAGCTCAAAGACTCCTCCATGCGCTTGGACGACTGGGTTCTGTGCA

GAATATACAAAAGGAACGGCCATGACCAGGACGACGTGCACGAGGAAAGCGACGTTTCGAACACGGCTTACATGGCCCAA

GGTTACGAGGATGATCCGATGAAGATGCATAAGTCGTTCTCTGTTTCGGAACTTCTTAATGAAACGGACATAGCAGACAT

ATCTCAGTTGCTTGATGCTCCGACGACCAATACTGGTATTGATAATCAAAACTTATACGGTATGAACCAGAGAAGTTTGA

AGAGACAGAGACCGGAGGAGGAAAATTACTTGCAGGGACAAAAATTAACCAACATTAATTCTTGGACTGCGGGGAACTTT

TCCAGCCAATTGGATTTCGATAGCCAGGCTTATATCAATCAGCAGTTGATGTTTAACTCCCAGCTGGATATGCAGCAAAA

ATGGTTAAGTTAAAAGTATTTCTGTATTTTTAGGAAAAAAAGGAAAAAGAAAATACATATTGATATTGAATAATTAGTGG

TTTGTATTAATTAGTTGGATGGGTAGTGTAATTAACTCCTATTTTGTAGATTTTGTGTATAGTTTGAATTAATGCAAATT

TTAACTTCTCTGATCGTGTACACTACGAAGAGTGCTCTTGATCCTACTGTAAAGAGTGGTTAAATTGATCCTACTGTAAA

GGGT

>CepNAC09

AAAATCAGAGTGCACTTCATCTCGTCTGTAAACTCAAAATCCAATGATGAAAATTCCGACTTCAGAACAGAACCTCTGCT

CCACTCTTCCACCAGGCTACCGATTCCATCCGACCGACGGCGAACTTATTACTTATTATCTTCACCGGAAAATCTCTAAC

ATCCCATTTCCGGTCGACTTAGTTCCCCAAATCAATCTTTACGGTTACGAGCCATGGCAACTTCCGGCTCTTTCAAAGTT

CACTGTGACCGGCAAAGAATGGTATTTCTTCAATCTAGTCTCCGGCCATAAGAAAGGAAGTTTTGTGCGGCCGAGTAGAA

GCAGGACAAATGTCAACCGGGAAACTCCCGAAGGGCAGTGGATATCGGGACTAAAGCGGGAGATTTTGGTAGATGGGAAG

ATTGTTGGGTGGAAAAGGAAATTCACCTTTTTTGCCAGGACCAATGGCGATAGGAAAAAGACGGACTGGTACATGATGGA

GTATCGTAGCAGCAAGGATGATGACAGTTATCCCAAGGGTGCTTCACTTGTTATTTCTAAGATATATGCAAAGGTCACAC

CTGCACAACTTCAAATTGAACCCACTTCGGCACATAAAGTTGTTGCGAATGAGGGCAACAGTTCAGTACAGGACATAGAG

CTTGATACGACACAGGACAGAAAATCATCAGTTGATGGGGAGCAAGATCTTTATTCAACAAAGCTTGAACAGGTATCAAA

AATGGCAATGCCCAAAGCTGGCCAATCAATCCTCAACGATGAGACTTCATCCTCAAAAACATTTACGCCATCGGGGTACC

TTTCTCTTCCAGGATTCTGGTTCTACCCAACAGACAATGAACTAATAGAGTACTACTTGAAGCATAAGGTGTTAAACCTA

CCTATACACGATAATGCCATATCTGAGATCGATTTATTCAAGTGGGCCCCATGGGATCTCCCTTCTCAATCTCGTCTTCA

GCATAAAAACATGCAATGGTTTTTCTTCAATCTCCATGATCGTACACACATTCACTCGTATTATAATGTGAATAACCAGG

GTGTTGAGGGTTACTGGTCATCAGGCACTGACTTGCCTGTACATAATACATCGACAAATGCAATCATTGGAATGAAAAAC

TTCTTCGTTTTTCACCAGGGAAAATTCCCAAATGGGAAGAAAACTTATTGGGTGATGCACGAGTATCGTTTAAGAGAAGA

TGGTCAGATAGGTCCTATATGTGTTGTGATTAAGATTTGGAATGTAAGCATCCAATTCAAGCTAACTGCATCTGAGACGC

ATGAAAAGGAAGTCCTAGCTCCTGTTGATAAATCAACATGTGCACTCGTTTCCAATTTAGAAGCTGCAAAGGGAGATAAC

GTCTTGCATTCACCCAACAACTCTGAGCAGGGATCAAGTACACCTTTTATGGTTGGTAGCGTGGCAGAAGTCGCCTATAC

TCATGAAAATGTTCCGATTGCATGGTATACCGCTACTGTCACAGAGATAATCAACGAAAAGGTTTTATTAGTTGAGTATT

TGAGCTTGAAAAATGAAGATGGGTCTTTGTTGCGTGAAACTGTTTCAATACAAAACATTAGACCGTGCCCTCCTTCAACC

CAACAAGTAAGAGATTTTCAACTGCTTGATATAGTTGAAGCATTCTATAATGGTGGATGGTGGCAAGGAGTTATAGCCGC

TGTTAAAGATCAGTCGAAGTACAATGTTAAACTCATGCTTTTAGAAGATGAAATAATATCTAGCAACACGGAGCTGAGAG

TTGTTTATGATTGGATCAATGAAGAGTGGATTTTGGCGTCACAGGATGAACCTTTAGAATCGCAAAACAATGTTGAATCA

TTGCATGCATGCAGTCCTCTCAATAAACGCAAGGATCCTGATCCAGTTGCTACCTCGATGAGTGATGTGGATATTTCTGG

TTTGTTACCATCATTTAAGAAAAACAAAAACGATCAAGAATCAAGAAGGCCTATCCTTCGAAGCAAGAAAGGAAGGAAAA

GTGAGACAGTGGAAGATTGTATAAACTTCTCTGCTTCAAGATGTGTTGAAACTTCTAATGTTCTGAAGTTAGGTCTTGAA

GTTCCCACCTTTATACATGAAGAAAAACAATCTAATATTGACACTAACACAGCGGTTGAAAAGATGAGCGAGGATAGTCA

GCAAATTGGGGAAGTACCTAAGACTGACCAAGATGCAGTTCAGGATAAATCCCTGGAATCGCGAAACTATGTTGAATTAT

TGCTTGCTTGCTTGCTGCTTTATGCAAAAACACAAGGATCTCGATTCAAATACTACCCTGACAAAAGTAGATCAATATTC

TTTGTTTCTCACCATCATCTTCACAAAATGGAAACAATCATGAATCAATAAGACCAATACAGGAAGGAAGAAAACAGAAG

GCCATCTATTTGAAGCAAGAAAGCAGAGAAAGGAACCATAGAACTGCTCGAATCATTGCATGCTTGCTGTTCTATGCATA

AACCTGAGCATCTCGATCCAATCACTACCTTGGCAAAAAAATTAAATTCAGTCAGTCTATATTCTTTGTTATTCACCATC

ATCTTCACAAAATGAGAATGATTATGAATCAATTGGACTAATACTGGAAGCAAGAATGCAGAAGACCATCTATTGGAACT

TCTAATGCTTTGATTCAAGTGCTACCGGAACTTCCAATGATTATGAATCAACTGGACCGATACTGGAAGCAAGAAAGCAG

AGAATGTGTGATGGCAGATTTTTTATCTTCAGCAATTGAAAAATGCTTTCGTATGTAGGGAGCTTATTTTTGTCTATTGT

TTCAACAGCATTATTTTTGACTTTTACAATCATACTGCAGAGAATTGCACAGAGGGATGGCTGCTGGTGGTCTTAATTTT

TTTACAATTTTGCTAATTTATATCCTTGTGCATGCTCCATATATTGCCTTGGATGAATCCAAAGTAAGAGAAGGGTTCAA

GATATGATTCTGTATGGTTTCTTTGTTGTACGGTTAAATTAAATAGGCAATTAAGATATCATTCTGCA

>CepNAC10

GAAAAATCAATTGAATGAAATAACTCATATTTACGAACCGATTTCGAATTTTATATTTTTATCCGTTTTAAGTTCTCTCA

GAAATTAATATGGCGGAAATCGGTGTGAAAGGGGATACAGGCTGGGCAACTAATTTAGCTAAGAAATGGATAAAGTTGCC

TAGAGGTGTTAAATTTGAACCGTCCGACGAGGATATAATCTGGCATTTACTTGCAGAGATCGGAGAAGGGGATGCTAAAC

CTCATCCTTTTATTGGTTTATTTATTAAATTCTGGAACATAGATGGTCCTCATAAGATACCTCCTGGTGTAAAAAAAGAT

GGAAGTATGTCCTACTTTTCCCATAAACCATTTACGCCACAAGAAATTCGGAGTAGAAAACACAATGAAGGAATGAAAGA

TGGGTGCGTCAATGGTTTCTGGCGTAAGTTAGGTAATACCGAACATGTATTCATGGATGGAACTCAATATGGTTCAAAAG

AGATATTTGTTTTGTGCAAGTACCTGAAACGGCATTCAAGGTTCAAGAAAACTAATTGGGTAATGCATCAGTATCATATT

GGCACTGGGAATGTTAAGGGTAATCTTGTCATTTCAAAGGTTTTTTATCAGCTGAATTCTGAGCAAGGCGAAAAGACTGT

ACGAAATTTTGGTATATCAACGGAAACTGTTTTAAGTCTTGCGACCAATGGACGTCAAGTTCATGCTTATGATTCTTGTA

TGAAATCATTAGCTGTGACTAATCAGAATGATACTATGGGTAATCATCATAATGAAGCCAATGCATCTCAGGGTGATGTT

TCACGTGCAATGGTAGAGCCCTCTAATTGCATTCAGAATAATCCCATGGAAGTTGCACCCAGTATTCCTGCTGCTGTAAG

TGAATCTGGAAATGGCGAGGTTGATGACTTGACATTGCAAGAGCGATATAGACAGCTGTTTTCCAATATAGACAAAGTTG

AACCTGAAGCATTGCAAGGACAGTGTAAATCAATGTTGTGCAATATGAATAATGACACTGGTCATAATCTTATTTCTGAA

CTTAGCGAAGATGCTGGCTCTGGAGTTCACGTTGAGGAAAATGAGGTTCAGATGTAGGTTTGCATTGATCTTTCATTTAT

TTTTTTGCAAGTAGGTTTCCCTCTGATATGGTAATATTTTGCTGAGAAATATGAGTTTGCTTTGCTTATCTCTACATGTT

CCCATGTCTTGAACCAACTATCTCTTGTCCAAGTGGAATTCAATGAGAGAAAAGGGTGGATAGACAAAAGAGTTGCGAAG

ATATCTCTATGATACAGTATTCCTTTTCGTTACCAAACATTGATCGTACAAAGGAATCATCAGCGAGGATGGTTAAGGAT

GAGGAAAACGGTGACCAATTTTTGCACGGTGATGGCCTATGTATCAAAAGTGCAGTTGGTCATCATGTTTTATCAGTTCC

CTGCTGTACTAATGAGTCATATGAAACTGGTGCCGTGGGATATTGTGACCAAGGTAAAGCTCAAGTGAAGGCTATGATGT

CTACAGAAGAAATCCTCATAGACGCAATAAAGCAAGACAATTATCAAATGTATGATATATTTGACTATCCAGATATGAAT

GTTTATACCTCTTCTTTGAGGGATACCTGCCTTCCCCCAGATACAAACCCGGAGGCTTCTTCTCCTGGAGTCAGTCAAGT

AAAGCATTTACTAAAATGTAATAATTGCAGCCAACCTAGTGATACAACTCTACCTATAAAGACAGAACCTTCTGATGAAT

GGAACATGAACTCTATCCCCGTAAAGAATGTTGATGAACCTTTTAACACTCTTCATGAAATAGACGTGACTTCCCAAACT

GAAATTCATAATTCTGTCTTACCAATCAAACCAAAGCTTGAACCTATGGAAATTAATCCATTACTCTCTTGTGAGATTAA

GATTGAAAAACACACCATGGAAAATAAATCAGATGATGAACTAGACTTCTTAGACGATATTCCATTAGATGAACGAAGGC

GGTTGCTAAATTCCACTAGCTTTACAAACATGGGCGTGGATGTAAATGCAAAAATTCCTCTGCAAACTATACTGCCTCCA

GAATGCGATGCTCCCAATGTCACAGATGCTAAATCAGGAAACTCTTCGAGTAAAAGAAAAAGGAAAAAGACTGCCACGGA

TTCAGTTGAAGTTGCTCTTGAGGAGGATGCACCTGGACTTTTACAGATGCTACTTGAGAAGGGTATCACTGCTAATGAAA

TTAAGCTTTATGGTGAGGAAGAAAGCGAAGAAGCTATATCGCTAGGTTCAACTGATGGTTCTTTTCAGCACTTTGAAGCT

ATTCTTGCGAAGTTGTTCCCTGAAGTTCCTTCGATATTGAAGTTCCCATCTGTACAGAAGACAAGAGCATCAAAAGTACT

TTACTGTGTAGCCTGCTTGCTTTCCCTTATTGAACAGGCTCGGTATCTCCACTTTAGGAAGAATCCAGTTGAGTGGGGAT

GGTGTAGAGATCTACAATCTTTTATATTTGTTTTCGAAAGCCATAATAGATTGGTATTAGAACGCCCAGAGTATGGATAT

GCAACATATTTCTTTGAGCTTGTAGATTCTTTACCAGTTGATTGGCAGATTAAAAGGCTTGTAACTGCAATGAAGCTTCC

ATGCTGTACTAGGACTGTCCTCCTTGAGAACAAACCCCTAATAGTCGGAGAAGATATGTCAGAAGGAGAAGCACAAGTCA

TGGAGGAGTTCGGTTGGATCCGGAACACCGGACTTGGAACAATGCTCCGCTTTTGCGACAGAGTGGTACACGATAAAAAA

AATGAAAGGTATAGCTTAGAATGGAATGCAAAGATTGGAAAGTTGCTAATGGATGGTCGGCATAATGGGCGTACAATACT

TTCCAACATGCCAAAGAGATTCAAGGTGGAAACTGTAGATATGGATATGGAGGTCAAGCAAGAGCCCTACTAAGTCTGTA

ATACAGCGATAGCAAGATGACTTAATAGAATATATATAAATCTTGGGAACTAGAAAAATATCTGCTGTATTAGTTGGAAT

GACTAATCTGTCTTTTAGCCTTTTACTTTTACATAAGACATGATCTCTTTGATGCAGGTGATCCTACTTATTTCACTATT

GTTACGTAAATAACAATAATCAGTTGAGATACATTTGTAGTTGGATC

>CepNAC11

ATTTCTTTCCCTTTCTCATTTTCCTTCCTTGCTAATCTTTTTTACTCTTTCTATTGAATTTTGTTTATTGTTTTATAAAA

TAATTTCAAAATATGAGTTACTTGAGCATGATGGAAGCTACAATGCCTCCAGGTTTCAGGTTTCATCCAAGAGACGAAGA

GCTCATATGTGACTATTTAGCAAAAAAGATCGATGGAGATAATACTGATAGAAGTTTATATGATGGTTGTCCTACGTTGA

TGAGCGTGGATTTGAACAAGTGTGAGCCATGGGATCTTCCTGAAATAGCATGCGTAGGAGGAAAAGAATACTACTTCTAC

AGCCTTCGGGACCGAAAATATGCAACTGGGCAGCGAACAAATAGAGCAACCATATCTGGATACTGGAAGGCCACTGGAAA

AGACAGGCCTGTATTTCGAAAAGGAGTTCTTGTAGGGATGAGAAAAACTTTAGTTTTCTACCAAGGAAGAGCTCCTAAAG

GTAAAAAGACTGACTGGGTTATGCATGAATTTCGAATGGAATCACCAGCTGGTCATACCTTGAAGCTTCCTGTAACTAAG

GAAGATTGGGTGTTATGCAGAGTATTCTATAAAAGTAGAGGATTAACATCAAAGCCAATTACCATGATGAACACTACAAA

TTATGAAGATACAATTGCATCACCGTCACTACCGCAATTAACAGAAACATACATAACATTCAACGCTTACGATCAAGTGC

CCTGCTTCTCCAATAATCAGTTTCAGAATAATCCAATGGCTATGATTGGAAGAAGCATGGCAATAAAAAATGATGCAGCT

GCACAAATGGATGGCTTCGATCAACTGAGTCTGAATCAAGTTGGTTGTGACAGGAATGGAATTGTAGATGTTCTAAATCA

ATTGACTAAAATGGAAGAAAAAACAAAAAGAGAGGCACATACTGATAGTTATGGAGGAGGAAATATAGATAGTTATTTTA

GTGAAAATGGTGGGTCAAATATGTGGTAATATTTTTGAAACTAGGGTTGAATGAATGCATGTTCATTCTTCTATTATATT

TGTACATATATTTGGATTCATGCTGGTTATGTAAGATATTTGCTTTTATATATAATTGTAGATTATTTCCCTTTTTTTTT

TC

>CepNAC12

AATAATTTTGGTGCTCTTTGATTTGAACCCTAATCCACACTCACACTCTGCTCTGGCAATTTAGATGATAAAATAGCCTA

CCTGATCTCTTTTTCTACTATTTCGAAGACGGCTGATCAAACCAACACGCCTTTTCTTTTCCCTTTCCCTTTATCTTCTC

CAATACACACAATCATTCTGATATCCGATAAAATTCAATGATCCCTTGCGAATCATCGTCAAGCTCAACCCGATTAGCAC

CAGGATTTCGATTTCATCCAACAGATGAAGAGCTTGTTAGCTATTATTTGAAGCGCAAGATATCAGGTCTGCCTCTGCGC

GTAGACGCGATTGCGGAGATCGATTTGTATAAGAAGGAGCCATGGGAGTTGCCTTCTCTCTCGAAATTGCAAACCCGTGA

CATGGAGTGGTACTTTTTCAGTCCCTTGGATCGGAAGGATTCTAATAAGTTGCGGACGAGCAGGGGAACTATGGAAGGAT

ACTGGAAAACTACAGGCAAGGATCGTGCTGTGAGGAGTACGTATTCTAATGTTGTGGTTGGGATGAAGAAGACGCTTGTT

TTTCATGAAGGGCGGGCGCCAAAAGGGAAGAGGACTAACTGGGTTATGCATGAGTATAGATTGGAGAGTTCCGAGAATGA

TGCTGCTAGTTCATGCGAAAATACTGCTCTTGTGCATCCTCAGGATTCCTTTGTTGTTTGTAGAATCTTTCAAAAGCATG

GTTCTGGACCACAAAATGGAGCTCAGTATGGTGCGCCATTTATTGCGGAAGAATGGGAAGATATTCATGATAATAAGACT

CCAGAATGTGCAGAAAGGCACGGTAATGGCCATGATGCAGCATTAATGTGTCTCACTAATGAGCAGTACTATGCCGAACT

TGATATTGACATGAACCAAATACTGGCAAATCATCAAGATAACAATGAATCGACAAACTCAAAAGATTGTCAAAGTCATG

CAGATGAACCAACTCAATATCAACTAGTAGACAACCCAGTTGCAGACCATCTATCTGGGGAGCAAATTTGTGATAGTCAT

GCATATGATATTTCCTTTACACTTGAGGATTTAATTGATTTTGATAAGCCAAATAACATAGATGGAAACTTGCAAGAAAA

TGTTGTTTGTATTGATGGTAACACAGCAAGAAACTCTAGCATTGCATCAGACAAGAGTGATGGATATCTGGAACTAAATG

ATGTTTATGCAAATCCAAATGGACATTGCACCAACAACACTTTAGACGATTGCATGAGATCAAACCATGCAAATAATGCC

AACACCTCTCTAAAAATAGAAGATGCCGAGATCTTCAACAATTCCAATGTGACAACAGCTACCGAAGGGGTACATAGTGT

GCAGCCATATGATTATCTTAATCCTGCAGATGGTAATTACCTTGAAGACGATACCATGTTTTACGATGCACCTAGCAACG

ATGTTCAGTTTACAGAAGATACTTATATGAACATAAAAGAATACAATCCTACCGTTACTGATTTTGATTTGGTGGATGAT

TTGATGGCTTTTTTCGATGCGACGGAACAAAATGTTGATCCTTTAAGTGCAGATCAATCAGACATTGACAATCCTACAGT

CAAGTCAGAGGTGGAGGGCAGCAACTATCCTACAGGAAAAGACTGCCTACAGACTCCTGGAGCAAATATGGGAATGGGTG

CACCATCGACAGAATTCATAGATGCTGCCAATCCATTGGAAAAGACTAACGTGGAAGCAAATGACGGTTGGAACAAGTCA

TTTAGAAAGCATGTTGTGGATATGCTGGGCTCGATTTCTGCACCACCTGCAATGGCTGCTGCTGATTCGTGTAAAGGCAA

AGGTATTCAGATATCGAGTAGTGGCAGCAGCTCATCTATTCGTGTTTCTGCAGGTATGATCCAGATACGTGGTATTTCGG

TCACTGGACATCCAGAAAAGTGGACCCTATCGAAGGATGGAGAAATAAACTTCATTTATACCTATGCAAATGGCGCAGTT

TGCAAATTGGCAAGCCGTCAACCACCACCTGCCACTGTTTTTGGAGCGTTGCTTCGAAGTGGAATATTTCTTTTTGGCTT

CTCTGTGCTCGTTCTTTCCATAACCATGAAGCTAGGAGTTGGTGTTTATAGGAAATAACTAAGTTTCCAGAGACCCAAAA

AAAAAAACCTCGTTGCTTATGCTTTGATCATAGATTTCAGAGGTTGAAAGACAAAAACAATAATAATGTTATGTTTTGCA

TAAATTTGTTGGGTGCTAGGAATATGCACCCGTTCTTCCGACTATTACTGTTGACTCGAACTAATTTATTGTGCTAATAT

CCTGCCTATTATACGGATCATCTGTGCAAAATTTGGTATCTTCAAAATATTTG

>CepNAC13

TTGTTCTCTATATAACTGATTTATCTTTTCCACTTTTGTTTCTATTTCCTTCTCTGTTTCTCTCAACTGATCTCTCTCTT

TTGAAATACAAGAGTTTCAAATATGAGCTATTTGAGCATGATGGAGTCTACACTGCCTCCGGGTTTCAGGTTTCATCCGA

GAGACGAAGAGCTTGTATGTGATTATTTAGCTAAGAAAATCAATGGTTCAGATGGTACTAGTCATGGAGGTTTGTATGAT

GAGTGTCCTGCGTTGATTTGTGTGGATTTGAACAAGTGTGAGCCATGGGATCTTCCTGGGGATGCTTGTGTTGGAGGAAA

AGAATGGTACTTCTACAGCCAACGTGACCGAAAGTATGCGACGGGCCAACGCACAAATAGAGCCACCTTAACTGGATACT

GGAAAGCCACTGGAAAAGACAAGCACATTACTCAGAATGGTGTTCTTGTAGGCATGAGAAAGACGCTGGTCTTCTACCAA

GGAAGAGCGCCAAAAGGTACAAAAACCGACTGGGTCATGCACGAGTTTAGAATGGAGTCTTTTGATCATGCCTCAAAGTT

TCCTCAACCTAAGGAAGACTGGGTTCTATGCAGAGTATTCTACAAAGATAGAGGGATGGCTCCGACACCTACTTATAGCA

TGACGAACGCTATGAATTATGATGATTCAATGCTTGTGGAAAGGTACATAACGTTCGACGGTTATGATCAAGTGCCCTGC

TTCTCCAGTAGTTCGCTGCAGAACCCGACGGCTGCAGTTGAAAGAAGTGCGCCGGCTTATCGCGAGCAAGGTGGAATGGA

ATGCTTTGAACAGCTGAATCTGAATCAAGTGGACGACAACAGTGCGATTGCAAGTGTTTTGAATCAACTGAATAGAGTGG

ATGGGAAAACAAGGAGAGATGTGTGTATTGGTGGCTTGGGAGAAGGGAATTTAGGCTTAATGATGTTGTCATAATAGTAA

TGAAATGAAATAAAGATGTTAAGAGTAATTAGGGGGGTGTGTATATCTGTATTAAAATATTAGATGATTATTCTTTCTGT

CAATTTGCAAACATATGCTGGTTCATTATATGCCAATGCAATTGGTGTATGCTTCTGAAGATAACATTTTTTTCTTTTTT

CTTTTTTGTT

>CepNAC14

TCTCTCTTCTCACCTTACCCCCCTCAACAATTCGTAGTTTGCACACCATGACCAATCAAGCTCCCCTTCTCCCTCCTGGC

TTTCGCTTCCACCCTACCGACGAAGAACTCATTCTCTTCTACCTCAAAAATCGAGCAGAGGCCACTCCCTGTCCCGTCTC

TATAATCGCTGAAGTCGATATTTACAAATTCGACCCTTGGAATCTTCCAGATAAAGCTATGTTCGGAGACAAAGAGTGGT

ACTTCTTCACACCAAGAGATAGGAAGTATCCCAACGGAGTCAGACCAAACCGGGCAGCGGGTTCCGGGTATTGGAAGGCT

ACCGGAACTGATAAGCCCATCGTGACCACCATCGGAAATGAGAACATCGGTGTGAAGAAAGCTTTGGTGTTTTACATAGG

AAAACCTCCAAAAGGTGTGAAGACTAACTGGATCATGCATGAATATCGCCTTTCTGAAAGCAATATAAAGAGCAGCAACA

ATATTGCTAGATCCACAAGGATCAGAAATTCTTCCATGAGGGTAAGACAGTAAATCTAATGTGAAGCAGTTTCTTATGCT

ATATGATCATAGAGATAGTACTAATATACGTTCCTTTGTTTACATGTAGTTGGATGACTGGGTGCTATGTAGAATTTATC

AGAAGGGAGGTCATCTGAAAGAAGACTCGGTCGAGAATGAAAGTTCATTTGAAGATAACAAGCTCGAGATTCAAAGATCG

TTTTCTTTGTCGGAGCTTCTGAACGAAGCAGACTTTTCGCTGTTGTCGAGATTTATAGAAACTCCGGTGGATACAGGAAT

GTCTGGTGCAAACATGAATGTAACTACGTATGCAAAAGCAGAGCCTTCTGTTTTTGTTAACGGTTTGAAGAGGCAGAGAG

CCGAAGAAGGAGAAAACTCAATGTATCCGTTGAAGAAGAATATCGGAAATAGTTCATGGGATTATATGAACTTTGGCGAT

CAGTATGACAGTCAAACGTTTTATGATCAGCAGTTGTTGATGAACTCTCAGTTTGGGATGCAGTAGATAGAAGAAATAAG

TTTGTTTCCTATTTGATCAAGTAATTTTTGTATAGATGATTGTGTGATTTAGATAACTATGTGTCGAAAAATGGCACTAC

GTACATGAGTCATGGACTGGACAATTTTAGTGTTTGTATAATTTTGGTGACTAATGTTTATGATTAAAATGTCATTTAGC

A

>CepNAC15

AAAAGCTCTTAGCGCGGGCGCTTGTTCAGGAAAATGGAAGAAATACCGTACGATAAGCTGCCACTAGGGTTTCGTTTTCG

ACCGACCGACGAGCAGCTGATCAATCACTATCTCAAACGGAAGATCAATGGAACTATCCACCCGTCGGAATCTTGGGTGA

TTCCAGAAATCGACGTGTGCAGGACTGAGCCGTGGGATCTTCCGAGCAAATGGAGGGTAAAGTCGTCCGATTTGGAGTGG

TTCTTCTTTGCCGCGGTTGACCGGAAGTATCCTTCGAGGAATCGATCAAATCGGGCAACGGAGAATGGCTACTGGAAGGC

GACAGGCAAAGACCGGTTGATCAAGTCGAAGAAAGTTTTGGTGGGGATGAAGAAGACTTTGGTTTTTCATATAGGAAGAG

CGCCGAGAGGAACTGGAAGTTGTTGGATTATGCATGAATACAGAGTTGTTAATCCAGACGGGACTGATGTTGTGGGTGAT

GAGGGCGCTTTTGTTCTTTGCCGCCTTTTCAAAAAGGATAAAGATATCTTGAATGTAACACCATTGAGCCAAGCATTGCC

AAACTCAGATCTGTCAGATGATCCCCATACAGTCAATCCTGAAAATTCCTACGGACAAATAACAGAATATAATGTGGCTA

ATAACGAAGTGCTACCTTATTTGGTACCTACTACTATTAAACCAGAACAAAGTCACAGTAATAGTGATATTACGGCAGAT

GTTGGTACCCATCAAGAGCTTAAAATTAAGCAAGAGGAAGATTCTCTGTTTGACGGCCTTAAAGATTTTCTAGGCTCAGA

GTATGATCAGATTTTATCTCCTGAAGCTCCCGAAAATTACACAATTAATCCAAAAATGTTTGAGCAACAGCTTTGTGTCT

TATCTACTTCGGATATGGCTGGTGCTATTAGCAGGGATTCTGACGACTTAAAACCAATGTGGCGTTTACACAACAAAATG

GTTTATAAAGGAGCAAGTAAAAGGAGTCAGGGAGTACCGTCTGAAATGCATGAACCTATTGAGTGGGAAACTCATTTTAC

ATGTGACGATGAGAGCTTAAAAGCTTCTAGCAGCTATGAAACTGAAATAAATATCCGGCACAGAATGCCTGAAAGTTCAA

ATAGTTCAATGAACTACCCAAGTCCTCAAGGGACGGCTGAGCGGCGGATTAAACTTGAAAGAGTGAGACGTAGAAAACCG

AAGAAGCAAATTGAGATTGAACCACCAATGGCTACTTCAGTTGTCAGTGATCAAAGTTCTTTCTCCTCAAATAGTGATGG

TGATATCGATACAAATGACTTAAAAGTCAAAAGCCCGACTTCTGGATATTCGGTTCCTATGCATAGGATTGCTGTTGCAG

TTCTTGTCATTGTGATGATGCTTTGTGCTGGAACAATGTGGCTCTTGACCTCTTAAATTTTTTGTGTATGATATTGTAGA

TGGTAAGGTTTTGTTAACAACTAATTAGGATGAAAGTGAGAGATTTTAGATTTATGAGTACGTATTGAGGGGTACTGTTT

CAGTTGAACTTTTGAATAGCACTAGTAGTTGACTACTGAAGTGACATATGGATGGTATCTACTGCTTGCTTGGTAGTAGT

GTTTTTACGTACATCATAGAATGCTATTTTTGCTATGTAGTAGTGTGTATATGAAGTTTTGGTTTTCTGTGAAAAAGATT

GTTTTAGGG

>CepNAC16

CTTCGGTTTTCCAACTTTCAATTTCAAAGATTTACAATTCCTTCATACACAAGTCACCACTTGTAAATCGTCAAATCTTA

AGTAGAAAGAAGTAGATTTACAGAATGAACGGTGGAGATTTACAGTTGCCTCCTGGATTCAGATTCCATCCAACGGATGA

GGAGCTCGTCATGCACTATCTATGCCGAAAATGCACAGGGTTGTCTCTAGCCGTCCCAATTATAGCCGAAATCGATCTTT

ACAAATACGATCCATGGCAGCTCCCTGGTAACTTTTCGTTTCGTTTTCAGCCCCTATAAAAAATTTAAAATAAATTACTC

CAAGTGCTTATTTGCATAATCGCTTTCTTCAGGTCTAGCTTCGTACGGGGAGAAAGAGTGGTACTTCTTTTCACCTAGGG

ACCGGAAGTATCCAAACGGGTCAAGGCCGAACCGGGCTGCCGGGTCGGGTTATTGGAAAGCAACGGGTGCGGATAAGCCC

GTTGGGACACCCAAACCCGTTGCGATCAAGAAAGCACTAGTGTTTTACTCTGGTAAAGCACCAAAAGGTGATAAAACCAA

CTGGATCATGCATGAATACAGACTTGCCAACGTGGATCGTTCCGCTAGAAAAAAGAACAACTTGAGATTGGATGATTGGG

TACTGTGCCGCATCTACAAAAAGAAAGGAGCAGAAAAACCAAACGGGAACGAGCAGAGAGCGGCGTATGATTCGAGGATG

ATTCAGCAAGTACCGGAATTCGAGCAGAAACCCGAAGTCATTCAGCACGTGACGCCAACTTTCTCGCACGTGCAGCAAAA

TAACGGCTACGGGAAAAACGATTGGTTCAACTTCGACGCGTCCGAATCGATGCCGCGTCTGCACGCGGACTCGAGCTGCT

CGGAGCACGTGCTGTCGTCGGATTTCACGTGCGACAGGGAGGTGCAGAGCCAGCTGAAGTGGGGGGAGCTGGAAAGAGCC

CTTGCCGTTCCGTTCAATAACGTGAATGCCACCAGCTCGTTTCCGACGTTGGATAATGGGCCGTTAACGGATCCGTTGCA

GGACATATTTATGTATTTGCAAAAGCCATTTTAAGATTGGAGATCTGATATGTACAAGCGACGTGCTGGCAGATGCTGAG

ACGTTGATTGAGATGAAGTATGAAAGTAAAGACGGAGAGAGAGATATGTTTGTATAGATTGTAGTAGTGGGCTTGTTTGT

TGATTTCTAGTCGATGAAACCAAAGGACAAATCAATTGGTTATAGTTCAAAAAAATATTTTGGCATGTGGAAATGTTAAC

GAAGGATATGATAAATGGAAATGTTCTTTTGACGGCAAATAAGCGTGTCAATGTTGGTTTTAAAGTTACTATGAATGCTA

AAGGTGAATCCTTGCATGCTGCTTTTCAATTTGCATGTTAATCCAATTTTGTTAGAAAATATTATTTATAGGATTTGGAA

AGTGATATGTTTGGATTATAGAAATACTTGGTCGTGGTTATGTAAACGCTATTTTGATGTGTGATTTGAGATATCAAAAG

ATGATCAAGTAGCATGTCATTCTTATCTTTTGGCCCGAAAGCATCAAAA

>CepNAC17

TGAAGGCGCTTTACAACTAAACAAGGAGAACTTCATTCAATTCAAACTCAGGATTTGAATCATCTCCTTTTGTACACCTC

AATTCAATACTAATGGTTGTAGTGCCTCTTTTCACAACAGTGGATCAGCTTGACCTGGAGGTGTCAAAGTTGCTAAAGAT

AAACAATCAGCTATTGAGGCGCTAAATGCACGAGATGACAACTTCAATTCCTCGAATAGCATCAACTAGCAGTAGCCTGA

CAATACCAAGAGATTGAGTGTAAAATGAAGTAAGATGTTGATGATCTCCTACCGATGATTATCAATTTATGCATGTCGAA

CAGAGGTCAACGTCGTGGGTTTAAGTTTCATCTTAAAGAACCGTTGAAGGAAGGAAGCTCAGAGTGAAATAGCTTCCGAT

TCGGACTAGCAATGGGAGGGTGGAAAGGTCTATAGCCTGTAGCTTTGGCTTTTAACTCTAATCAATAGAACCTGTAGACT

TTTCATTGCCTAATGAAGTTGAGCAACAAATGACAACAGGGATTATTTGCATTATGAAAGCTGGTTCTACAAGCGAAGAT

AAGTAACAAAATTAACTGTATGACATGTAGCATACATCAGTGAAATGTAAATTCTCAGTAAATTTTCAGCTTAAGATGTA

TAAATTTCATCTACATTTCCTCATGCAGTAATTTGCTGTTAAAAGAAACGTGAGAGCTTATTCAGGAAATATGCCAAATT

TGCATTTTAATTGCATGCTGTGACGGATTCAATGGCCGGTAAAATCAAATTAGTGGTGTTAAGGGCAAAAGCTATGGCCG

TGTTGTCCTTTCCTCTACTTCTCTTATATTGCCATTGGAACATCATACTGGAAAACAAAATCAAATTCACTGCCAGTGTT

TGCTTGACTCGAATCAGGTGGATATAAAATCATATTCAACAAGATTTGGCAGAAATGGAATGTTGTGTCCCACCAGGATT

TAGGTTCCATCCAACTGAAGAAGAGCTCGTTGGATATTACCTTACCCGAAAAGTCGCATCCCAAAAAATTGATCTTGGTG

TCATCAATGACATTGACTTGTATAGGATTGAGCCATGGGATCTGCAAGAGAGATGCAAGCTTGGTTATGAGCAACAAAGT

GAGTGGTACTTCTTTAGCCACAAGGACAAGAAGTACCCGAGTGGAACTCGAACTAATAGAGCAACCAATGCTGGATTTTG

GAAGGCAACTGGGAGAGACAAGGAAGTGCTTTCAAAATCTAGAGTCATCGGCCTGAGGAAGACCCTTGTATTTTATGAAG

GCCGTGCCCCAAATGGAAGGAAAAGTGATTGGATCATGCATGAATATCGTCTTCAATCTGAACATGGCCCTTCCCAGGAA

GAAGGATGGGTGGTATGTAGGGCATTCAAAAAGCCAATTCCGAACCCTAGGCCATCATACTACAATAGTTACAGTCCTTA

CAGCGTTAGAGAACAAAACCAATATGAACAACTCAATGACCTTATCACCCGACCTACTCAAAATATCATAGAATCAAATC

AAGTACCTAATCATATCTTCGATCAACCACTCGGATCGATGGTCGTAAGCAAACTCAATGAGCCTAAAGTGATCACTAGT

CGAGGCATCTTCGAGTACATTCCACAAGTTGATAGTCCTAGTATATCCACGACGAATATTGCTGCTCGTGATCAATGTTG

CAAAAGAGATGATTCAAAAGGGCAATTTGTTGATTGGAAAGCACTTGATAATCTTCTTGCTTCACAACTAAATGAGTCAA

CTTCTTGTTCTAACTCGAAATTTCCAATGGTCAGTCTTTTGGATCAATATGATATGAATGATCCTAGCCATGAAGATGAG

TTTCTTGCATCATTTGACGGTTAGAAGAATTTCAGAAGAAACATAGATGTTCATAATGCAAGTTTAAGGGACATCTTGTA

AAGCATTGCATGCAGAAGTCATGATGATATAATAATTATATTATATATATAGTTTGTGATTAAGAAATTACTAATTGGAG

GGGTAATTTTTAATTGTGTTATATTAA

>CepNAC18

TTAATATTAAAAATTGATGCTAGCAATTTTTCTACAAACGCACAAATTATACTAGCCTCAATTTTTGATTTCTTTGTGTG

TATAAATGGCAACAATGAGCCTACCTCCTGGTTTCAGGTTTCATCCTACGGATGAAGAGCTTGTTGCATATTATCTTAAA

AGGAAGATCAACAGGAGAAAAATTGAGTTGGAAGTAATTCAAGAAGTAGACCTCTATAAGTGTGAGCCATGGGAATTACC

AGAAAAATCATTCTTGCCAAGTAAAGATTTAGAGTGGTTCTTCTTCAGTCCTAGAGACCGAAAGTACCCCAATGGGTCAA

GAACAAACAGGGCAACACAAGCTGGGTATTGGAAGGCTACCGGTAAGGATAGAAAAGTGTATTCTCAAAAAATAAGCATT

GGTACAAAGAAAACACTAGTTTACTACAGAGGAAGAGCACCCCACGGCTCAAGAACGGATTGGATAATGCATGAGTATCA

ACTTGATGAAAGCGAGTGTGAAACCCCCAGCTCCGGTTTACAGGATGTGTATGCTTTGTGCCGCGTATGTAAGAGAAATG

CACCAGGGCCTGTGATCATAGCAGAAAAATCAAACAGCTATTCATTACAGTATGAATCAAACTCCTCGCGGATAATCCAA

AAACCTATGAATTGTGTAGAAGGAAAGTGGATGCAGTTCCTAGACGAAGATGCATTTGATGCTAATGTTTCTCCTTTTCA

AGATAGTTCCAGTTTTCCTTATGTGCCCTCTGAGGTGGATGTGCTTGC

>CepNAC19

TTGAGTACAGATTACGCCACAAAGAGGTCTTCCTCTTCTTCCCGCAACCTCACTATATAAAACTCACCTCACTCCTCTAT

CTCATTAAAAACAACTCTCATTTCTCTCTTTCCCTTACATTTCTCATTTAAATAATACTCATCTATAAGAATGGAACATG

GGCTATTCCATTCTTAACTCTCTACAAAGTCAAAGAAAGTAACTATTTTTTTTATACTTAGAGATATTAAAATCAGCATA

TCATATCCAATGGAGAAACAAAACCCAAGTTCAATTCCAAGTATTGTGAGGCATAACAATCACAATGGAGTCGTAAAACT

ACCCCCTGGTTTCAGGTTTCATCCAACGGATGAAGAGCTTGTGCTTCAGTATCTGAGAAGAAAACTGTTTTCTTATCCAT

TGCCTGCTTCCATCATTCCTGAAATCGACCTCGCCCGACATAATCCTTGGGATCTTCCAGGTGGATGCGAACAAGAGAGG

TACTTCTTTGCACTACGAGAAGCGAAGTATGCAAATGGCAATCGTACTAATCGTGCTACTGAGTGTGGATACTGGAAGGC

TACGGGAAAAGATAGGGTAGTCGTAGCGACTAAGTGTGGAAGAGCGGTCGGGTTGAAAAAAACATTGGTGTTTTATCATG

GAAAAGCTCCGATAGGGCAGAAAACTGATTGGATAATGCATGAGTATCGACTAAACATTTCGAACCCTCGATCATGCGGT

TTATCCCATATGAAAAACTCAACTCTAAGTGATGCGATTGAGGTAAAGGATTGGGTACTATGCCGCATTTTCAAGAAGAA

AAGAAGCGCAAAGGCAAGTGTCGAACAAGAAGAGCATCGTTTCATCGATTTCATGGGCCGAGGAATGAATGGAAGACGCT

CTTCCTTGCCCGCTCATTCGGATTCAAGTTGTGTCACGGATCACGCAAGTGATGAATCAAAGTATGGAGAGGAAATTACT

TCATCATCATATCCGTGAAGATGAGATAGTAAAATATTTTAATTGCACTAGCACCACTTTAATTAGTGAGAAGCAATAAC

CTTTGAGCCTTTTTCTTTTTTCTTTTTAACTCTTAGGAACTCTGTATAACTTTGTGCTTTCTCTTTTCCCATTGTAATTT

CTTGATGCAAAAGAGAGCCTTTAGACATATATACAATTAAAGGTCCTCTCCCAATGTTTCAAATTGTTAAATCTTTGTTC

CTACAATTATGTGATCTCTGGGCTCTAGCAATCAGTTCATTTCGTGTGATAATATATTATTGAGAGTTTATGGAAAATTT

GCAAAATTTAAAACGTGTAATGTAACACTGTATGATGACATTGGATGCTCCAAAGGTGGTTCCATTATGTAATCATGATA

TACAAGCAAAAAAAGGCTCGTGTATTATTTGTCAATAAGAAGTAGACTTTTCATCAA

>CepNAC20

GCATATATTCTAAAATGAATCTTTATCTGTCATTCTAGAAAGGAAGTTCGAAGATATATGTTATATTTATACATTAAGTA

GAATATATATGTGGTGATGAGAAAAAATTATATGGATTCAATTAGTAGCTCCGATCTTATTGAAACAAAGCTCGAAGAAC

ATCGAATGAGCGGATCCAAGCAGTGCCCAAGCTGCGGTCACAAGCTTGAATTCAATAAGCCTGATTGGGTTGGGCTACCA

GCAGGAGTCAAATTTGACCCGACCGATCAAGAACTAATCGAACATCTTGAAGCAAAAGTTTCAGAAGAAGAAATGAGAGT

TTCACATCCTTTAATTGATGAATTTATTCCCACAATTGAAGGTGAAGATGGTATATGTTACACTCACCCTGAGAAACTCC

CAGGTGTGACAAGAGATGGATTAAGCAAGCATTTTTTCCACAGACCATCAAAAGCATATACAACCGGGACACGAAAAAGA

AGAAAAATACAAACAGAATGTGATTTGCAAAGTGGAGAAACTAGATGGCATAAAACAGGCAAAACTAGACCAGTTTTGGT

AAATGGGAAGCAAAAAGGGTGCAAGAAAATATTAGTGCTATACACAAATTTCGGTAAACATAGAAAACCCGAAAAGACGA

ATTGGGTAATGCATCAATATCATTTAGGAGAATTAGAAGAAGAAAAGGAAGGAGAGCTTGTTGTTTCAAAAATATTTTAT

CAAACCCAACCAAGGCAATCAAGTTTGCCCACTGAAAAGAGTACCGGAAGTTGTTCTACTTCTTCAAAAGAGGTTAATAT

CACCCAAAGTGATCGTCATGGGTATAACTGTAATTTGGAGCAGCTGCAACACATGAAGAACATTGATAGTAATTTCAGCT

TTGCTGCATTCAGAAAACCATTTGATGAGGTTGGCTTGAGAGAAGTTCCGACACAAGCAAGAGATCAAGCAACAGTAGCA

TCTATAGCATGTGACAAAGAACATGAAATCAGAGTGACTTCGGCGTACCATATAAGTCGACCAACAAACCCGATGCCAAC

GACCATACAAAATCCGCTACATCAAGGCTCGGTTGTTCTTGATCATCATGATCCGTACAATTTATCGACCATGCTTTTAC

ATCAAACAAACAAATTTCAGCAACATCAACAGCAGCAGCCACAGCAGCAGCCGCCTCCACCAAAGTTAGTAGAGAGATCA

TCCGCTGGCTTGGAAGAATTGGTTAAGGGCTGCACTTCTGCTGGAAATAGAGGGGAACCATCAATTCCAGTAAATACTCA

GGAGACAGACTGGCTAAAGTATTCATCATACTGGACCGACAATCCTGATCATCACTGACATAAAGAAATCAACGCAAGAT

ACATATATTAATGGATAAAAATATCTAATTTATACCAAGTAGAGTTTATTCATGGCAGCTTGACAAAATTTCACTCTTCC

GAGTTCATGAAAAATATGGTCGTGTACTGTGTTCTACCATAGAAAACATTTTATAGTATGCATACTCATAACTGATACAG

AAAAAAGAAAAAGAAAATCTCTTAAAAACATCATCATCTGCACTTAGAGTTGGAGTGTAATAATGAAAAGGGAAAAGGCA

GCAAAACTAACTGTAGAATAGGTAAGTTGCTTGGAAATTACGAAGAGCTAATAAGTGGGTAAAAAAGAGAGAATGGGAAA

AAGAAACAAAGGCAAAGAGCAAAGAGGATAATGCATCAAAATCAGTGCTGTTATCATTACTATATCATCTTCTTAATTGA

TTGCCTTTATTTGTCAGTTTATTATATAATTTAAAGATTTCCTTTTCTTTTCTTTGCGTTTATCATAATGTTGTATATTT

CTTTTTTCAATGTTTAAAGTTATGTAAAAGGAACGGGA

>CepNAC21

CGATCCATTCACTATCGACCCTCAACTCTCTTCAGATAATCATTGGTACATGTACAAGTCGGTAGTTTCAGACATTTACG

GTAAAGAAAGCGTCATCAAAGGAAAAAATGGATATTGGGAATTTATGAATGACATCAATATACCTACCAGTACTGCAATT

GGTATGAAAATTACTAGAGAATTTTACTTGGGTAATGTAACTTTTGGAGATAAAACCGCCTTTAGGATGCATGAGTATCA

TGTCAAACTTGAGAAGAACAGGTTCAAGGAAAACAGTTTGTCTAGAATATTCTTAGAAATTGAAGTGAAAAAAGAAGAAA

AAAGCAACATCGATGGTGAGAACATCGAATCAAAGTTGCCATGTTTGGTAAACCAGCAAGAAAAAAACTCTTCTTTGAAG

CAATGCCAAAATGACCTTGAGGTGAGTGAAGAAAATGAAGGGCAACCAATATTACCAAGCGGTCTGATGAACGAGGAAGC

TGGGTTTGAAGGTTTCATCGAGTTCAATGATTTTGGTGCAGAAGATGGAAGCTCATCGTTTTCGGTTAATACAAGTAGCA

GTGTGAAGTCAATGACTTCTGATGAGCACTTTGAATCGGAAATTGTGGGATTTGATCCAGAAGCTGTGTTGAGTGATATA

GCTAATGATGTAAACGATGTGAATGCATCAGTTGG

>CepNAC22

CTAAATAATAATAATTAATATCTCCACCGTTTCAGTTTTTAAAGATCTTCAGAATATCGACTTTATACTAGAGATATTTT

GGCTGTACATATTATTCTCAGTTTCTCTCTTTCTTTTGACATGGGTTTAAGGGATATTGAATCTACTCTTCCACCAGGGT

TCAGATTTTCTCCCAGCGATGAGGAGCTAGTTTGCCATTATTTATTAAAAAAGGTGACAAATGTAAGGGTTTATGAAGGA

ACAATGGTGGAGGTGGATTTGCATACTTGTGAGCCTTGGGAGTTACCAGAGGTTGCTAAGCTGGGCGATAATGAGTGGTA

TTTCTTCAGCTTCCGGGATAGAAAGTATGCAACTGGCTCTCGAGCAAATAGGGCAACAAAATCTGGCTATTGGAAGGCTA

CTGGAAAAGACCGTCCTATATACAACCAGAAAACACATGGAGTTATGGGCATGAGAAAAACCCTGGTTTTCTACCATGGA

AGAGCTCCAAATGGGATAAAAACTGCGTGGGTCATGCATGAGTTTCGGATGGAAAATCCACATATGCCCCCTAAGGAGGA

CTGGGTCCTGTGCAGAGTCTGTCAAAGAAGAAGAGAAGACATAGCAAGTTCTTCTTTGCAAATGACCAGTTCATCTCAGA

CCCCGAGACCAGTTGACCGGTCAATGCTACATGGATGCCCTGAGATGATGTGGGACCCACACCAAGATGGCCAAAGTACA

AATACTAACTTTGACAGTAATAATTTGGAAGCCTTGCAGTACAATATCTTTGATTTCACACAGGATATGGATATGAGTAT

GGGCTCAAAGGATATTGATGATGATTATGGACCATTTCTGGATTTAATTAGCTAATTATTAACTGAATGAAGAAATTTAG

AATAGCAGTAAGAACTAGTTGTTAGTGTCTCTGTGGATTGTTTGTGCTTCCCGTGCGAGTGTTTGATGTATTGCTATTTG

TAAACAAACAATATGGTTTAGCCATGTATCATTTGTGTGTGAAAGAATCATGATGTGCTTCGCTATATTTAATCATTTAC

>CepNAC23

ATCGAAGGAAGACCCTAGAAATAAAATAGCTAAAATCATTCAACACACAATGGGAAGAAGAACAAGAGACGCAGAAGCAG

AATTGAACCTGCCGCCAGGGTTCAGATTCCATCCTACCGACGAAGAACTCGTCGTCCATTACCTCTGCAAAAAAGTCACA

TTCCAGCGCCTACCCGTTCCGATAATCGCTGAAGTTGATCTGTACAAGTTCGATCCGTGGGAGCTTCCCGAGAAGGCATT

ATTTGGGCAGAAGGAATGGTACTTCTTCACTCCGAGGGATCGGAAGTACCCTAACGGATCCAGGCCGAATAGGGCTGCGG

GTCGTGGATACTGGAAAGCGACGGGTGCGGACAAACCCGTTTCGCCATCCGGATCCAAAAAGCCCTTGGGGATTAAGAAG

GCTTTGGTGTTTTACTCGGGTAAAGCGCCTAGAGGAATCAAGACTGACTGGATCATGCACGAGTACAGACTTGCTGATGC

TAATCGCACTCCTGCTAATAAAAAAGGCGGCAGTTTAAGAGTACGTATTTGGATCAGACCTGTGTTGTTTAGAAATTGTG

TGAAATTACTAAATAGTCTAAATTGTATTTTATTTTCTTCAGTTGGATGATTGGGTACTGTGCAGACTGTACAACAAGAA

GAACAACTGGGACATGACTCAGAATCTGAAAGAGCAATCTTCATCCTTCGGTGAAACAATGGACATGGATTCAGTAGATG

ATGATTTCAAGTCGGATATAGACAACGATTCATTTCAGGAGTTCAAAGATTTAAGAAAGCCTCAGATAGTGCAGAATCAA

AGCGTGATGGTGAATGAACAGCCGCAGCAGATGAAAAAGGAAGACAGTGATTGGTACATGGATTTGAATCTTGATGATTT

ACAGAGCACTTTTGGGGATTTTGGATCTTCAGCTACTGGACTTGACATGTCGAATTCGGATTATTTCTTGAATTTGCTTG

GGTCGCCGATGGGGAAGATGGGACAAGGTACTTTGCCCCCATTTTAATCGCAGAGTTTGATGTATAGGTAGATAAAATAT

ATGTGTATGTGAATAGCGTATGCAGAGAATGTAGTGTTGAAGAGATAGAAATATTGTAAGAGTTTTTCAACTTTCTTTTG

GCAAATTCAATCCATTTATTTATCCATGAAATGCGACTCGTCAGTAAACGCAGCATGGGGCAATTAACAATTCAGTGTGT

TCATGTACTGTGTTTTTAAAGGTTAATTATGAAATGTATTGCAAGTACAAAGGTTGGACATGATA

>CepNAC24

TAAAGCCCTATTACAGTACATTACATAGAGTGGTAATGTTGTTTTCCTCTTACCCAAAAAAGATTTCTTGTAATTATGGG

TTTTCTCTACTTCACTAGTGTTTCCTCTATATATAACTATCTGAGCTTTAATTTTAGTAAACACGGAACCAAACGCTGCT

TTACATCTCATTTTGTTTGTTCAAAACATATAAAATTTATCAGAAAAAAATACGATGGGACTAAGAGAAATCGAGTCACA

TCTACCACCTGGTTTCAGGTTTCATCCAAGTGATGAAGAACTAATATGCCATTATCTCTTCAAGAAGGTGAATAATGAGA

GAAATGATGAAGGAGCAATGGTCGAAGTGGACTTGCATACAAGCGAGCCTTGGGAACTTCCTGAAGTGGCGAAGGTAAGT

GAAAATGAATGGTATTTCTTCAGCTTCCTGGATCGTAAGTATGCAACTGGCATGCGTGCGAACCGAGCAACTAAATCTGG

GTACTGGAAAGCTACTGGTAAAGATCGAATGGTCTACGATCCAGTAACACACACGCTCATAGGGATGAGAAAGACTTTGG

TTTTCTATAGAGGAAGAGCTCCAAATGGAATCAAAACCGGCTTTGTCATGCATGAGTTTCGCCTTGGAAACAGCCATATG

CCTCCTAAGGAGAATTGGGTGCTTTGTAGAGTATGCCACAAAAGGAAAGGAGAAGTAGAGATGAACAATGAGTCATTCAA

CTCTCAACCTTTTATGGGAAAAGAAATCGAAAATTCAATGTTCGCAAATGGTGCACAGTCAAGTTCCTCATTTCCTGATA

TGAACAACTTAATGCAATGCAACTTTCTTGATTTCACACCTGAATATGCACAAGATTATGATTTGCCTTTTGATGTGGGG

ACTGAGGAAAATGCTATGCACTATGAAAATTTAGTAGATTTCAGCTATTAAGCTGACATGCAGATGTATAGTGAAATTGT

TCGATTTCGTTGATTTCAGGTGCAGTGTTTGCTTTTGTTATATGTAAATAGTGCTTTTGTGATGTATTTTAAATACGTCC

AAAAAAAAAAAATCGTATTTATGTTAGGGTGAGATTAATTTTGTTTTACGCTGTG

>CepNAC25

TTATGATTAAAAATGGTCCAGAAACGATGTCGTCTGTTGAATCGTTTGCGTGTGCAGGTTAAGCTACTATAATATGGATT

TAGTAAAATGATGGATTTCCTTTAAAAATTTGTATCACTCAAGAAGCACTACCTCAGAAAACGAAAGCTTACTGGACTGA

CAGACTCCTGTATGGAATAGGCTTGGACAAATATGAGATGTGCATAAGAGAAGCGTCACTTTAGAAAGTTCATACTAATA

AAGCAGAATTTCATGCGTATCGCTTACTTTTCACTTTTAATGGTGTAAGGTGCGAATAAGCTTCTTGACTAATAAACGGA

TTCATCAGTATCTCCCATAAATCTTGACTGTGAGCTTAAGCTAATGATGATATGGGATTAAGAGATGTGGAATCTGGGTT

ACCACCGGGCTTTAGATTTTTTCCTAGCGATGAAGAGTTAGTATGCCACTATCTTCATAAGAAAGTTGCTCGATTAGCAA

TGAAAGAAGAAACTATGGTGGAAATAGATTTACATACATGCGAGCCATGGGAACTGCCTGAGGTGGCCAAGCTAAGCGTG

AATGAGTGGTATTTCTTCAGTTTCAGAGACAGAAAGTATGCAACTGGATCACGTACAAACCGTGCAACAAAATCTGGGTA

TTGGAAGGCAACAGGAAAAGATCGAACAATCTACAATCCATCTGCAAGGAATGAGATTATTGGGATGAGAAAGACATTGG

TCTTCTATAGTGGAAGAGCTCCTAATGGCATCAAATCCAGTTGGGTAATGCATGAGTTTCGGTTAGAAAATTCTCATTCA

CTCCCTAAGGAAGATTGGGTACTCTGTAGAGTATTCAACAAACGAAAAGAGGAAGAGGGGTCAGCAATCACGAGTTCATC

ATCGTCCCCATTATTTTCAAGGGACTGCCCTATGGAAGGACGAATGTATAAGCATATTGAACCTTTGCTCTCTGGTGATC

ATAACAATAGTTATTTGTGTCCTACATACGAAAGCATAGGTGATTTCAATGATATGTCCAATTTGATGTCTTTCAATGTT

CCTCAAGCTGAGATAGAAGATTTTGGATTTCTTATGGACGTGGGCCTATAATAAGCGAGGTTCAGGCGATAAGCTTTGGT

AGCAGTATTGGCTGTAGCTCATGAATCAATGATTGAAGTGTGG

>CepNAC26

ATTTGATCCAAAGCTGCTAAACTCCCCCAAGAACTAAACAAAGTCCATAATTCAGTAAAAGGAAAATCATCAATTTTATG

ATCCAGGGTTCTGAATTCCGCACAATCTCTAATTAATCGGAAGATAGGTAAACATGACTGTTGTTCCCATCAAATCCTTG

CCGTTAGGGTTCCGATTTCATCCGACCGACGAAGAATTGGTGAATCATTACCTGAAGAGGAAGATTAATGGAAGGATTCG

ATCTGAAATGGAGGTTATCCCTGAAATCGATGTCTGCAAGTGCGAGCCCTGGGATCTTCCTGGTAGAGCTTTGATAGAAT

CCGATGATCCAGAATGGTTCTTTTTTTCACCAAAAGACCGAAAATATCCGAATGGACACAGGTCGAACCGTGCAACAGAG

GCAGGATACTGGAAAGCAACAGGAAAAGACCGATATATTCGATCAAAAGCTCCATCAGCAATTATCGGTATGAAGAAAAC

ACTAGTTTTCCATAGAGGTAGAGCTCCAAAAGGTGTTCGCACTAACTGGATTATGCACGAATATAGAACTACTGAACCGG

AGTTTGACTCAGGAGAGCAGGGAGGCTATGTTCTTTACCGTTTATTCAGAAAACCGGAAGAATCTGCTCCACTTTACAAT

GTTGATGAAATGGACAGAAGTGATTTGTCTATAACGCCATCCAAATCATCCCCAAGCGAAATACAAAATGAAGTAGATGT

ATTTGAAGAACTTCAAACTCCTGCAATGCTACAATGGAACCAAGTATCCCCTGTGTCCGATATACAAGAAAATACAAAAT

TTCAGGTTGCCGTCAATAAGTATGACAATAACAATTCCTTGAATCTAAAAGATGGATATAACAACAACCTTGAACAACGA

GAGTTGGTTGATCCTTTACTGGAAGCTTTGGGTCAAGTGGATTCGAATGGGATCCCTAATATCCCTTCTCCTCTGTGCAC

TTATGTTGACAACTCATTCCTCTGTAATGGCAATCAAGAATCAAACACCGGGCTGTTTCAAGCTAACTTCAATGAACCAG

ATCCAATGGATGATTTGTTGAACTCATTTTTTAGTAATGAAGTCGATTCCCCTAGTGGACATGAGGGAGTTGGTTATAGC

GATCCTGAATGGTTCTACATGGATGCTTCAAATGATTATTCAGAAAGAACATTCCCTGAAAACGCATTCCTCCAATTTCA

ATCAGCGCACGACGATTATTCCCTGGATTCTGGATCTGAATCTCTACATGATTTGTTCAACAACATGGATGAATCTAATG

CTCAGTTTAACTTGTCCAACCATGAAAATAATCTCGAGGAAAGTGGAAGTACTGGAATCAAAATCAGGTCAAGACAACCT

CGAAGTGTAATTCCAAATAATATGCCTGCGCAACAGGGTTTTGCAATAAGAAGAATCCGTTTGCAAGTTTCTACTGATGA

TGCTGAACGGAAGCTCAAGCTAGAAGATAATGAAGAGATTTGTGAAACTGAACAGGTTGAGAATAATGGGGAGGATGAAG

ATCACGGTGACATCAATGTCGCACCTTTAAGCCTAAAAGAATCTGAACCCAATTTGAGACTCAGAGCAAAAACCTCAGTT

GAGATCAACCTAAATGAGGAATGCAAATATTCAGTGAAGGAGAAAGAAGAAATAAAACGTCCAGTGACATTTAACTATGT

CATCTTTATGACGGTAATGATTATGATAGTCGTGCTGCTTTTGGTGGGGATGTTGATGTGGAAGGAAGAGATTTTGGGGT

TTGTGGATTTCTGAGTAGTGAAATCGATAGATAACAATGGTTGTATAATATTAAAATTAGATATCATGCTCTTATGTTAT

TTGTTGCAGAGCTTATTATGTTGTAATCTTAATTTGCTGACCAGAGGATACAAAAGATTTGTATGCTTAACAGTTTGCCG

TTTGTTTGTACACTTCGAGCTGTCTTGGTAGTGTATTTTGGATCAATGCTTATGCAGCTTTTGACATCTCAAGCATCGTT

TAATGTATTTAGTTTTTTAGTGGTTGTTACTGTGTATTGGATCATC

>CepNAC27

AAGATATTTCTTGTGAACATCCTTCTTCTTAAGTCGATCTTATATCACAAGCTTTAAGGTCTCTCGAGATCGAGATCGTG

ACCGAATAGTATACCTTTTTTACACATCTGGTATTCAAGACTTTAATAGGAAAAAAAAAGGTATACCTCCTTTTTAGCTT

TCAAAAGATTTTCAGAGTATCTATAGTGAACTTGTACTGCTCTCACTTTGTTTTTGCTCCACCTCTTTGTTTCTTGCTTT

TGATATGGGCTTAAGGGATATTGAATCTACGCTTCCACCGGGGTTTAGATTTTACCCGAGTGACGAGGAGTTAGTTTGCC

ATTACTTGTACAAAAAGGTGGCAAACGTTAAAGTTTATGAAGGAACATTGGTGGAGATAGATCTGCATAATCTCGAGCCT

TGGGAATTACCTGAGAGTAGCAAGCTGAGTGATAATGAGTGGTATTTCTTCAGTTTCCGTGATCGAAAATATGCAACTGG

CTCTCGAGCAAATAGGGCAACAAAATCTGGTTATTGGAAGGCAACTGGAAAAGATCGTGCTGTTTATAATCTGAAGACAC

GGAGGTTAGTGGGCATGAGAAAAACCCTAGTTTTCTACAGTGGAAGAGCTCCTAATGGATTAAAGACTGGGTGGGTCATG

CATGAATTCCGTATAGAAAATCCTCATACGCCTCCTAAGGAAGACTGGGTTTTGTGTAGAGTTTTTCAGCAAAGAAAAGG

AGAGGCAGGTTCTTCTCAATCGCAATCGGGCCATTCGTCTCCTGCAATGGCCGATGGTCAGTCTGTGAAAGATGAATGCT

TTCAGACAATGTGGGATCCACATCAAGAAGGTGTAAATACGACAGATAATTTGGAAGTACTGCACTACAATATCTTTGAT

TTCACACCAGAGTTGAGCACTACATACACAGCCGGTATGGATATGAGCATGGGCACCATGGACGTCAATGATTATGAACT

TTTATTGGATGGGAATTTGTTAAGTAACATGGGGGAGCTCAAGGATTTGATTTGGTAAATTTGATGAAACGATTTCTGAT

AGTAAGATCTAGTTTAATTTTTTTTTCTTCTTACTTGTTTGCGTTAGTCAAGACATAGAGAGATACAGTTTGTATTTGAT

GGTGCATGGTGGTTGTGGTGTGGAGCTTTGGTCTATTATTGTTTTATGTATATACAAGTAAAATGTATCCATGATCGAAA

CTTTGGTTAAAAAAAAA

>CepNAC28

GTCAGCATTTACCTCTTCAACTTCCTTCACCCTTCCTCGTATAAATACAAACTCAGCTTCCTTCACCATGCTCAATACAT

AAAATGAATAACAGTGAACAGCAAATTCCTCCAGGCTACCGATTCTTTCCTACCGAAGAAGAGCTCATCTCTTTCTACCT

TCATAACAAGCTCGCTAATGCAAGAGAAGAAGAAATAGAACAAGTCATTCCTACTGTAAACTTATACTATCTCGATCCAT

GGCAGCTTCTTCCCTTTGCTTCTGTACAAGAGTCGGAGCAGTGGTTTTTCTTCTGCCCTCAGCAAGAAAGGGAAGCACAT

GGAGGCAGGCCTGCACGGACCACGCCATCCGGGTACTGGAAAGCAACTGGCTCGCCTGGTTCGGTGTATTCGGGTGTGAA

TAAGCTTATAGGCGTGAAGAAAAGCATGGTTTTTTATGAGGGGAAGGCTCCTAGAGGCACCAAAACCAAATGGAAAATGA

ACGAGTACAGAGCTCTGGATGAAACTGTCGTTAATGGTGATTCTCCTAAGGTACTTGTTAGTACGTTTTAACAGTTAATT

AAAGATAAGATATGTATGGTAATGGTTTTTTTTCATATCTTTTTTCTTTCTTTTAACAGCTGAGGAGTGAATTTAGCTTG

TGTAGAGTGTACAAGAACTCGGGATGCATGAGAGCATTCGATCGTCGGCCATTAATGGATGCTGCGACAGTGCCTGAACA

GAGTCGTTTAGGTTCAGAAGATCGAAATGATGATAATAGTTCTCTTGATGCATCCTTGGAAGAATTGAATGAGGATATTA

AGTTGCTTCAAGAAACGGATTGGGAGGATTGGCTTTGAGTATATATTTCCCGCTTTAGGTTGGGATCAAATAGATTATTT

TAGTTGCACCTATTATTATAATAAGAACTAAATAAATTGTCGAGTTCTTGAATAAATTTGTAGGGTGGACACCATGTTAA

TGTGGCATCCTCTTTTATGCTTGTCAATTTATCATTGACTTCTGTATTATTATTTCTGATTTGATATCAATCCATGTGCT

CATTTCTCATTCATTTGAGTAAACATTTTTGGATTGGTCCTAAGATTTATTTATGTTCTTCTGTTAAGCTATTTTTTTAT

TAATATATATGTGTCTGAACGCG

>CepNAC29

CTTCGTTCTTTAAATAAAAATAGAATACTACTTGTTAGAATGAGCGGTGGAGATTTGAAGTTGCCGCCTGGATTCAGGTT

TCATCCGACGGACGAAGAGCTTGTGAAGCATTATTTGTGTAGAAGATGCACTGGGATGAATATTGCAGTTCCCATCGTCG

CTGATATTGATCTTTATAAGTTTAATCCGTGGGAATTACCCGAGAAGGCGTTGTACGGAGAAAAAGAGTGGTACTTTTTT

ACACCGAGGGATAGGAAGTACCCAAACGGATCTAGACCCAATCGGGCCGCCGGATCCGGTTACTGGAAAGCTACCGGGGC

GGATAAAGCTATTGGAGCTCCTAAACCAATAGGAATAAAGAAGGCATTGGTCTTTTATGCGGGTAAAGCGCCTAAAGGTG

AAAAAACCAATTGGATCATGCATGAGTATCGACTAGCGGATGTTAACCGATCCGTTAAGAAATTGAACAGCTTGAGGGTA

TGCTTTTAATTACACACTCTTTCTTCGCTTCTATGCTACGAAATTTCTATTTAGTGCAAAAATAATTATACTTCTGTAGT

ATTGTAATATTGCTTAATGTCCAATAAACTTTTCGGCTAGTAAAACAACGTGGCCATTAAGATCAATTTGGACCATACTA

GTCACTGCAAACATCATCTAATTCAGCTGCATATGACAAGTTGTTGCGCAAATATTCACGAAACATATTAAAATATACAT

CGATATAGAAAAATCTAAAAATGGCTGGGGCCATCATGCTATGAATTTTTTTGTCGTGAATGCAAATGAAACAGTTAATA

GTATTCATCAAGTAGTAATTTTCAATTATTAACGTGAAAGGCACATGATTCGTTCTTAATCAATTTATCATTGTACATGA

ACAGAAAAAGTCATTTTCTGATAATTACAGCGATTGTACTTGCTATCGTTTGACGACTGAACATGCTTCCCACTTGTTTA

TTTGCAGTTGGACGACTTTGTACTATGCAGAATATACAACAAAAAAGGCGGGCTGGAACGGCATATACCCGAAAACCCAA

ACCCGGCCCGAGGTTTACCGACCCATGTTAAACCAGAGCCCATTATTTTTCCGCACGTGCCAATGGACATGGTGCATTTT

GATGCAGCAGATTCAGTGCCGAGATTGCATACAGATTCCAGTTGCTCGGAGCACGTGATTTCCTCGTGTGAGCGTGAAGT

TGAAAGCCAGCCGAAATGGAATGAGTGGGAAAAAGCCCTTGATCTTCCATCATATAACGGTACTGCCACAGAGTTTTTCC

CGGATTTTGGGTCGGGCTCATTTGATCCGAAGTATAGAGACCCGATTCAGGATATTTTGATGTATTTGCAGAAGCCGTTT

TGAGATTTTTAGCTTTAACGGTGTTGAAACGGGTTTGCGATGGGGATGGAATAATATGTACATGATATTGGTTATAAAGT

ATAGAATGGAAAAAAACAATCATTCAACGTTTCCTCTTTATTTAAGATAGTATTTTATTTATGATAGTATTTTGTATCAA

AAGTTATAACAGCCATTAATCATGATCGAGTCGGGCATTCATTCTACAAACACTTGCACAAAATCAATGTACCTGCCTTC

GGCAATGTTAAATGCAAG

>CepNAC30

ACCTTCTTCCGCTGTTTTTTCCCCCTTAATGTCCAAAGCGTTGATCGTTGATCTATCCGTAGAATGGCAAGGGCTTGTTG

GCTTATCGATGGAAGGGGAATTGCAAGAAAAGTAAGAGACGCTTCTAGTTTTTCAAAGTGCCAAATCAAAGAGCTTGGAA

CCGAAGCACATCGTGAATGCCCTAACTGCAGTCATATTATTGATAATAGTGATGTTTCTTTAGAATGGCCAGGTTTTCCT

GCAGGTGTTAAGTTTGATCCAACGGACATCCAAATTCTCGAACATTTAGCAGGGAAAGTAGGTTTGCCTAATTCAAAATC

TCACATTCTCATTGATGAATTTATACCAACTCTTACAGAGGAGCAGGGAATATGCTATGCACACCCTGAAGACCTACCTG

GTGTGAAAAATGATGGAAGCAGCGTTCACTTCTTCTACAGAATATCCAATGCATATGCAACAGGCCAACGAAAACGTCGT

AAAATTGGTAATAAAGAATGCAGTAGTGCTTCGGAAGAAAAAGCGAGGTGGCATAAGACTGGGAAAACTAAACCTGTTAT

AGACAAGAATGGAAACCAAATAGGATGGAAAAAGATTATGGTTCTGCATCAGAGTTTGAAAAAGGGAAGTAAGCGGAAGA

GTAGTGCTGATAAAGCTAACTGGGTAATGCATCAGTATCATTTAGGCACAGAGGTAGAAGAAAAGAATGGAGAAATGGTC

GTTGCTAAAATATTTTTCCAGCAAAATAGCAAGCAGAACAATAAAGTTGATGCTGAACTTTCTCGAGAAGAGCCTATCAA

TGTTGCAAGTGGTCCAAGAACTCCAAAAATGGTTACACCTCTGCAGCCCCGTAAAGCAAAACAGTCACCCATGTCTGAAG

CTGAGTACAGGGAAGAGGTGATTCCACAATCAGTAGTTAACAAAAACGATGAAGGGAATTTCCTCTTGCTTGCAGGAGAA

TCACAAGCAGTCGATGAATTCAACCCAAAACCATTGGATGAACTTCTGTTCTGCCATGAAGTATTTGATAACTCTGCATA

CACAAATGACACATCTTTTGGCAACAATGAGCTGCTGAATACAGACGACTTCAACTCCTCCAACCCCTTTGATGATTTGA

ATAACATACCTTTAGGCACACCCCCTGATTTTAATCTTTCGGATATACAATTTGGTTCTCAGGACAGCATATCTAGTTGG

TTGAATCGAATGTAGTTATGTTGACGAGCTAAGCAGAGC

>CepNAC31

GGTTAATTACCGAATCAACGGAATACCTCACATAAGCTTAGACAGTAGCGTCTCTACACCTCATTTTCTGGAGGCGCTTC

TTGACGGCCTTTTCCTCATATTGGCTTGTTCAGCAGATGCCTTTTGACCTCACCAAGGAGTTTCCTCCGTGGTCACTCTG

GTGTGGCTTCAGGTTATATCATTGTGAACTGTGGCGTTTGTGCAGAGCAGTATATTTTCTTCAAGCCTGAGGAAAGACAA

TGAATTTGATTTCAGTCAAGCTACAAAAATAAATTTTATATTCCCTTTTCCAGAATTTTTTTTTGCTGTTCCCACTGATA

TATTTTTTCTCCCATCTGCTCAGGGAAAAGAAAAACAAAACAAAACAGTCTTGCTGTTAGTTTTAGTTATAATCCTAAAT

CAAAAACCTTTTCTTCCTAAAAGATACATTTTTTATTTTATTTTTTTCTCATGGTTGATAAATTGACGCTCCCCGGCTTT

CGATTTCACCCAACTGATGCTGAATTGGTATTATACTATTTGAAACGCAAGATTCAAGGAAGGCTTTCAAAAAGTCAGGC

TGTTATTTCAGAAGTTGACATTTATAAGTTTGCTCCTTGGGAGCTTCCAGAGAAATCTTGTCTTCAAAGCAAAGATCTTG

AATGGTACTTCTTCTGCCCTCGAAATAATAAGTATTCACATGGAAACAGGACAAACCGTGCTGCTACTGGTATTGGTTAC

TGGAAACCAACAGGTCAGGACCGCCCTGTCATCAACAAAAAGCAAACTGTAGGAAAGAGGAGAACCCTAGTTTTTCATAC

AGGCAAGTCACCAGATGGCACTAGGACCGATTGGGTCATGCACGAGTTTAGGATAGAAAATTCAGATGAATTAGTCAATG

CTGGATTCAAACAGGATGCCTATGTGCTGTGTAAAGTTTTTGAGAAAAGCGGCCAAGGCCCCAGACCTGGTGAACAGTAT

GGTGCTCCATTCAACGAAGAGGATTGGGATGATGAAGTTGATACGGTTATCAGCAATGATAATTCTTTACCTAGTGCTCT

TGTGATTGACTCTGAACTTTTACAATCTATAGCTTCACAAGCTGGTGAACTGAACAGTGTGCATCATGTTCAAAATTCTT

CTTTGATTCAACCTTCTAGCGTTGGAGCTGAAACATCCTTGCAAGTTCATTTTGAAGTCGGTGAGAGTTCTAGAGCCCAA

GAGTTATCTTCGATGCAATGTAATTTGCCGCCTTCCTTTTCTGATGGAGTTTCTCTTGGATCCCTTACTCAAGAAAATGC

TCATCTTGTATCGGAGATGCCAGGTTCAGCTCTTCCATTGGAGAATTCTATTCAATATGCAGACTTGGATTTTGACTTGG

GCGACCTCGGTGATCTTGACACCTCCATCCATCATCTAATGGAACCAGACGATGGCTTTTCTGTCCTTGAAGTTTTAAGC

TACGTCGATAATCAAGAGCTTCAACATTCTGCAGAGTATGCCAATGGAGATTATTTGACTGTCGCCGATATTGCTTCTGA

AAATCAAGCGAATGTCATGGAACAGCCGCCTCTTACCAACGTCCAAGATTCGCTAAACGTTACCTCTGGCGACGATTGCT

ATGACCACATTGTTAACTCTTTGTTGACAGATAATCCCCAGCATACGGAAATGTCTGATGTTTTCCTTCCGTGCATTGCA

TATCCTGATTGCCAATGCTGCAACAACTTATAGCATGATATTACCAAACTTGTTAAGAGAGCATCATCTGATTTTGCTTT

CAGTATAGTTTTACTTTTTTGGAATTCTTCTGGTTGGTGACAGTTGTGTATATTCTGATACAACAACCAGCCATAATTAT

TTTTCTCCCTAGTCTGATTTTGTGTGATCAAGTTATGAATACTGAATTATTCAATGTTGGCTTTTCTTAAGCATGGAACT

TTTTATCTGCTACGATTTACTTCCATTGTAATTCTGTATGCATTGTTAGCTGGATCAGTGTCAGTGCACCTTATGTAAAT

TGATATCCATGTGTTCTAAATGTTATTTTTCTGTTAT

>CepNAC32

CCTAACCGTCCTGGCCTTCAATTTTCTGCCTTTTCAATTCGTTTGAAAACCTTTGCTCTTTTACTCTTTCAAACATCTTG

AATCGTGCATTTATCAAATTAACAAATCAATAATAGGCGATTGATCGATTTATTATTTTCATCTATATCAAAAATTAATG

ACTATCCAATCATCATCGTTGTCTTCAACCCTAGCGCCAGGGTTCCGTTTCCACCCAACCGACGACGAACTCGTAAGCTA

CTACCTAAAACGCAAGAAATCCAACTCCCCTCTTCTTGTAGATGCGATTTCTTACATCGACCTCTACAAATACGAGCCGT

GGGATCTCCCATCTCTCTCCAAGCTCAAAACCCGCGATATGGAATACTACTTCTTCACCACTTTAGATCGAAAACATGAT

AGCCGAATTCGAACCAGCCGAAGGACTTTGGATGGGTATTGGAAGACTACAGGGAAGGATAGGGAGGTTTATGGTGTTGG

AAAGGTGGTTGTGGGGATGAAGAAGACTCTGGTTTATCATTCTGGGCGTGCGCCTACTGGTGCTCGGAGTAATTGGGTCA

TGCACGAGTACAGATTAAATTCTGATTCCTCTTCCACAGCAGATGATCTTCAGGTGAGTTAGATGAAACCTGTGGTGTTT

GGTTTGAGAATTTAGATGATATGTTTTATGCGGTTGAGTCGTGACATTTCGTTCAAATGGGGGAAGGAAACCCTTGTTTT

ATGCAGGATATTTCAGAAGAACGGTTCTGGGCCTCAAAATGGGGCGCAATATGGCGCACCTTTTCGGGAAGAGGAGTGGG

CTGAGGGTCAAGGCCTTGGTCCTAAAACAAATGAAGGCGAACTGAATGTCTTAATGCATGCCAATAATATAGAAGAGAAT

CAAGCTCTGGCTGATGGTCAGGGAAGCACAGATATTACTGCAAATTCGGAGGAAAATGTTGGAAGTCAGCCAAGAAATCC

AAGCAATCTTCTTGAATTAGATGAAGGTATCAATGTACCTGAACCACAGGTTGCCGACAGTAATATTAAAAAAATGTACA

CCTTTGAAGAACAAGTTGAAAATCACTCAGGCCAAAAGGATGGATATCTTGAACTAAAGGATTTTCACAATGATGACTCT

TTCGGTCAGATGGGAGAAAATTATAGGGATGAATCCACCGCAGTGCCGCAAGTTGATTACGCTAGTTTATACTTAGACAA

TGCAGCTGAAAATATGAATTCTAACGGATTGCGAACAATTGATCCTAGTCCTATTTCTTATGACTATCATTCTGCTCAGT

CAGTTGCTGTTGATGAAAATATATTCTTTGATGCATTCAGCAACAACTTCTCATTTACAGAAGACATTTTGGATGATATG

AATAATGCATCTGGATATGATATGGTGAATGACTTATTTGCTTATTTCGACGCCACAGAGAATAACTTACAGAACGATAT

GGAGATTTTCAATTCGACTACCACTGTGCAAGTCAATGTCAAATCAGAGGTTGATAGTGGTCATGTTAAAGAAAAAGGCT

CATCACATTCAGCTTCCAGTAGTGAGAATGAAGCATTGAATAAGGGCAACAGTGCAACAGCAGAAGTGCAATCGCGCATG

GGTTGGAAAAGTGCTGTTAAAAAGCAGGTTGTGAATATGTTGGGCTCAATCTCTGCACCGCCTGCTATGGCTGAAGAATG

CTCAACGGCGATCAAAGCGATTCCTGCACCGACTTCATCTATCCCTGCTACTTCTGGCATTATCAAAATTCAGAACATAT

TACTCGATGAAAGCATGGACTTCTGGTCTACGAAAAAGATGATTGAACGCTCAGAATTCGAGTTATCCTCCTATGAGCCA

GTTGTAAAGAGGAATGGGGCTTTTTCAACAGTGCTTAGATGTGGGTTCTACCTTTTCGGTGCATCTGTGCTGATCTTTTC

TGCATGTTTGAAAATTGCAATGTGTTTGCATAACAAGTAAATTTGCTTGGTGCATAATCATCTAGTAATAAATATGCCTT

AGAGGAAAATTTTTTTAATGTTTTTTTTTTTTTAATGAGGAGAATCTTCCTAAAAGCCAAATATGCTGAATTTTGTAATT

TTTATTACATTTAATGTTTACAGCCAAAGTTGTATTGATG

>CepNAC33

GGCATTTTTGTAAACTTGTGTTTTTTTTCTTTCTCCCTCTCTATTTCTAAATTATGATCTGAATTTTTCTTCTTTTATTG

TAGTATTCTAGTCAATAAGGCGAGTCACTCTTGCTTTCAAATTGTTAATCATGGAGAAAATCTTTTCTTTCAGCTAAATC

CATGACTTGGTTCAAATATCTCGTTCTTTCTTCCATAAAAGTTCAATTTTACTACTGATTCAGCAATCGGAAACTAGTTT

GAAGGCAAATTTAGATTTCTAAACGGGATGTGTCCTATTCCGCCTCTTCTGTCGGCCGATTCGTCGGATGAATTTGTTGT

GATGTTTCTGGATTCGAGGAGCGTTGATGATCCGATGCCATCTAATGTGATTACCGATGTCGATCCATTCACTATCGACC

CTCGACTCTCTTCAGGTGATAATTGGTTTATGCACAAGTCAATCATTTTGGACACTGTGGATGAGAGAAGTATGATTAAA

GGGAATAGTGGATGTTGGGAGTTTATCAATAACATCAGTATATCCATGCCTACCAGTACTCGTACAATTGGTAGGAAAAT

TACTAGAAAATTTCTCTTGGGCAATGGAACTTCTGGGGATAAAACCACATTTGGGATGCATGAATATTTAGTAGAACAAG

ACAAAAACAAATTCAAGGAAACAAGATTGTCTAGAATCTTCTCACAAATGGATGAGGTGAAAATTGAAGAAGAAAGCGGC

ACTGGGGGTGAGAACACAGAATCAAAGTTGCCATTTTTGGTAAACCAGCAAGAAAGAAACTCTTCTTTGAAGCAACCTGA

AAATGAATTTCAGGCTAGCAATGGAATTGAAGGGCAATTGGAATTACCAAACTATGTAATGAACGAGGAAGCTGGACATG

AAGATTTCATTGAATTGAAAGATTTATTTGGTCCGGAAGATGGAAACTCGTCCTTTTCGGGTAATACGAGCGTGAAGTCA

ATGACTTCGGATGAGAACTTTGATTCGGAAATCATGGGATTTGATCCAGAAGCCATGATAAGGGATATTGCTAATGATAT

ATACAATGCGAATGCATCAGTTAGCTCTGATCCAGCGGTCATCCGACCATCAATTTCTCTTGGTATCTTTACAAGAAACT

TATTCCATTTTGTAGTTGCATAAATTGCAGAGCTTTCCTTTTTCATTTGGTAATATCTGAAAACACTTCAGTTCCAGGTG

CGGTGAGAAAAGGTAAAAGTGAAGCGTCAACCTCCAGTTCATCTGAAAGCGGTGGAGATGGTACATCAGCAAAGCATTCT

GGAAATGGTACTTCTTATAAACGTGCAAAATCAAGGTCTGCCAGGCGAATGGCAAAGCTTGGTAAAAGATACTGCTGTTT

TATTTGATGAATCAGACACGATTTTTAGGTACTTGTAGGTGCATGATGTACAATGCACTTTTCCTCAGATGAAATCCAGG

TGATCGGAGTATGAACACCCCCACGCACACCCAAAAAAAATAAAAAAAATAAAAAAAAAAAAAAAAAAAAAAA

>CepNAC34

TTTTGAATATCGCAGACCCAGTTTGACTAAACCCGAATAAAAACACAGCAACCAACCCTAGTTTGATCCAAACAAAAATC

AATCCTGTTTCCTTCTCGATAATCTACTGATCCGGGAATGACAGGACCATCATGGTTGATAGATGGCAAAAGAATTGCAA

CAAAGATTAAATATGCATCTGAATCAAAGGATCCTAACAGAGTAAATTGGATAAGCAATCCAACAAAGCCATGCCCAAGT

TGCAACCATATTATTGATAATGATGATGTAGTTCAAGATTGGCCAGGCTTACCTGAAGGAGTGAAATTTGATCCATCTGA

CCAGGAATTAATTACTCATTTGATCGCGAAATTAGAGAAAGGAGATGAGAGACCTCATCCTTTCATCAATGAATTTATTC

CTACCGTTGATGAAGATGATGGAATATGTTATACACACCCTAAAAAATTGCCAGGAGTGAAGCTTGATGGGAGTGTATCA

CACTTTTTTCATCGCACTTTCAAGGCATACAACTCTGGAACCCGGAAACGTAGAAAAATACAGCACAATGGTGCTGATGA

TGTGCGCTGGCACAAAACTGGAAAAACCAAGCCAGTCATTGTAGATGGCAAGCATGTAGGATGTAAAAAGATTATGGTTC

TTTATAGCTCTTTGAGGAATGGAGAAAAGCCTCAAAAGACCAATTGGGTTATGCACCAGTACCATTTAGGTACAGGAGAG

GATGAAACTGATGGTGAATTTGTTGTATCGAAATTATTTTACCAGCAGCAGCCTATGTTGAAATCTTGTTCCAACGCCAA

CAAATTTGAAGTGGTGGAAGAAGCGTCACCGGTGATGGAAACTATGGATAATGATGCTCTTCCTGTTCCTCCATCATTAG

ATATTTCCCACGCTTTCAGTGTGCATGATAAATCTGCTATGGTAGCAGTGTGTTCACCTCTAGATAACGATCAGAATTGT

GTACAAGAATCGGTCACAACCGATAGTGGCACCATCATAAACGACTCCACAAGTGACAAAATACTAAAGCAAGGGAACAT

TGCTGATCAAGATAATGAAAATGGAACTGAGGCAACAGAAGACCCAAAATGGTGGGAAGGGGAGTCACAATTTCTATTAG

ATTCTCAACAGCTGGCCGAAGGTATTGCACTGTGCGATGAATTTCTTCATAGCCAAAGCCAGACATCATGCGGCGGTGAG

ATTGAAGAAGCAACAAAGAGAATCAAACCTTGTGCCTTGTCTGAATATGCTAAGAAAGGAGGCGTTGATGTGTTCAAAAA

AGATTTAGAAGAATGCCAAATTCTCAATGGTGATCATTTTGCTACTGATGATTCACAGCCGCAGTTGCCGAATTTTGAGC

TGATGGATACCCCTCCCGATTTCAGACTTAGTCAACTTGAATTTTGATCGCAAGATAACTTCATCGCGTAGTAGTTTTAT

GTATTTGTGACAAGGGGGCTGAATGAGGGTGATACGTTTTGTCTAAGCTATATGTATTTATGTAAACGCTTGTCTGATTT

TGAATTTGAATGACTATAATTTCTGTTGATCCAGAAAAATCTGTGATATGCAGTTTATTAAGCACTATGAAATAAACAAG

TTTTATGTAATGAACCAATGATTTAAAACAACATTTTCTGCGTACGGG

>CepNAC35

CTAACCTTCTCTTGTATAAAGTTTTAACAATGGGAGATCGCAATCCAAATGATATACCCCCAGGTTTCCGATTTTACCCC

TCTGACGAAGATCTTGTGGTTCACTTTCTTGCTCGAAAAGCCTCACACCTTCCTTGTCAACCCAACATCATTCCTACTCT

TGATCTCCATAATTTTGATCCATGGAGTCTTTATGGTAATGCTCTTCAAGGAGGCAACCAAGTGCATTACTTCTTCGCTC

GCAACACCCAACATAGAGCTACACCAAGCGGGTACTGGAGCCCCATCACCGTTGATGAATCCATAATGAACAGTGATCAA

GTTATCGTGGGCTTTAAAAGAACTCTTATGTTCTATTTCGGCGATATGAAGACTAATTGGGTAATGCATGAATACCATTT

ATCAGATGCTATCGTTAACAACACTAGAAGATCTTCCAAGAAGAGGGGAACTTCAAGAATTGAAATCAACAAATGGGTGA

TTTGTGGAGTATACGAATCATCATGTAATTCACATTCGAACCATAACGATGGAGGACGAGAGCTTTCGTGCTTGGACGAA

GTGTTCTTATCATTTGACGATCTTGATGAAGTGAGCTTGCCAAATTAGAACATGGAAGAAGTGTTAATAAAAGAACTAGT

CGGCCAAAGCAAGCTAACCATGTAATGTAACCACTAATTGTTCACTTGAGGCTTGTTTTTGTCCATAGTTTTATCTGAAT

ATAACTGATGTTATCTCCTAAATGCTAAAAAAGACAACAACAGTTATATTCATTCCACAGTCAAACTGCCTCAACACTCT

GT

>CepNAC36

GGAAACTTTTGCATTGAAAGAGTACTTTTCTCTCCCCACAAGAATTTGTCATTTGAAGAAGAGATAAACAAAATAAAAAA

TGAAAATCCAGTTGAGTATAGTTTCCGCCATTGATTAACAGGTCTCCCTCTTCTCCCCGCAACCAGTAATAATAACTATA

TATATAACTCACCTCCCTCTTCTCCCTCCTTAGAACAAAGCTTCCTTCTTCCCTCACTCTCTCTCTCTCTGAATAAACAT

TTATACATTTAGAGGTAGAGGTAGAAAGAAGTGAAAAAGAGTGGATACAATTTTGATATGGAGAAACAAAACCCAAGCCC

AACTCCATGTATTTCCAGGCATAGTAATACTATTATTATTAACGGAGTGATGAAAGTACCCCCTGGTTTTAGGTTCAAGC

CTACAGATGAGGAGCTTGTACTCCAATACCTAAGAAGGAAAGCCTTTTCATACCCTTTGCATTCATCCTTCATCCCCGAA

ATCGATCTCTCTAAACACAACCCTTGGGATCTCCCAGATGGTGGATGTGAAGAAGAGAGGTATTTCTTTGTACTTAGAGA

AACAAAGATCGCAACTGAGAGTGGGTACTGGAAGGCAACTGGAAAAGACAGGGTGGTTGTAGCGACCAAATGTGGAAGAG

CAGTTGGGTTAAAGAAAACGCTTGTATTTTATAAGGGGAAAGCCCCTAGAGGGCAGAAGACTGATTGGGTAATGCATGAG

TACAGTCTAGCCATTTCCAACATTTTTTCACAAAGGAAAAGCTTATCCCTTCATGGTGCAGTTGGGATAAAAGATTGGGT

GCTTTGTCGTATTTTCATGAAGAAAAGAAGTGGAATAAGCTACGTCAGGGGCCTTCCCTCGCCTGCTCATTCAGATTCAA

GTTGTGTTACGAATCATGCAGTTGGAAAATCTAATGACGGAGAGGAAATTTCCTCATTGTTACAATGAGGTCAATGGATT

GAGTAAATTATTATGTTAGTAGCTACTTTGTAAGAAGGAATAACCTTTCAGCCTTTTTCTTCTTACTTAAACGCTTAGGG

ACTTGTACACCCTCCATGCTTCTTCTTCCCTTGTTATTTTATGGTGCTTATTCAAAATGTTTTATCTCTATTGTTATGAT

CGTGTTATTTGTCAGCCACATAAATCTATATATGAGTAACTATTACTGTGTG

>CepNAC37

GCAAACGAAGCTACCCTGATACTCTACAATCCTCTTCATTTTGCCGACCTTTAGTTAAAAATAAAAATAATATTTTTTAT

TTTCTCGTCTGAAATCATCCTATTCGACTTCATCTTTTTCTATTTTGAAATAATTTAATCAGTTACTCAATAAAATTCTC

ATCTATTTACTTTTTATACAATAAATCAGATAGATACTCAATGTTCTCCGAATCATCGTCGAATTCGACCAAATTAGCAC

CAGGATTCCGATTCCATCCCACGGATGAGGAGCTGGTGAGCTATTACTTGAAGCGTAAGATATCAGGCTTGCCTTTACGC

GTCGATGCGATCGCGGAGATCGATTTGTATAAGAAAGAGCCATGGGAGCTGCCCTTTTTTTCGAAATTGCAAAGCCGCGA

CGTGGAGTGGTACTTTTTCAGCCCTTTAGACCGGAAGTATTCGAATCGGACGCGTACAAATAGGGCTACTGTCGAAGGGT

ACTGGAAAACTACGGGCAAAGATAGGGTAGTGAAGCACGCTTATTCCAATTCGGTGGTTGGCATGAAGAAGACACTTGTT

TATCATCATGGACGGGCGCCGAAAGGGAAAAGGACTAATTGGGTGATGCATGAGTATAGATTGGAGTGCTCGGAGGATGA

TGCTAATGGACGTAATTCTACTACTCTTGTTCGTCCTCAGGATTCCTTTGTTGTCGTTAGAATTTTCCAAAAGAATGGTT

CTGGACCACAAAATGGTGCTCAATATGGCGCTCCTTTTATCCCAGAAGAGTGGGAAGAGCTTCAAGGTAATATTGCTCCA

AAAGAAGAGGAAGAAAATGCACGTCACACAGTGTTGGTGCATCAAACTAATGTTCAGCACAGTGATAAAGCTTATGTTGA

ACAAAAGAACCCATTGCTGGTGGATCCACAGCAAAATACTGATCATTTATCGGCAGAGTCAAAAGATGGTCAAAGTCAAC

CAGATGACCAGACTCAAATCCATGTTGCAGGCAATCATTTGGCTGAGTCTGAGGGTCAAGGTTGTGATAGTCATGCAGAT

GATTTTACTTTTATGCTTGAGGATATCTTTGACTTAGACAAGCTTAATAATGTACCTGAAGATATGCAAGAAAACGACTC

AAGTATTGCCAATAACATGGTAGAAAACTCTGCCAATGTATCAGATCAGAATGATTGGTATCTGGAACTAAATGATATTG

CGAATGCAGATGGACTTTGCACAACCAATAATGTTTTTGATGATACTATCATATCACATTTTCCAAATGAAGGTAGTGCT

TCACAAGAATTAGATGGATCTGAGGTCTTTGATAATTCTAACAGAACCATGGGTACTGAAGGCATGTTTGAGATGCAGCA

GTCTGATGATTTTCTTAATCCTGCAGTTGCTAGTCGTCTTGGAGATAATACTGTTTTTTATGATGCACCTAGCAATGATA

CTCCATACACAGGAGATGTGTACATGGACATGAATTCAAACGATCAGTTTTCACCTTATATTGGAGATTTTGATTTGGTG

GATGACTTAATGGCTTACTTTGATGCATCGGAACATGATTTACCATATGAAGTTGACTCTCTCGGCTCATACAGTTCAGA

TATTACCCATCCTAATTTTGCAGCAAAGGTTGAGGGGAGTAGCTACCCTACGATTAAGCAAAACCTTGGGGACCCTGAAA

CAAATATTGGAAAGGGTGCATCATCTTCAATATTTACTAACGCCACCAATCCTTTGGAAACCACAAAAAGGAATAGCATC

GCTGCTACAGATGTGCAATCAAATGATGGTTGGAACAAGTCTTTCAAAAAGCATGTTGCAGACATGCTAGATTCAATTTC

CGCTCCACCTGCTATGGCTGAAGAAACATGCAAAGGAAAAGCTGCTCAGATATTCAACGGCAGCTCTTTGATTCATGCTT

CTGCTGGCATGATAAAGATTCAAGGTGTATCAGTGAATGGAATCCCAGAAAACTGGACCTTAAATAAGAATGGAGAAATA

AGCTTCATCTATACTTATGCTAACTCTAACGATGCAGTTGAAAAATCACCGTGCTTTAGGCCACTTACTGCTAGTGCAGC

TTTCAGAATCTTGCTTAGAAGTGGATTATACATTTTTGGCATCGCTGCTGTCCTAATTTCTATAAGCCTGAAACTTGGAA

CCGGTATTTATAGTAACTAAACGCCTTTCTAATTGGAAACTCCTTTTGGTTGAATAAATGCAATGGCTTTCTTCTGTAAT

ATTTCTTCATGTATTTTGTGGAGAAAATTGGTGCGTGGATTTATGTTGTCTTTCTGCTGCCTGAAAAACAACTTGCAGTA

AAAATCTGATTTTTATGTTGCTTTGACGTTTTAAGCTGCCGTGGTACAAATTTTTGGTGCTCATAAGAGGTGCATGCGTA

CGATGACTATTATATTCAAGTGTGGTACTTGACATTGTAATTTGATGTGTGGGCAATATTACTGCTTTGTGATAGTCTCC

TTTTGCACAGAGGTTGTCAGTCCCTTTTTTAACTAGTTACATGCTGTGCTGCAAATGTCATACAATGTTATTCCTGTAAC

ACAGATATTAGAAACCA

>CepNAC38

TAAATTCAATTTTAATTAAAGGGTTTCTCGGTTTTTGACATTTCTTGTTGTCTACTCATGTCACAAATCCAATTTTACTT

GAAGCTTTTTTAATACTGAAAGGAAGATAAGAAGTTTTATTTCAAAGATTTCAAAGGTAAGGTGTATAATCTTTTCCTTC

CAATTATTATTCAAATCCAAATCCCAACCTTTTTCATATAAATTTTTCACAAGCTCCATTTCCGATGAATTCAGTCCACT

TCCCCAAATCAAAGAACTAGCCTTTTCACAATCGAAACCCTAATTCTGAAATTCGATGAATCTGCTAAACCCAACGGCCA

ATCTGGCCCCAGGCTTCCGATTCCACCCAACTGAAACGGAGTTGATCACTTTTTACCTTAATCGTAAAATCAACCGCGTG

CCGATTGAGTTCAACCCGATTGCTGACATCGATCTTTACAAATACTCGCCGTGGAAGCTGCCTGAGCTGTCAAATTTAAA

GATACGGCATGAAAAGCAGTGGTTTTTCTTTAATAGCTTGGATAGGAAGTACGATGGCTCTAGAAGGTCGAACCGAGCAA

CGGCTGACGGGTTTTGGAAGTCGACTGGGAAAGACAGGCCGATTTACGAGGAGACGGAGAGTAAAAGGATTATTGGAATG

AAAAAGATTTTGGTTTTCCATGTTGGAAGAGCGCCAAATGGAAAGAGAACTGACTGGGTGATGCATGAGTACAGAGGTGT

CGAGGATGCGGAGATTAAAGACAATAGGGCTTCCTTTGTCCTTTGCAGAATATTCCATAAAAGCTCGATTGGAAGGGAGA

AAATGGATCAGTGCGGATCCCGCTATGTCAAGGAGGAGTGGGAAGATGCTCATGATTCAAGTGAATCTGATACAAATGGC

TTGGTAGATGCTGATGGCTCTGACCAGAAAACAGATGTGATCTTTTTGGCTCCTGGTTTTCGATTCCGCCCTACTGATGA

AGAGCTTGTTGGTTATTACTTGAAGCGTAAGGTTATGGGTCTCCACCTAAGAGTCGATGCCATTCCCGAAATAAATATAT

ACAAATCAGCTCCTTGGGACCTACCTTCCCTTACTCGCGTTAGCCTAACTGTACCTAACAAGGAGGGCTATTTCTTTAGT

ATCCTTGACCGTAAGTATTCTCAAAACCACTCCACAATTAACAATCGTGCAACTGCAGAGGGCTACTGGAAGTCCACCGG

CAATGACTGCCCCGTACTTAGCCACTTAGGAGGGAAAAAAATGGGGATGAAGAAATTTTTGGTTTTCCATACTGGTCGAG

CCCCACGCAGCCAAAGAACTGATTGGGTGATGCACGAGTACCGTTTAGAGGAAGATGAACAAAACTCTGTGATGTGCGGA

AAAGGGTCAACCTTTGTCGTGTGTAAGATATTTCAGAAAAAGAGTTTTGGTGAGCAGAAGGGCCGACCTCGTTCCGTGGA

TCCCACCACTGAAGAAACAGATGAGGGGGAAATAGTTCCTGTAGATGTTTTAGATTGTGCTCCGAACCCCACAACTGATG

GTTCTCCGAATGTCACCACAGCAGTTTGTACTCCTATAGTCAATGAAGCAATGTTTGCTCCTATGGTCACTGAAGAAAAT

TTTGCTCCAAAAGTTACCCAAGCACATGCTGCTCAAAATGCCGAACTGATAGAGGTTGTTCAGAAGAAAATTCATGCCCA

AGCCTCCAAAGAGGTTCAACAGGGAAAAGCACAGAGTCTGTACAAAGTTGGCAATCATGTAGAAATCGCATATATGCGCC

AACAAATTCCAGTTGCTTGGTACACAGCATCAGTGATAAAAGTACAAGACGAACAAGTCCTATTAGTCGAGTATGAAACT

TTGAAGGCTAAAGACAACTCATTGTTGAGTGAATTAGTTTCCATCCAATACATAAGGCCACATCCTCCCGTTCGAGAAGT

TGAACATTTTAAAGTGCTTGCAGAAGTAGAAGCTTACTATAATGGTGGATGGTGGCCAGGAGTTGTTGCTACCGTGCATG

ATAATGCAAAGTATAACGTAAAATTCATGCATTTGGAAGAAGAAATTGAATTTGATCACAAGGAGTTAAGGTTACTCTAT

GACTGGGTTGATGGGAAATGGGTTCAAGCATCGCAGGAAAACAAAACATTAGAATCACTGAACCATGAAGAATTGAGGAC

TGTTGAACATACACAGTTGTTGCATAGTAATAATCAAGATCATAAGCGCAAACTTTCTCATTCAAATGGTTGCGAGATCG

GTATTACTTCTGAATTTACTATTACTTCAAATATGGAGAATAAAAATGGCAAAGAAATAGCAATCAAAAGTCAACATGAG

GTTTTTCAGCAATTCAAAAAAGCTAAAATAGAAGAGAATGTAGAAGATCCTTTTAGTGAAGAGAACAATGAAGAACTTGT

TCTGGAACTCAGTCTACCAGGATACAAGGGGGGAAAAATACATGAAGATAGAAAGAAGAAGAAGGATAACTGCATACCTC

GGACAGTAGAATCTTCATTAAATGTAGGATTACTGAGCAAAATTCAAGAAAACAATAAATCGCTTGAATTGTCAACAAGT

AGTGATAACAGCTCTGAAGCACAAGGACTATCCAATCCCTTAACCAGCTCTTCCGATGCAAGCTTAGTCGAATCTTCGAC

CTCTAGCGTCAAACCAAGGTTCGATAAGCTTCCATTGGAGCAACCCTCTTTACCCTTCGAAAAGCACTCTAGTTTATGGG

ATACACTCGAAAACATGGAAGTATTCCACACAATGCCGCAAAATCCCCATTTCGCCCCTTTAGGACAACATGGTGAAGCC

TTCCGTGAAGGAATGGCCATAGGGCTAATGTTGACATTTTCTAATGTGGTCGCCGGTATAGAGAAACTGGATGCAACGGA

CTCTGTAGAAAAGCTTGCTCTGATATACAAAACGCTCATAGAGCTCGAAGCTCATGGTTTCGAGGTGCAATGCTTGAGAG

CTCGTCTTATGGAGTTGTTGAAGGTGAAGAGGAATAAAGAGAAGCTTGATGATGATGTTTCTAGTTTGGAGGATTTGATT

GAAAAAAATGGAAGGGTTGATTTGAATTTGAAGTTGGATTTGGAGGTGAAAAGTGATGTTTCTTCTTGAATATACATGAA

GAGATGGGCATTTGGTAACTTTTATCGTCATGTTATGCAGATATGTATGTATTTTGTAATGTTAAGTTATCCTTTCTAAG

TTATCCTTTTGTGTAATGTCTTAAAAAACATGCATGTTGGTTTAAATTTTATGGTGGTATTTAGCTCTTTCAGTCTCATG

TATACATAGTAAGATGTATTATCATGGTATTAGAAGAAAGTGTTATGCATTTCCAGCTCTGAATCTTGTAAATTGTCATA

AAGTACAATTTTGATATTTTAATAGCAGGAAAGATTTATATGGGTTGTGCTTTT

>CepNAC39

AAAAAGGATCAGCATTTGAAAAGAAGCATAGATGGAGAGCACGGACTCATCCACAGGCCCACCAGCACCGCCGCAGCCAC

ATCCACAGTTGCAGCCTGCGCAGCCGCAGCTGCCGCCCGGATTCCGGTTCCATCCAACCGATGAAGAGCTGGTAGTCCAC

TACTTGAAAAGAAAAGCCGCCTCTGTCCCTCTTCCTGTGTCCATCATCGCTGAGGTCGATCTGTACAAGTTCGACCCCTG

GGAACTTCCAAGCAAGGCAAGCTTTGGGGAGCAGGAGTGGTACTTCTTCAGTCCAAGGGACAGGAAGTACCCTAACGGAG

CAAGGCCCAACCGAGCAGCAACATCTGGATATTGGAAGGCGACAGGGACAGATAAGCCGATAATGAGCTCCATAGGTGAT

TCTGGAGGCTGTTCGGTGTCTGCTGGGCAGATGAAGAAGGTTGGTGTGAAGAAGGCCCTGGTTTTCTATGGCGGGAAGCC

TCCGAAAGGGATCAAGACTAACTGGATCATGCATGAA
