# Supplementary material for: Identification of an NAC Transcription Factor Family by Deep Transcriptome Sequencing in Onion (Allium cepa L.)
Source: PLoS One. 2016 Jun 22;11(6):e0157871. doi: 10.1371/journal.pone.0157871 (PMC4917099; doi:10.1371/journal.pone.0157871)
Supplement: S1 Table — (DOC) [file pone.0157871.s003.doc]

Table 1 Details of the 47 functionally characterized NAC genes previously reported in other species.

| Gene | Other name | Group | Species | GeneBank ID | Function | Reference |
| --- | --- | --- | --- | --- | --- | --- |
| NAC1 |  | II | Arabidopsis | AT1G56010 | Promote lateral root development | Xie et al. 2000 |
| CUC3 |  | II | Arabidopsis | At1g76420 | Establishment of meristem boundaries | Vroemen et al. 2003 |
| GRAB2 |  | II | Wheat | AJ010830 | Resistance to wheat dwarf geminivirus | Xie et al. 1999 |
| ONAC45 |  | II | Rice | AK067922 | Regulating abiotic stress response | Zheng et al. 2009 |
| CUC2 |  | II | Arabidopsis | At5g53950 | Controlling organ separation in shoot and floral meristems | Ishida et al. 2000 |
| CUC1 |  | II | Arabidopsis | At3g15170 | Controlling organ separation in shoot and floral meristems | Ishida et al. 2000 |
| ORE1 | AtNAC2,ANAC092 | II | Arabidopsis | At5g39610 | Regulating senescence | Balazadeh et al. 2010 |
| ORS1 |  | II | Arabidopsis | At3g29035 | Regulating senescence | Balazadeh et al. 2011 |
| TIP |  | III | Arabidopsis | [AT5G24590](http://www.arabidopsis.org/servlets/TairObject?type=locus&name=AT5G24590) | Resistance to turnip crinkle virus | Ren et al. 2000 |
| NTL6 |  | III | Arabidopsis | At3g49530 | Regulating drought stress response | Kim et al. 2012 |
| FSQ6 | ANAC089 | III | Arabidopsis | At5g22290 | Negatively regulating floral initiation; suppressor of fructose signaling | Li et al. 2011 |
| NTL4 |  | III | Arabidopsis | At3g10500 | Regulating senescence | Lee et al. 2012 |
| NTM1 |  | III | Arabidopsis | At4G01540 | Regulating cell division | Kim et al. 2006 |
| NTM2 |  | III | Arabidopsis | At4G01550 | Integrating auxin and salt signals to regulate seed germination | Park et al. 2011 |
| RIM1 |  | III | Rice | AB265821 | Resistance to rice dwarf virus | Yoshii et al. 2009 |
| ENAC1 |  | III | Rice | AK071274 | Regulating abiotic stress response | Sun et al. 2012 |
| ANAC036 |  | III | Arabidopsis | At2g17040 | Regulating cell growth | Kato et al. 2010 |
| VNI2 |  |  | Arabidopsis | AT5G13180 | Integrating stress Signals to regulate senescence | Yang et al. 2011 |
| senu5 |  |  | Tomato | Z75524 | Regulating senescence | John et al. 1997 |
| XND1 |  |  | Arabidopsis | At5g64530 | Regulating secondary wall synthesis | Zhao et al. 2008 |
| PtrWND1A |  | IV | Poplar | XM_002317023 | Regulating secondary wall synthesis | Zhong et al. 2010 |
| PtrWND1B |  | IV | Poplar | XM_002300464 | Regulating secondary wall synthesis | Zhong et al. 2010 |
| NST3 | SND1, ANAC012 | IV | Arabidopsis | At1g32770 | Regulating secondary wall synthesis | Mitsuda et al. 2007; Li et al. 2012 |
| NST2 |  | IV | Arabidopsis | At3g61910 | Regulating secondary wall synthesis | Mitsuda et al. 2005 |
| PtrWND2B |  | IV | Poplar | XM_002302636 | Regulating secondary wall synthesis | Zhong et al. 2010 |
| NST1 |  | IV | Arabidopsis | At2g46770 | Regulating secondary wall synthesis | Mitsuda et al. 2007 |
| VND6 |  | IV | Arabidopsis | At5g62380 | Regulating secondary wall synthesis | Kubo et al. 2005 |
| PtrWND6B |  | IV | Poplar | XM_002325955 | Regulating secondary wall synthesis | Zhong et al. 2010 |
| VND7 |  | IV | Arabidopsis | At1g71930 | Regulating secondary wall synthesis | Yamaguchi et al. 2011 |
| TaNAC2 |  | V | Wheat | AAU08786 | Regulating abiotic stress response | Mao et al. 2012 |
| SNAC1 |  | V | Rice | AK067690 | Regulating abiotic stress response | Hu et al. 2006 |
| OsNAC4 |  | V | Rice | AB028183 | Regulating biotic stress response by plant immune response | Kaneda et al. 2009 |
| GRAB1 |  | V | Wheat | AJ010829 | Resistance to wheat dwarf geminivirus | Xie et al. 1999 |
| OsNAC5 |  | V | Rice | AB028184 | Regulating abiotic stress response | Takasaki et al. 2010 |
| OsNAC52 |  | V | Rice | AAT44250 | Regulating abiotic stress response | Gao et al. 2010 |
| SNAC2 | OsNAC6 | V | Rice | AB028185 | Regulating abiotic stress response | Nakashim et al. 2007 |
| GmNAC2 |  | V | Soybean | AY974350 | Regulating abiotic stress response | Jin et al. 2013 |
| ATAF1 |  | V | Arabidopsis | [AT1G01720](http://www.arabidopsis.org/servlets/TairObject?type=locus&name=AT1G01720) | Regulating stress response | Wu et al. 2009 |
| ANAC072 | RD26 | V | Arabidopsis | [AT4G27410](http://www.arabidopsis.org/servlets/TairObject?type=locus&name=AT4G27410) | Regulating stress response | Tran et al. 2004 |
| ANAC055 |  | V | Arabidopsis | [AT3G15500](http://www.arabidopsis.org/servlets/TairObject?type=locus&name=AT3G15500) | Regulating stress response | Tran et al. 2004 |
| ANAC019 |  | V | Arabidopsis | [AT1G52890](http://www.arabidopsis.org/servlets/TairObject?type=locus&name=AT1G52890) | Regulating stress response | Tran et al. 2004 |
| NARS1 | NAC2 | V | Arabidopsis | AT3G15510 | Regulating stress response and embryogenesis | Kunieda et al. 2008; Masoomi-Aladizgeh etal. 2015 |
| NAM | NARS2 | V | Arabidopsis | AT1G52880 | Regulating embryogenesis, determining positions of meristems and primordia | Souer et al. 1996; Kunieda et al. 2008 |
| OsNAC10 |  | V | Rice | AK069257 | Regulating abiotic stress response | Jeong et al. 2010 |
| NAP | ANAC029 | V | Arabidopsis | At1g69490 | Regulating fruit senescence | Kou et al. 2012 |
| mtNAC969 |  |  | Medicago truncatula | JN833713 | Regulating abiotic stress response | Ze´licourt et al. 2012 |
| JUB1 | ANAC042 |  | Arabidopsis | At2g43000 | Regulating senescence by ROS response | Wu et al. 2012 |

**Reference**

Balazadeh S, Kwasniewski M, Caldana C, Mehrnia M, Zanor M, Xue G, Mueller-Roeber B (2011) ORS1, an H_2_O_2_-Responsive NAC transcription factor, controls senescence in *Arabidopsis thaliana*. Molecular Plant 4: 346–360.

Balazadeh S, Siddiqui1 H, Allu A, Matallana-Ramirez L, Caldana C, Mehrnia M, Zanor M, hler B and Mueller-Roeber B (2010) A gene regulatory network controlled by the NAC transcription factor ANAC092/AtNAC2/ORE1 during salt-promoted senescence. The Plant Journal 62:250–264.

Gao F, Xiong A, Peng R, Jin X, Xu J, Zhu B, Chen J, Yao Q (2010) OsNAC52, a rice NAC transcription factor, potentially responds to ABA and confers drought tolerance in transgenic plants. Plant Cell Tiss Organ Cult 100:255–262.

Hu H, Dai M, Yao J, Xiao B, Li X, Zhang Q, Xiong L (2006) Overexpressing a NAM, ATAF, and CUC (NAC) transcription factor enhances drought resistance and salt tolerance in rice. Proc Natl Acad Sci USA 103:12987–12992.

Ishida T, Aida M, Takada S, Tasaka M (2000) Involvement of CUPSHAPED COTYLEDON genes in gynoecium and ovule development in Arabidopsis thaliana. Plant Cell Physiol 41:60–67.

Jeong J, Kim Y, Baek K, Jung H, Ha S, Do Choi Y, Kim M, Reuzeau C and Kim J (2010) Root-specific expression of OsNAC10 improves drought tolerance and grain yield in rice under field drought conditions. Plant Physiol 153:187–195.

Jin H, Huang F, Cheng H, Song H, Yu D (2013) Overexpression of the GmNAC2 Gene, an NAC Transcription Factor, Reduces Abiotic Stress Tolerance in Tobacco. Plant Mol Biol Rep 31:435–442.

John I, Hackett R, Cooper W, Drake R, Farrell A, Grierson D (1997) Cloning and characterization of tomato leaf senescence-related cDNAs. Plant Mol Biol 33:641–651.

Kaneda T, Taga Y, Takai R, Iwano M, Matsui H, Takayama S, Isogai A and Che F (2009) The transcription factor OsNAC4 is a key positive regulator of plant hypersensitive cell death. The EMBO Journal 28:926–936.

Kato H, Motomura T, Komeda Y, Saito T, Kato A (2010) Overexpression of the NAC transcription factor family gene ANAC036 results in adwarf phenotype in Arabidopsis thaliana. Journal of Plant Physiology 167:571–577.

Kim M, Park M, Seo P, Song J, Kim H and PARK C (2012) Controlled nuclear import of NTL6 transcription factor reveals a cytoplasmic role of SnRK2.8 in drought stress response. Biochemical Journal 448:353–363.

Kim Y, Kim S, Park J, Park H, Lim M, Chua N, Parka C (2006) A Membrane-Bound NAC Transcription Factor Regulates Cell Division in Arabidopsis. Plant Cell 18:3132–3144.

Kou X, Watkins C and Gan S (2012) *Arabidopsis AtNAP* regulates fruit senescence. Journal of Experimental Botany 63:6139–6147.

Kubo M, Udagawa M, Nishikubo N, Horiguchi G, Yamaguchi M, Ito J, Mimura T, Fukuda H, and Demura T (2005) Transcription switches for protoxylem and metaxylem vessel formation. Genes Dev 19:1855–1860.

Kunieda T, Mitsuda N, Ohme-Takagi M, Takeda S, Aida M, Tasaka M, Kondo M, Nishimura M, Hara-Nishimura I (2008) NAC Family Proteins NARS1/NAC2 and NARS2/NAM in the Outer Integument Regulate Embryogenesis in Arabidopsis. The Plant Cell 20: 2631–2642.

Lee S, Seo P, Lee H and Park C (2012) A NAC transcription factor NTL4 promotes reactive oxygen species production during drought-induced leaf senescence in Arabidopsis. The Plant Journal 70, 831–844.

Li P, Windb J, Shi X, Zhang H, Hanson J, Smeekens S and Teng S (2011) Fructose sensitivity is suppressed in Arabidopsis by the transcription factor ANAC089 lacking the membrane-bound domain. Proc Natl Acad Sci USA 108: 3436–3441.

Li Q, Lina Y, Sun Y, Song J, Chen H, Zhang X, Sederoff R, Chiang V (2012) Splice variant of the SND1 transcription factor is a dominant negative of SND1 members and their regulation in Populus trichocarpa. Proc Natl Acad Sci USA 109: 14699–14704.

Mao X, Zhang H, Qian X, Li A, Zhao G and Jing R (2012) TaNAC2, a NAC-type wheat transcription factor conferring enhanced multiple abiotic stress tolerances in Arabidopsis. Journal of Experimental Botany 63:2933–2946.

Masoomi-Aladizgeh F, Aalami A, Esfahani M, Aghaei M, Mozaffari K (2015) Identification of *CBF14* and *NAC2* Genes in *Aegilops tauschii* Associated with Resistance to Freezing Stress. Appl Biochem Biotechnol 176:1059–1070.

Mitsuda N, IwaseA, Yamamoto H, Yoshida M, Seki M, Shinozaki K, Ohme-Takagi M (2007) NAC Transcription Factors, NST1 and NST3, Are Key Regulators of the Formation of Secondary Walls in Woody Tissues of Arabidopsis. The Plant Cell 19:270–280.

Nakashima K, Tran LS, Van Nguyen D, Fujita M, Maruyama K, Todaka D, Ito Y, Hayashi N, Shinozaki K, Yamaguchi-Shinozaki K (2007) Functional analysis of a NAC-type transcription factor OsNAC6 involved in abiotic and biotic stress-responsive gene expression in rice. Plant J 51:617–630.

Park J, Kim Y, Kim S, Jung J, Woo J, Park C (2011) Integration of Auxin and Salt Signals by the NAC Transcription Factor NTM2 during Seed Germination in Arabidopsis. Plant Physiology 156:537–549.

Ren T, Qu F, Morris TJ (2000) HRT gene function requires interaction between a NAC protein and viral capsid protein to confer resistance to turnip crinkle virus. Plant Cell 12:1917–1926.

Souer E, van Houwelingen A, Kloos D, Mol J, Koes R (1996) The no apical meristem gene of Petunia is required for pattern formation in embryos and flowers and is expressed at meristem and primordia boundaries. Cell 85:159–170.

Sun H, Huang X, Xu X, Lan H, Huang J, Zhang H (2012) ENAC1, a NAC Transcription Factor, is an Early and Transient Response Regulator Induced by Abiotic Stress in Rice (Oryza sativa L.). Mol Biotechnol 52:101–110.

Takasaki H, Maruyama K, Kidokoro S, Ito Y, Fujita Y, Shinozaki K, Yamaguchi-Shinozaki K, Nakashima K (2010) The abiotic stress-responsive NAC-type transcription factor OsNAC5 regulates stress-inducible genes and stress tolerance in rice. Mol Genet Genomics 284:173–183.

Tran L, Nakashima K, Sakuma Y, Simpson S, Fujita Y, Maruyama K, Fujita M, Seki M, Shinozaki K, Yamaguchi-Shinozaki K (2004) Isolation and functional analysis of Arabidopsis stress inducible NAC transcription factors that bind to a drought responsive cis-element in the early responsive to dehydration stress 1 promoter. Plant Cell 16:2481–2498.

Vroemen CW, Mordhorst AP, Albrecht C, Kwaaitaal MA, de Vries SC (2003) The CUP-SHAPED COTYLEDON3 gene is required for boundary and shoot meristem formation in Arabidopsis. Plant Cell 15:1563–1577.

Wu A, Allu A, Garapati P, Siddiqui H, Dortay H, et al (2012) JUNGBRUNNEN1, a Reactive Oxygen Species-Responsive NAC Transcription Factor, Regulates Longevity in Arabidopsis. Plant Cell, 24: 482–506.

Wu Y, Deng Z, Lai J, Zhang Y, Yang C, Yin B, Zhao Q, Zhang L, Li Y, Yang C, Xie Q (2009) Dual function of *Arabidopsis ATAF1* in abiotic and biotic stress responses. Cell Research 19:1279-1290.

Xie Q, Frugis G, Colgan D, Chua N (2000) Arabidopsis NAC1 transduces auxin signal downstream of TIR1 to promote lateral root development. Genes Dev 14:3024–3036.

Xie Q, Sanz-Burgos A, Guo H, Garcia J, Gutierrez C (1999) GRAB proteins, novel members of the NAC domain family, isolated by their interaction with a geminivirus protein. Plant Mol Biol 39:647–656.

Yamaguchi M, Mitsuda N, Ohtani M, Ohme-Takagi M, Kato K and Demura T (2011) VASCULAR-RELATED NAC-DOMAIN 7 directly regulates the expression of a broad range of genes for xylem vessel formation. The Plant Journal 66, 579–590.

Yang S, Seo P, Yoon H, Park C (2011) The Arabidopsis NAC Transcription Factor VNI2 Integrates Abscisic Acid Signals into Leaf Senescence via the COR/RD Genes. The Plant Cell 23:2155–2168.

Yoshii M, Shimizu T, Yamazaki M, Higashi T, Miyao A, Hirochika H, Omura T (2009) Disruption of a novel gene for a NAC-domain protein in rice confers resistance to Rice dwarf virus. The Plant Journal 57:615–625.

Ze´licourt A, Diet A, Marion J, Laffont C, Ariel F, Moison M, Zahaf O, Crespi M, Gruber V, Frugier F (2012) Dual involvement of a Medicago truncatula NAC transcription factor in root abiotic stress response and symbiotic nodule senescence. The Plant Journal 70:220–230.

Zhao C, Avci U, Grant E, Haigler C and Beers E (2008) XND1, a member of the NAC domain family in Arabidopsis thaliana, negatively regulates lignocellulose synthesis and programmed cell death in xylem. The Plant Journal 53, 425–436.

Zheng X, Chen B, Lu G, Han B (2009) Overexpression of a NAC transcription factor enhances rice drought and salt tolerance. Biochem Biophy Res Commun 379:985–989.

Zhong R, Lee C, Ye Z (2010) Functional Characterization of Poplar Wood Associated NAC Domain Transcription Factors. Plant Physiology 152:1044–1055.
